# Supplementary figures and images for: Inheritance of H3K9 methylation regulates genome architecture in Drosophila early embryos
Source: EMBO J. 2024 Jun 3;43(13):8. doi: 10.1038/s44318-024-00127-z (PMC11217351; doi:10.1038/s44318-024-00127-z)

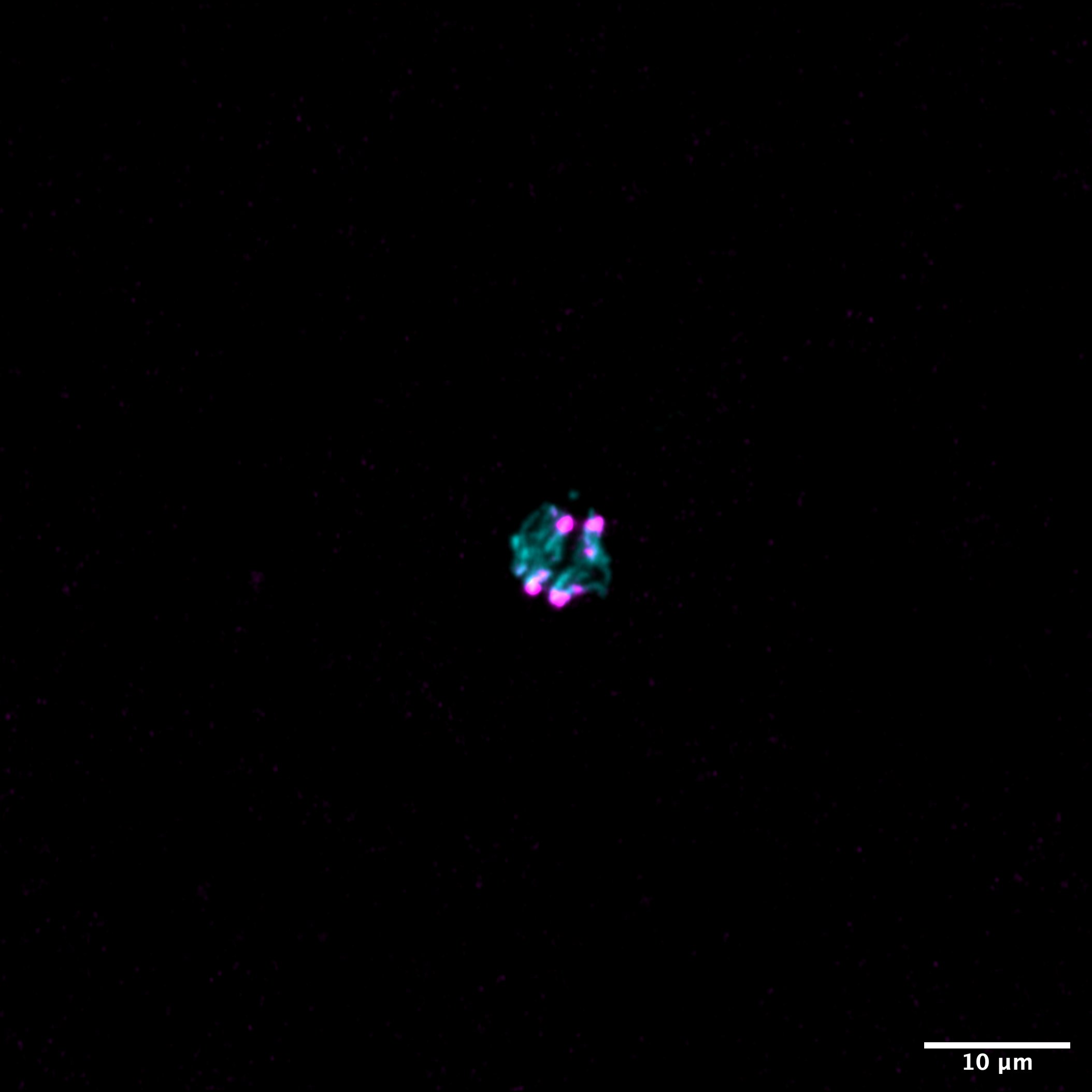

Supplement: Supplementary file 8 — Source data Fig. 1 [file 44318_2024_127_MOESM8_ESM.zip › figure1/figure1b/figure 1b_ctr_H3K9me3_DAPI_cycle4.jpg]

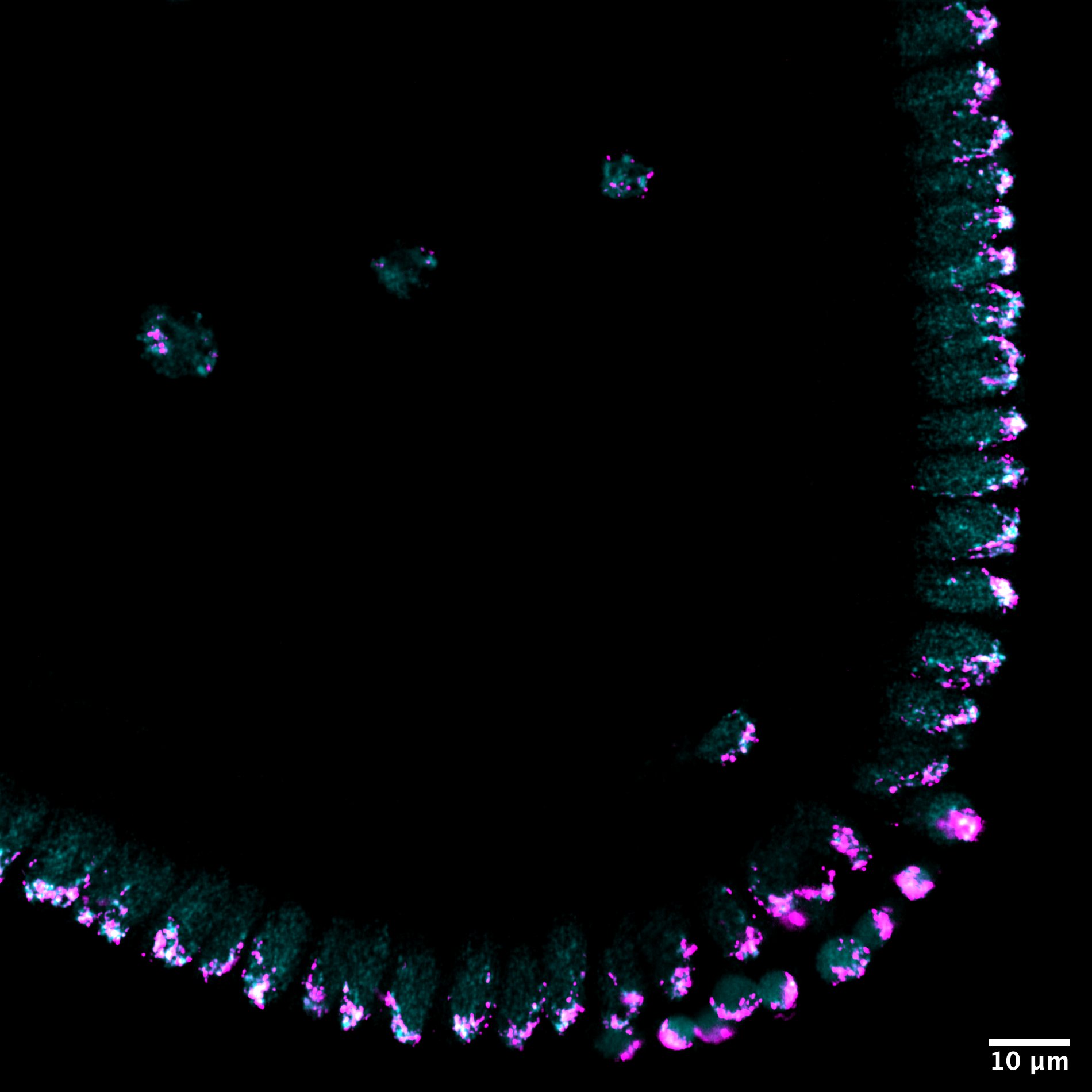

Supplement: Supplementary file 8 — Source data Fig. 1 [file 44318_2024_127_MOESM8_ESM.zip › figure1/figure1b/figure 1b_ctr_H3K9me3_DAPI_cycle14.jpg]

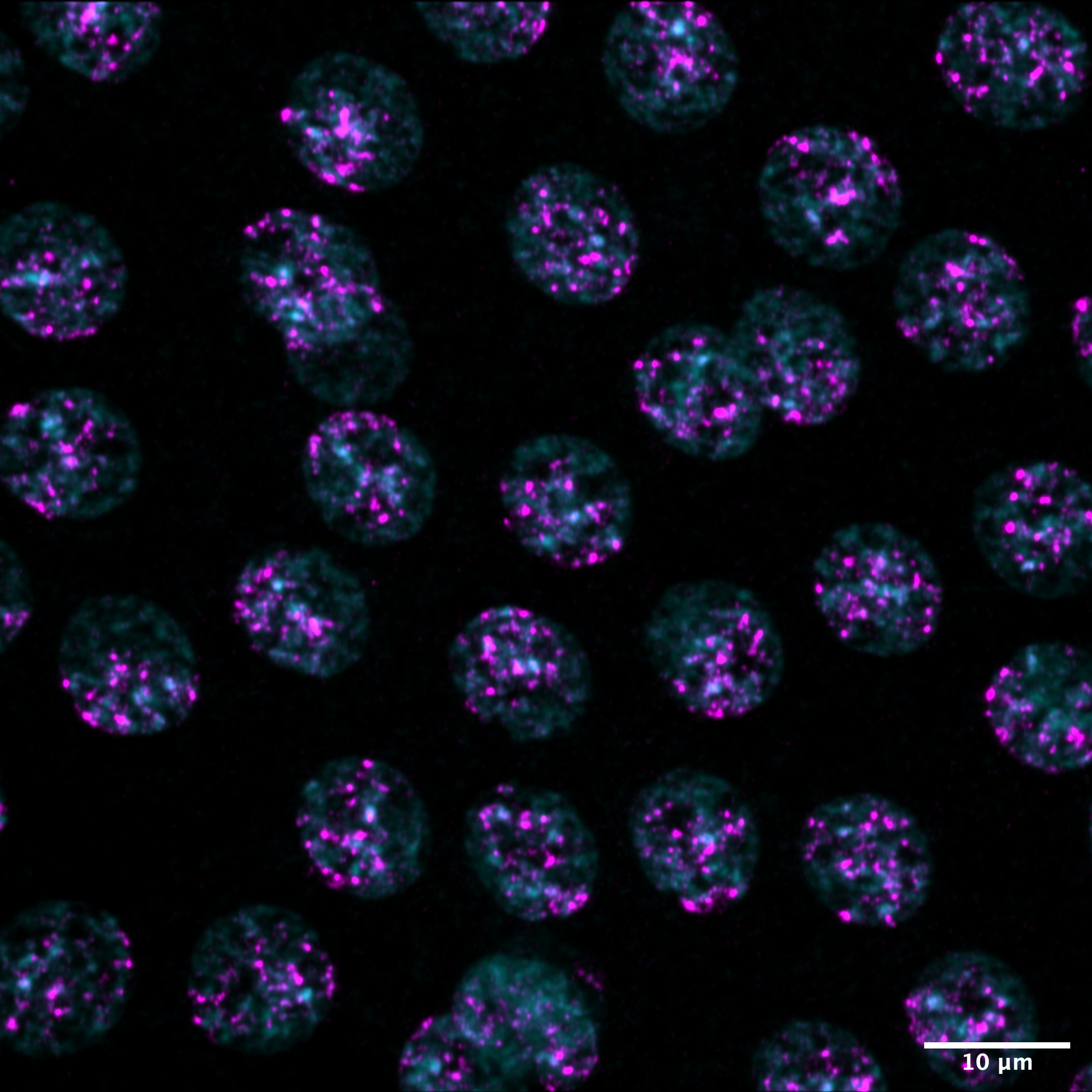

Supplement: Supplementary file 8 — Source data Fig. 1 [file 44318_2024_127_MOESM8_ESM.zip › figure1/figure1b/figure 1b_ctr_H3K9me3_DAPI_cycle9-10.jpg]

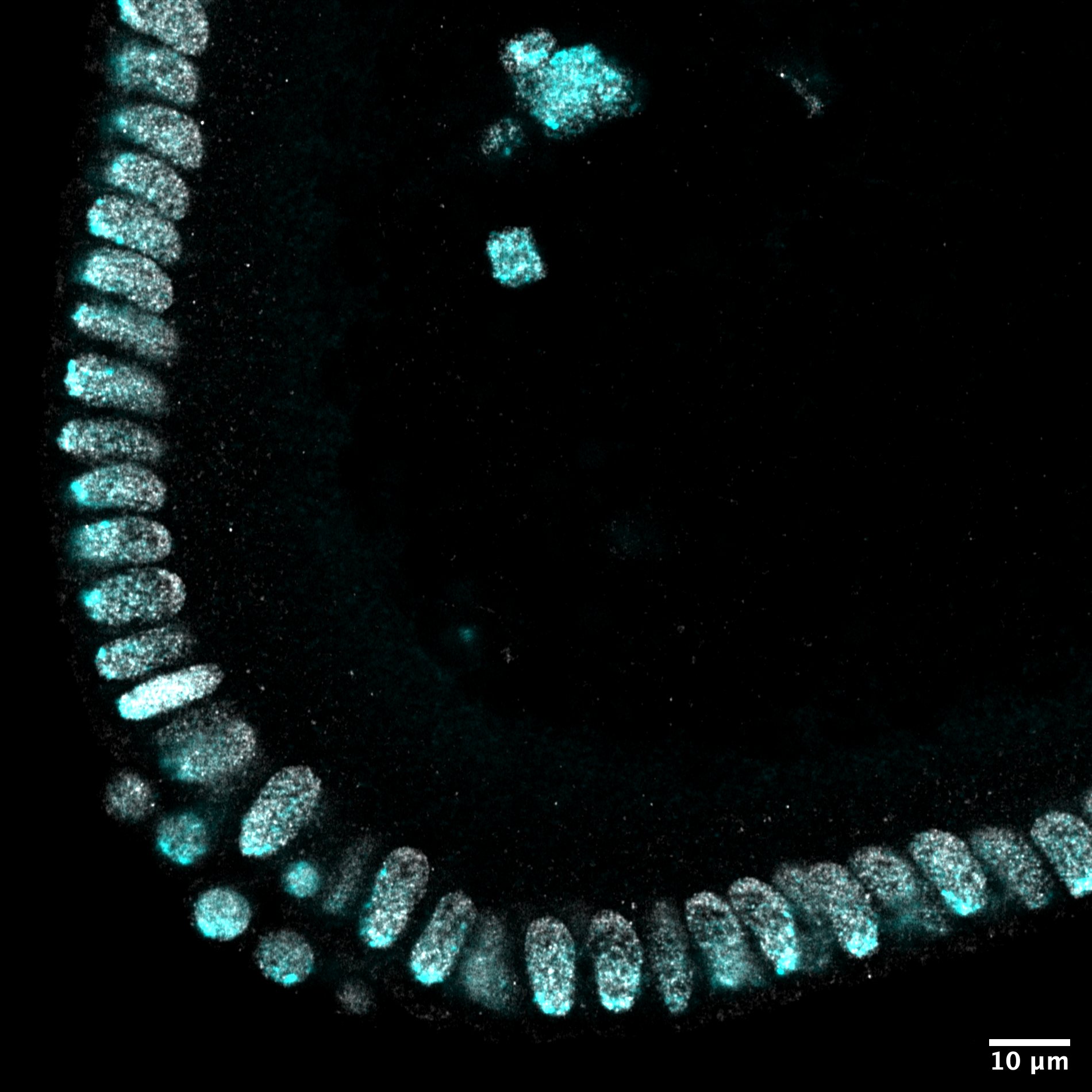

Supplement: Supplementary file 8 — Source data Fig. 1 [file 44318_2024_127_MOESM8_ESM.zip › figure1/figure1h/G9a/figure 1h_G9a_cycle 14_G9a_DAPI.jpg]

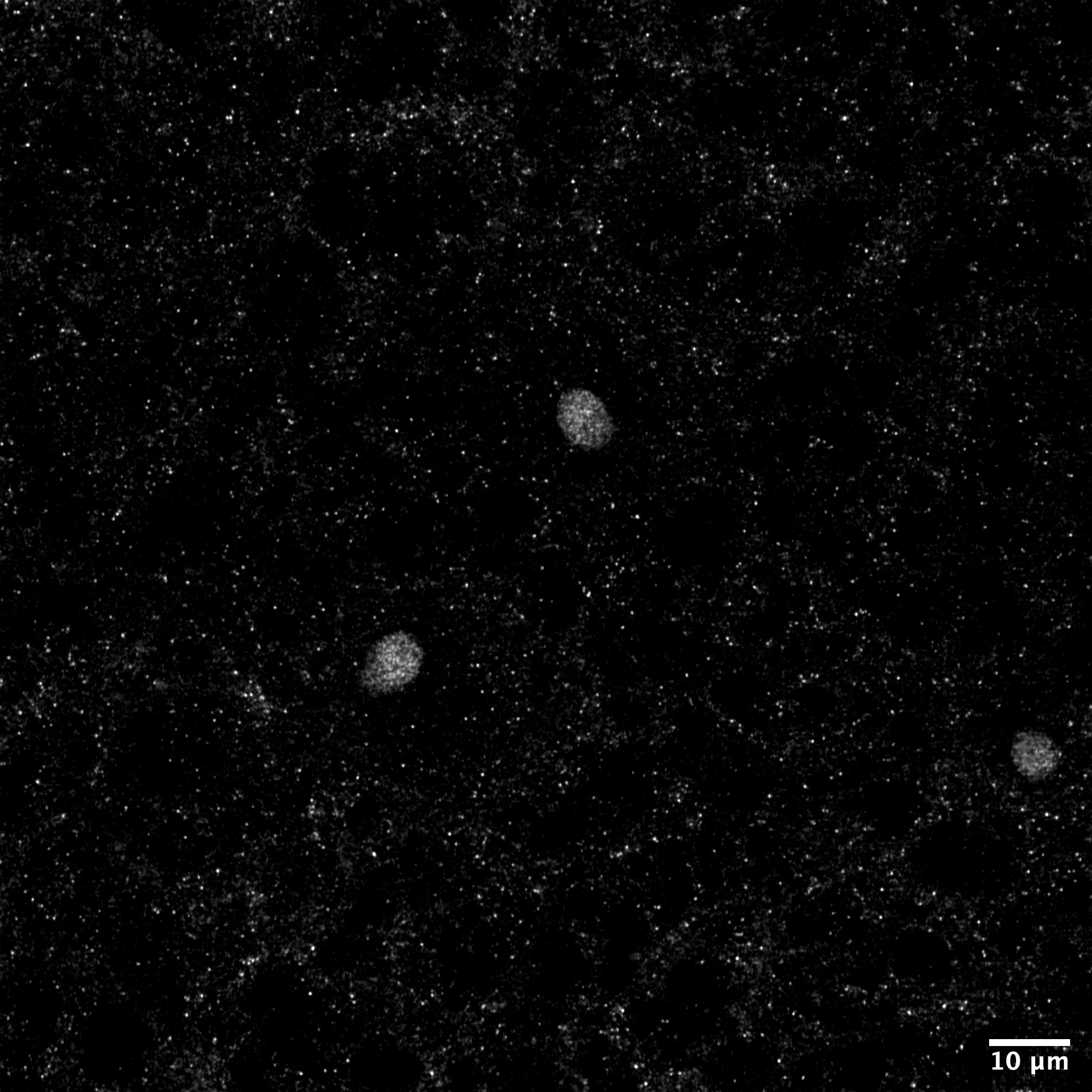

Supplement: Supplementary file 8 — Source data Fig. 1 [file 44318_2024_127_MOESM8_ESM.zip › figure1/figure1h/G9a/figure 1h_G9a_cycle 4-5_G9a.jpg]

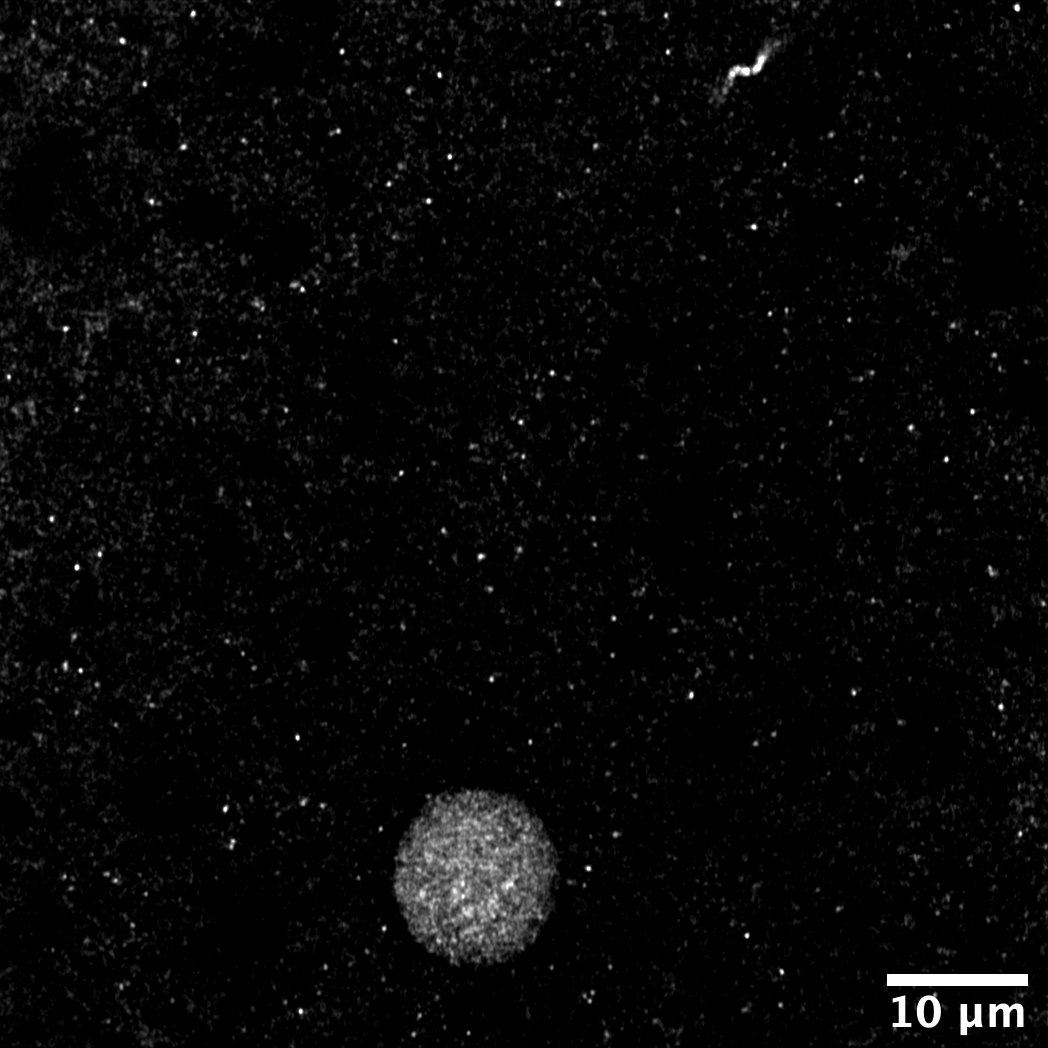

Supplement: Supplementary file 8 — Source data Fig. 1 [file 44318_2024_127_MOESM8_ESM.zip › figure1/figure1h/G9a/figure 1h_G9a_cycle 1_G9a.jpg]

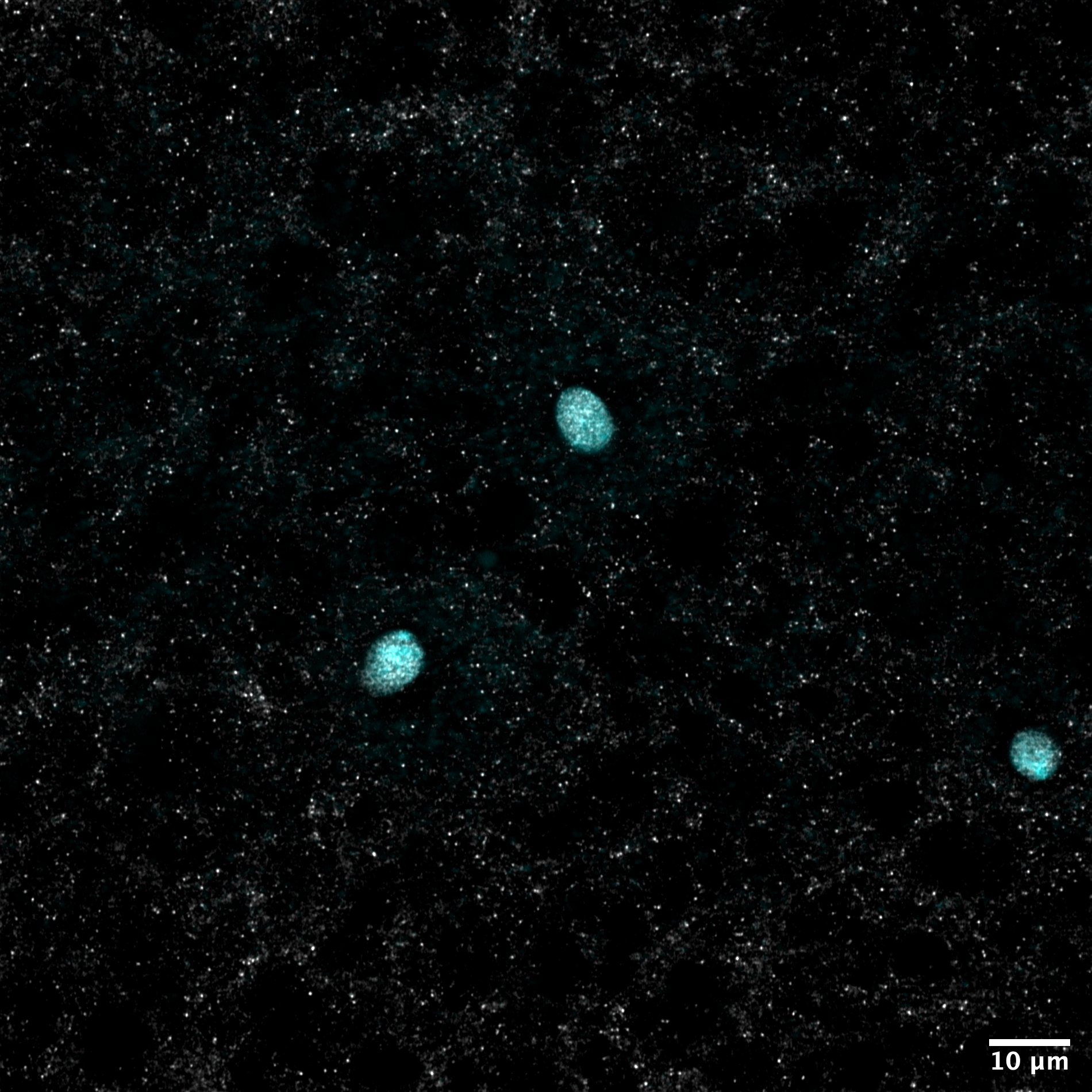

Supplement: Supplementary file 8 — Source data Fig. 1 [file 44318_2024_127_MOESM8_ESM.zip › figure1/figure1h/G9a/figure 1h_G9a_cycle 4-5_G9a_DAPI.jpg]

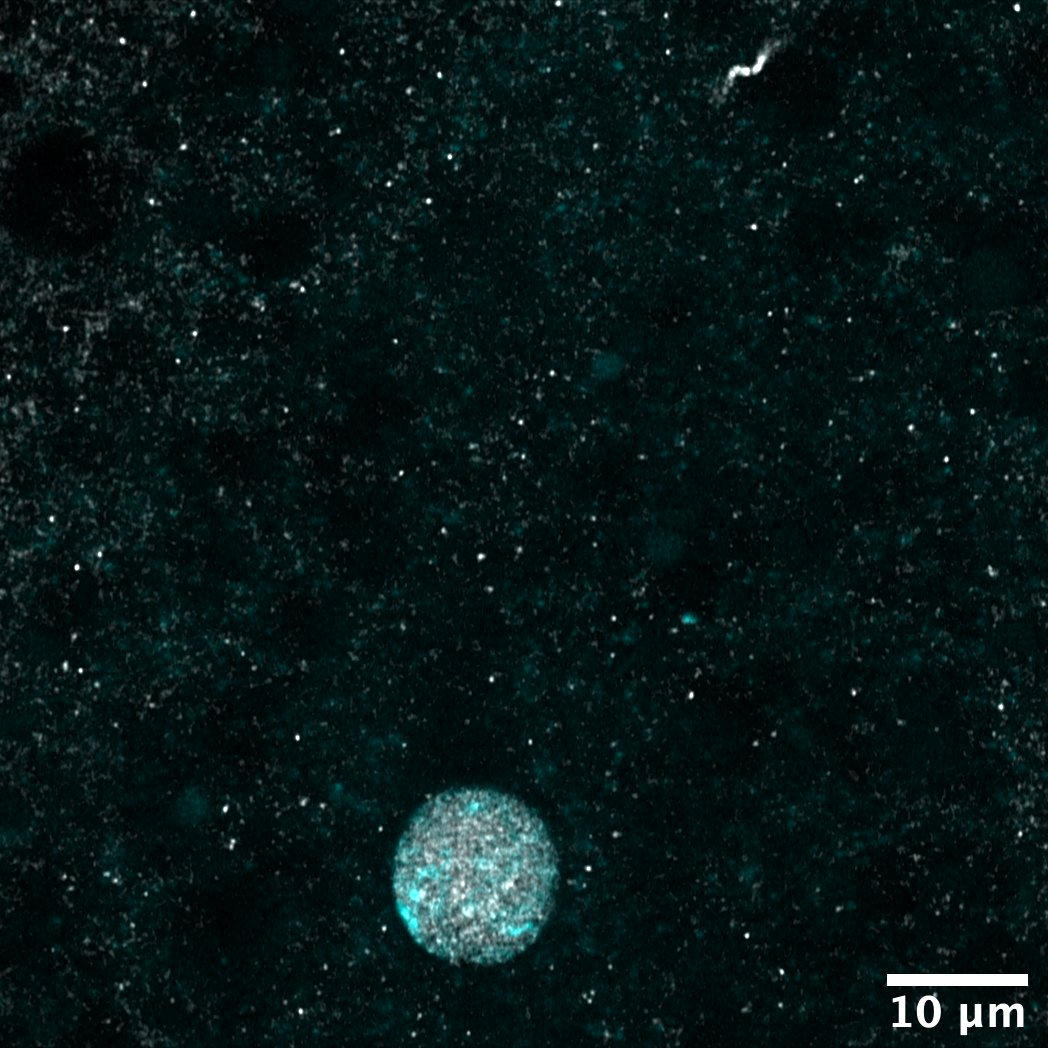

Supplement: Supplementary file 8 — Source data Fig. 1 [file 44318_2024_127_MOESM8_ESM.zip › figure1/figure1h/G9a/figure 1h_G9a_cycle 1_G9a_DAPI.jpg]

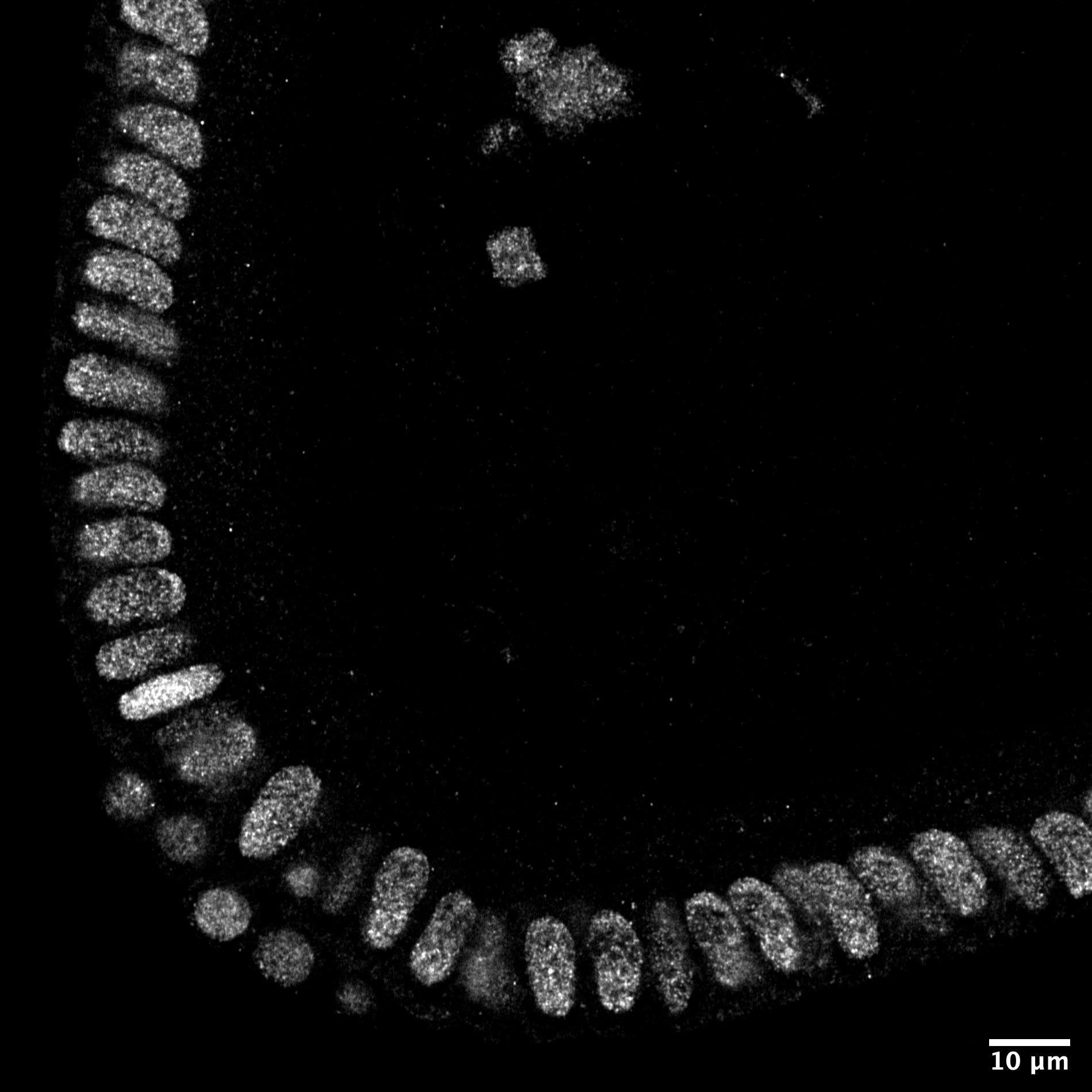

Supplement: Supplementary file 8 — Source data Fig. 1 [file 44318_2024_127_MOESM8_ESM.zip › figure1/figure1h/G9a/figure 1h_G9a_cycle 14_G9a.jpg]

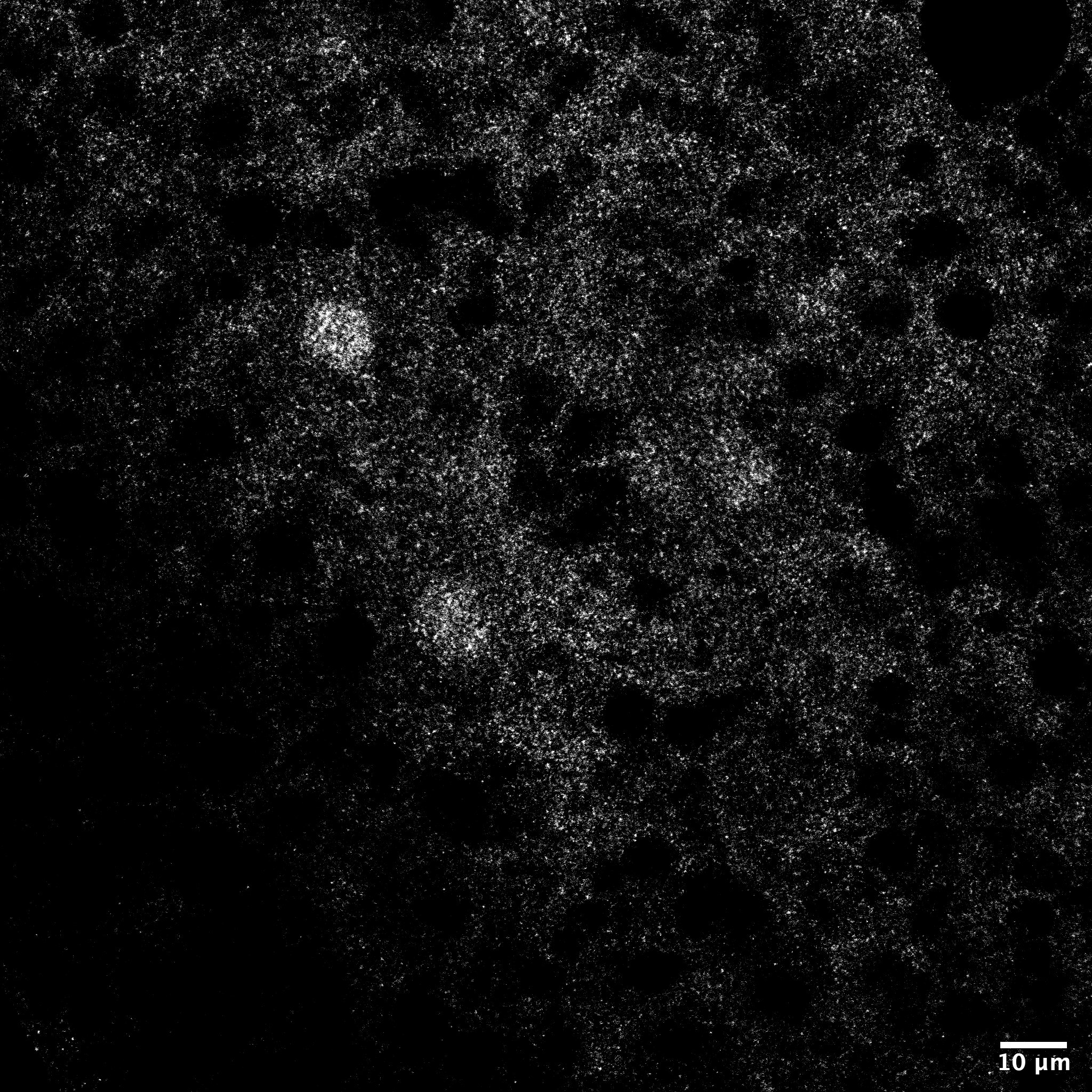

Supplement: Supplementary file 8 — Source data Fig. 1 [file 44318_2024_127_MOESM8_ESM.zip › figure1/figure1h/GFP_egg_embryo/figure 1h_GFP-dSetDB1_cycle 5_GFP.jpg]

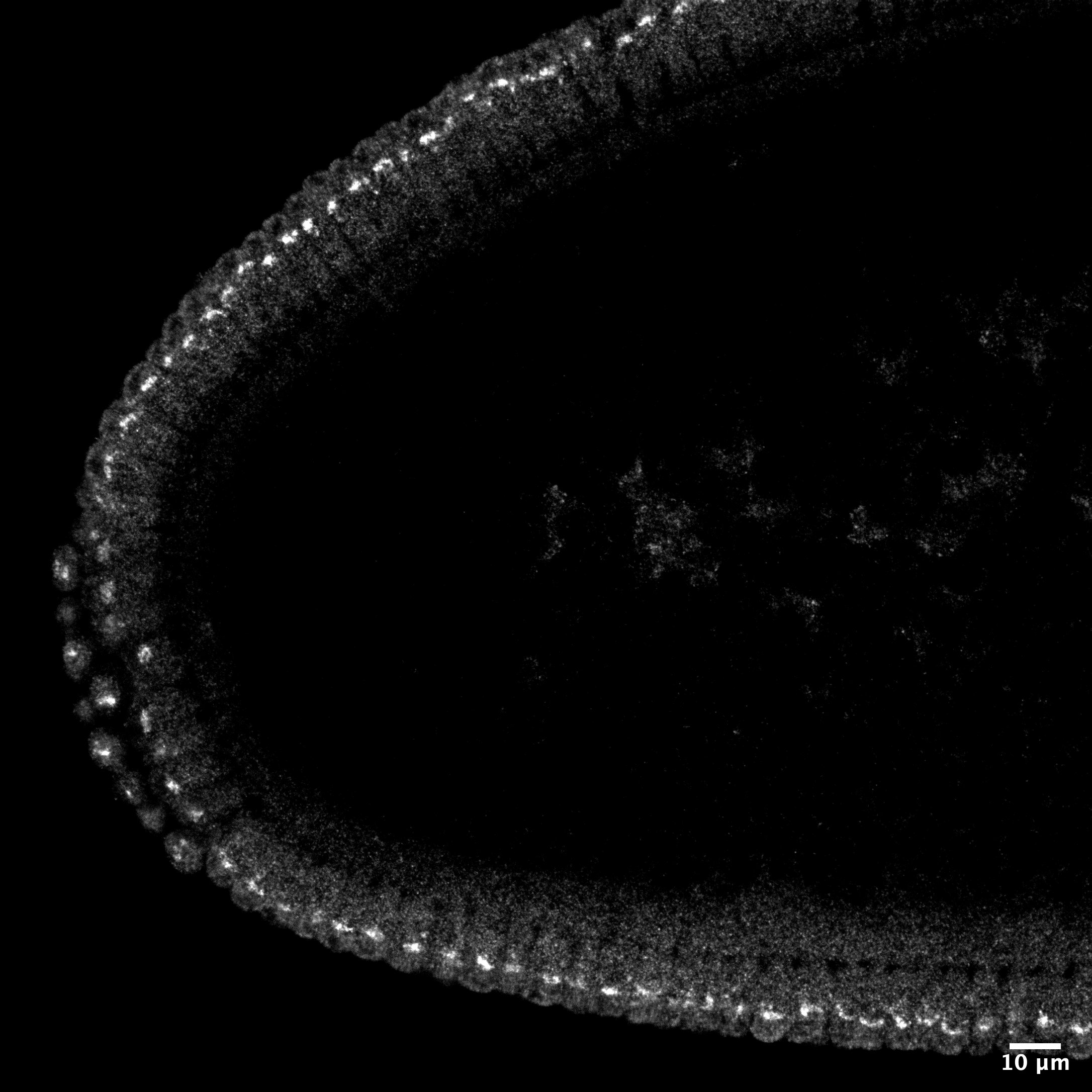

Supplement: Supplementary file 8 — Source data Fig. 1 [file 44318_2024_127_MOESM8_ESM.zip › figure1/figure1h/GFP_egg_embryo/figure 1h_GFP-dSetDB1_cycle 14_GFP.jpg]

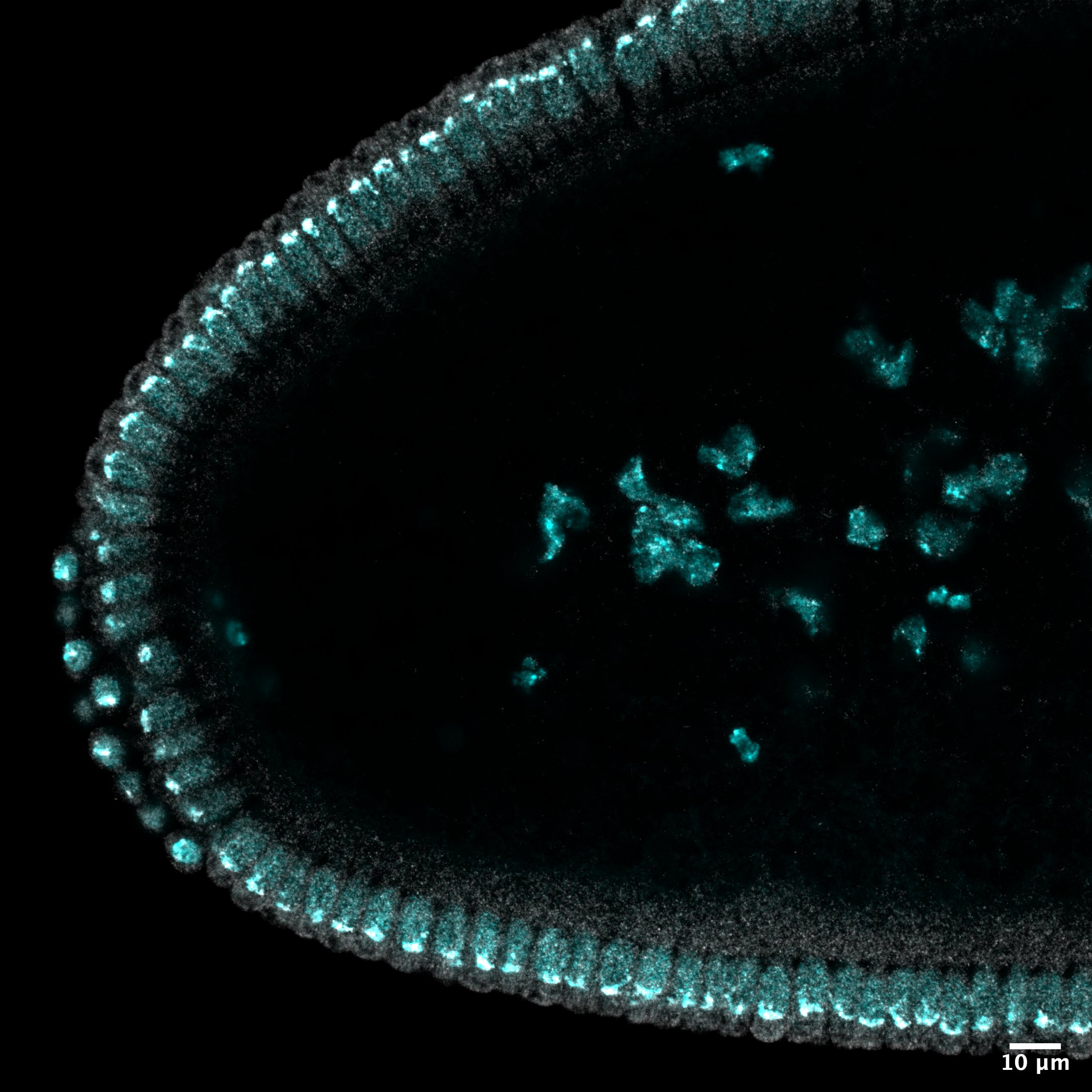

Supplement: Supplementary file 8 — Source data Fig. 1 [file 44318_2024_127_MOESM8_ESM.zip › figure1/figure1h/GFP_egg_embryo/figure 1h_GFP-dSetDB1_cycle 14_DAPI-GFP.jpg]

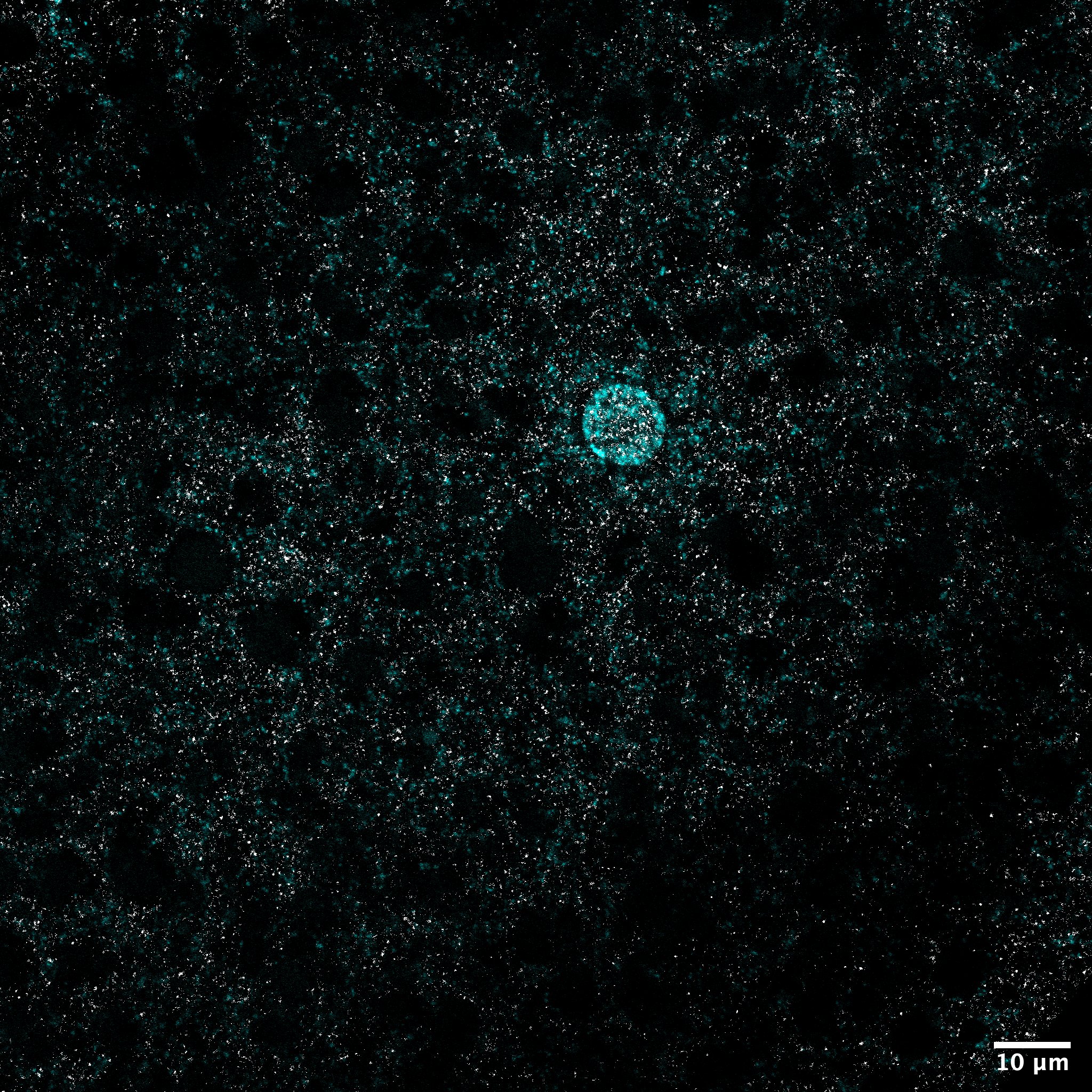

Supplement: Supplementary file 8 — Source data Fig. 1 [file 44318_2024_127_MOESM8_ESM.zip › figure1/figure1h/GFP_egg_embryo/figure 1h_GFP-dSetDB1_cycle 2_DAPI-GFP.jpg]

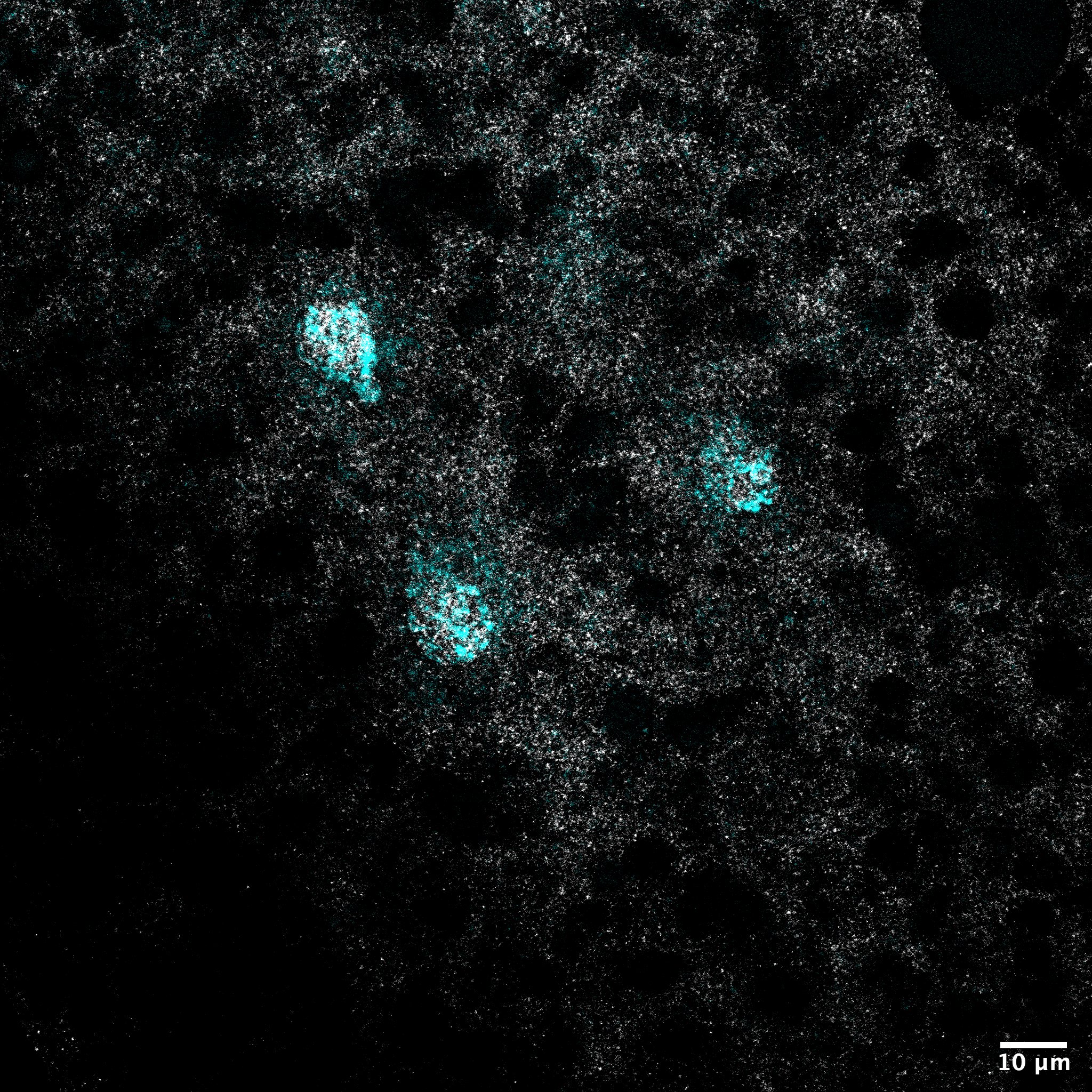

Supplement: Supplementary file 8 — Source data Fig. 1 [file 44318_2024_127_MOESM8_ESM.zip › figure1/figure1h/GFP_egg_embryo/figure 1h_GFP-dSetDB1_cycle 5_DAPI-GFP.jpg]

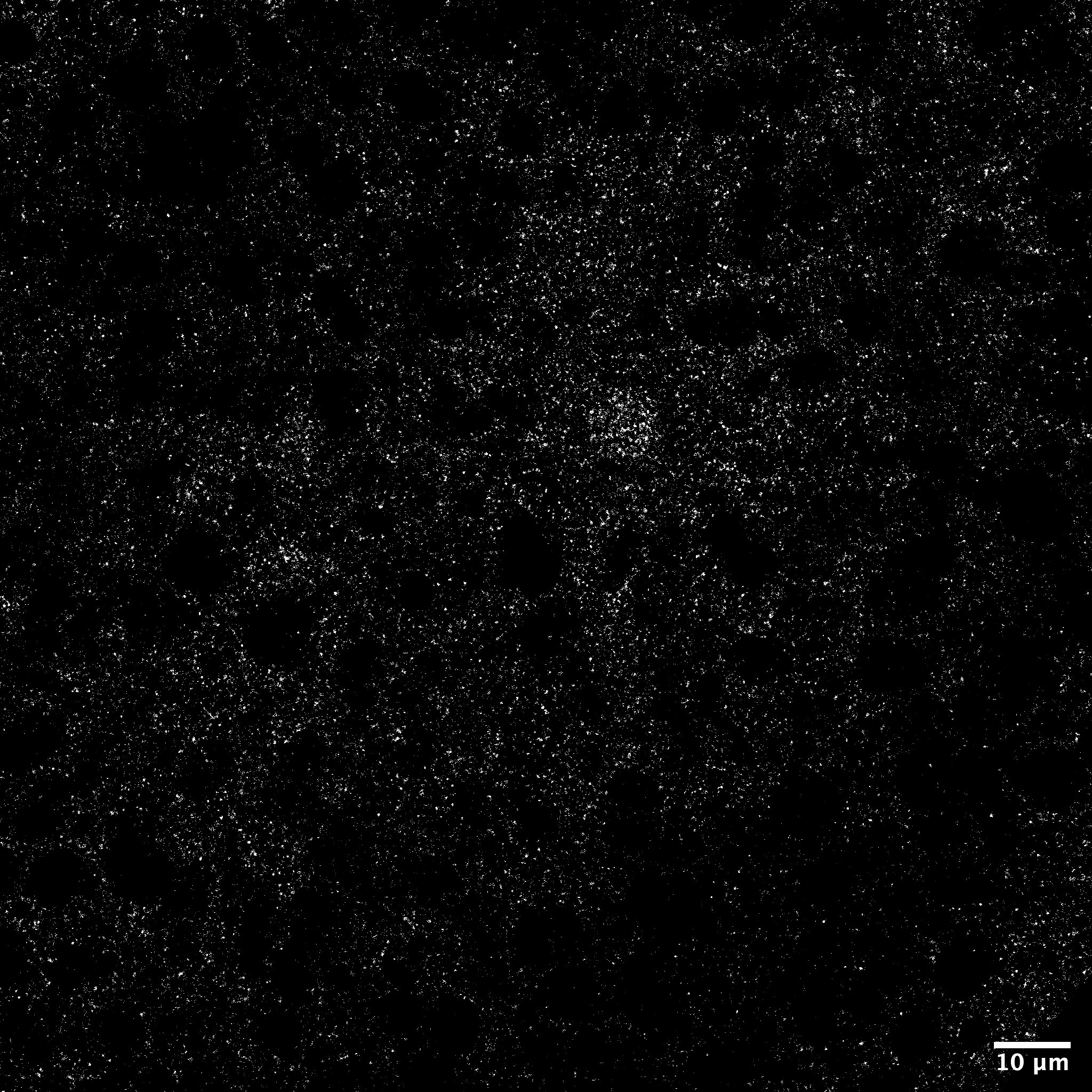

Supplement: Supplementary file 8 — Source data Fig. 1 [file 44318_2024_127_MOESM8_ESM.zip › figure1/figure1h/GFP_egg_embryo/figure 1h_GFP-dSetDB1_cycle 2_GFP.jpg]

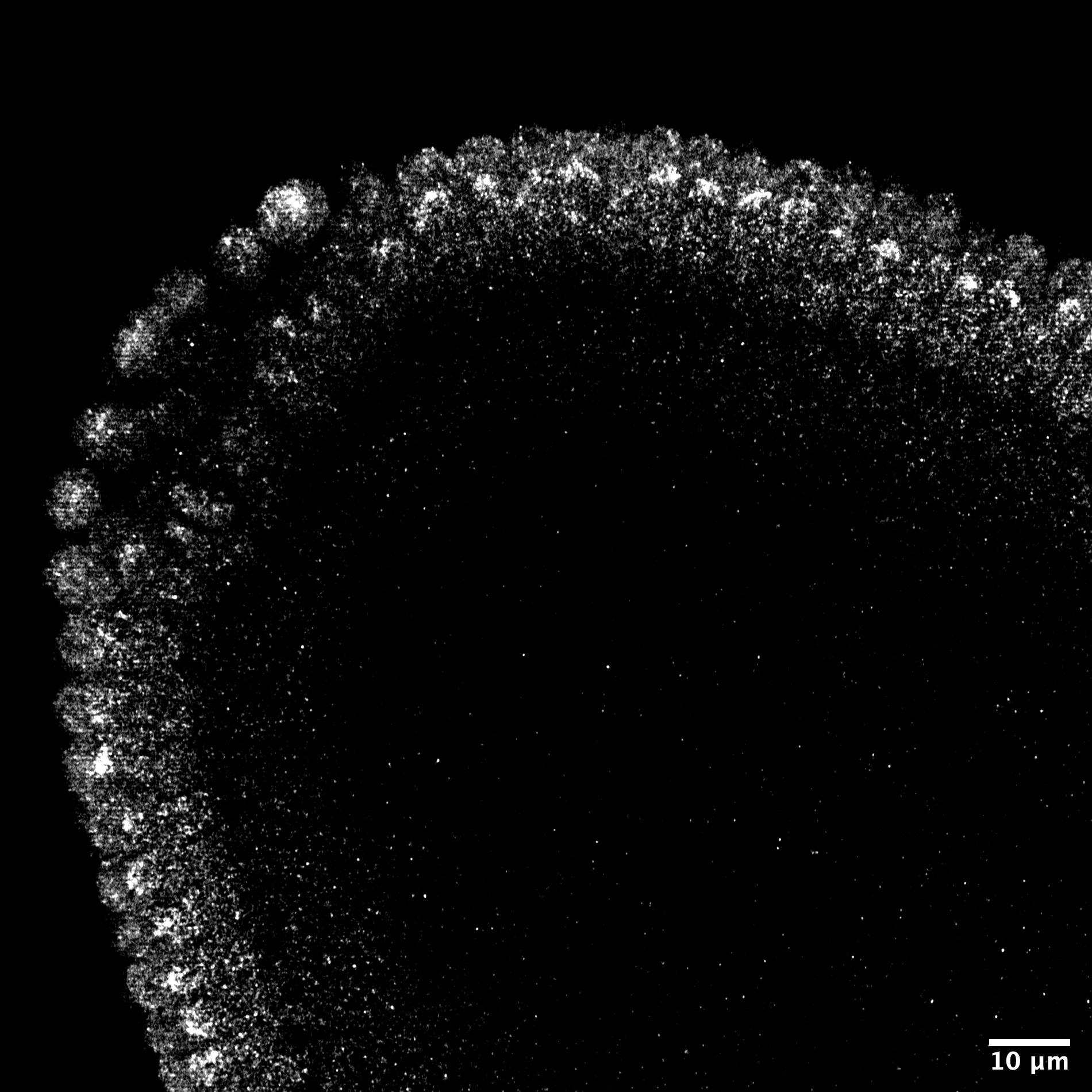

Supplement: Supplementary file 8 — Source data Fig. 1 [file 44318_2024_127_MOESM8_ESM.zip › figure1/figure1h/Su(var)3-9_GFP/figure 1h_Su(var)3-9-GFP_cycle 14_GFP.jpg]

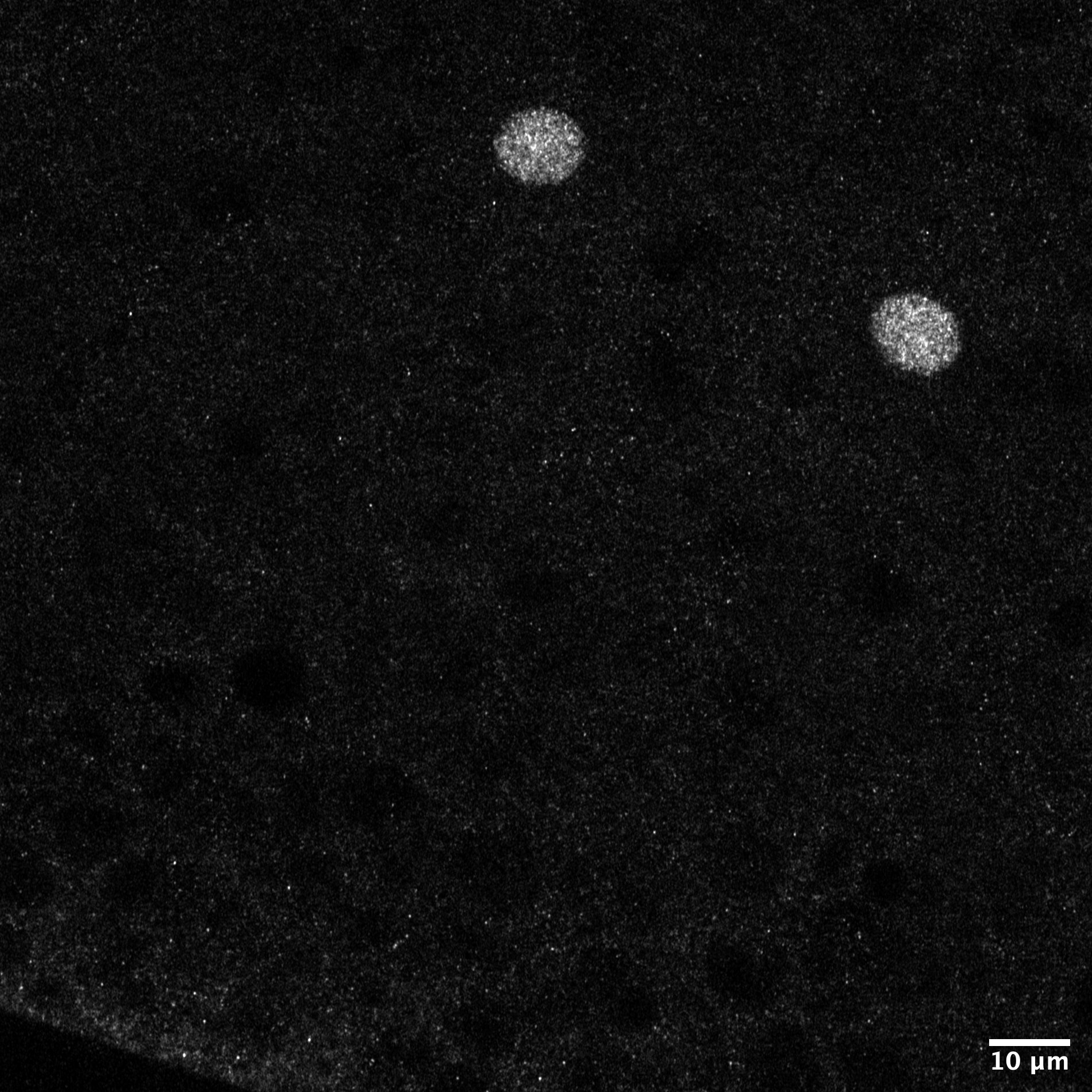

Supplement: Supplementary file 8 — Source data Fig. 1 [file 44318_2024_127_MOESM8_ESM.zip › figure1/figure1h/Su(var)3-9_GFP/figure 1h_Su(var)3-9-GFP_cycle 2_GFP.jpg]

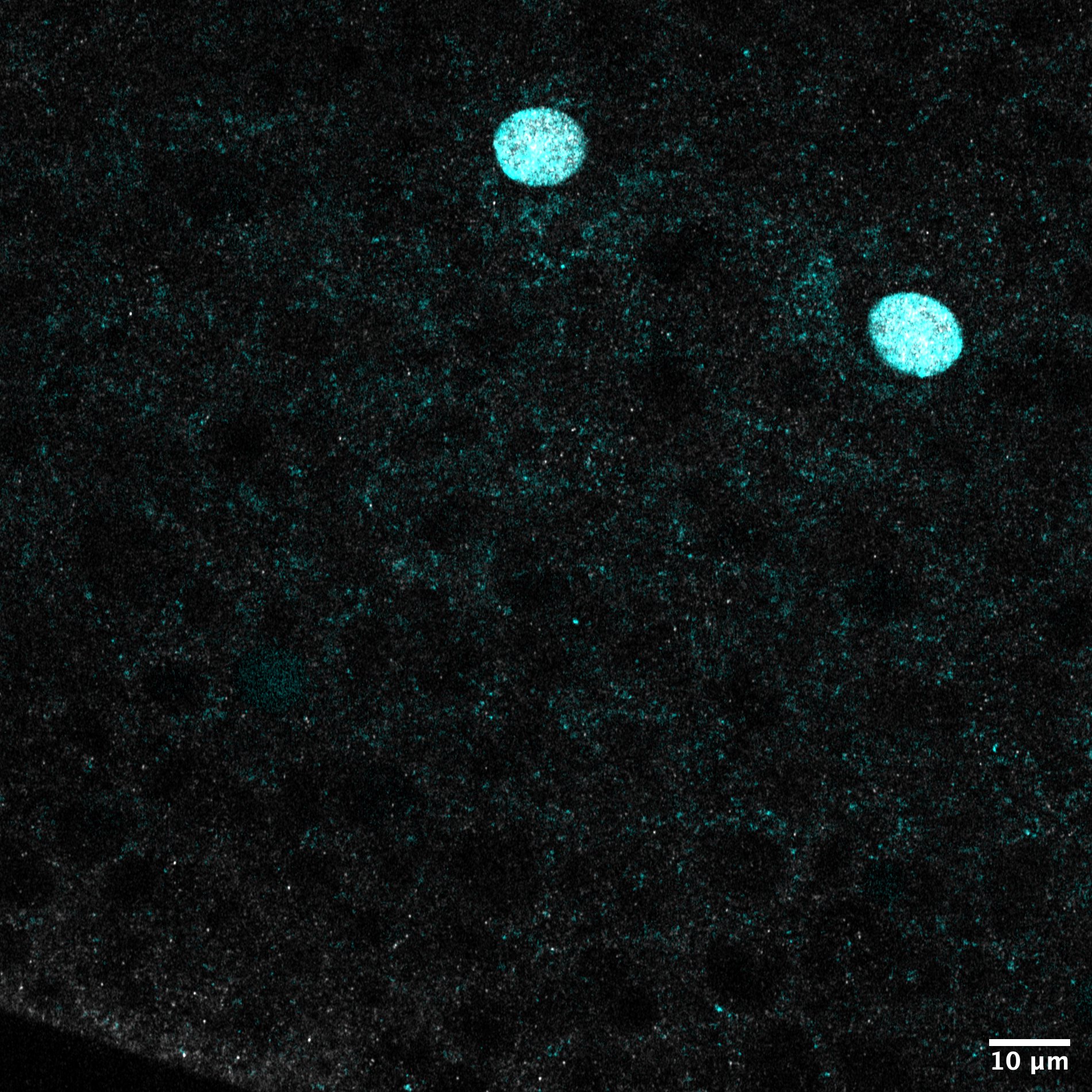

Supplement: Supplementary file 8 — Source data Fig. 1 [file 44318_2024_127_MOESM8_ESM.zip › figure1/figure1h/Su(var)3-9_GFP/figure 1h_Su(var)3-9-GFP_cycle 2_GFP_DAPI.jpg]

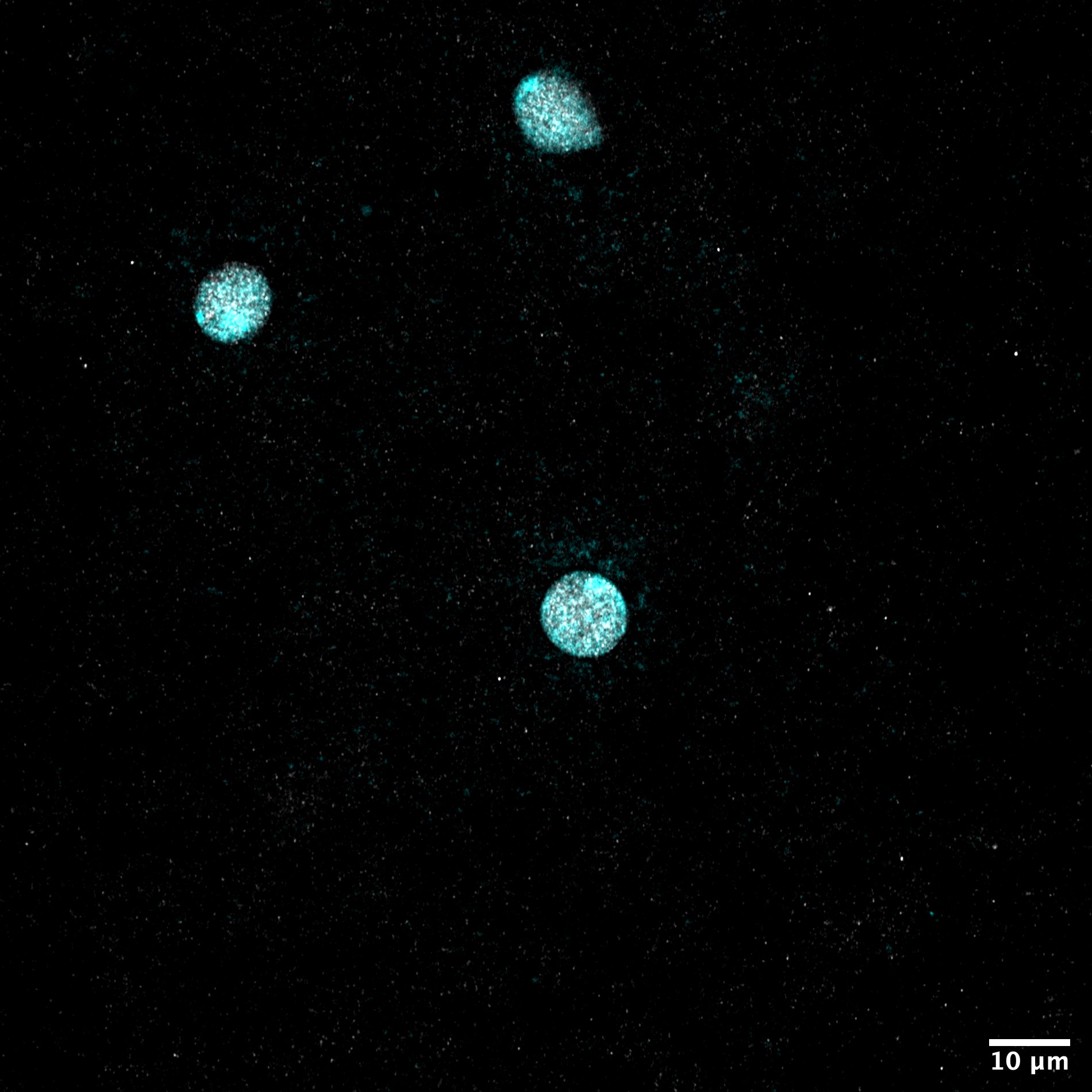

Supplement: Supplementary file 8 — Source data Fig. 1 [file 44318_2024_127_MOESM8_ESM.zip › figure1/figure1h/Su(var)3-9_GFP/figure 1h_Su(var)3-9-GFP_cycle 4-5_GFP_DAPI.jpg]

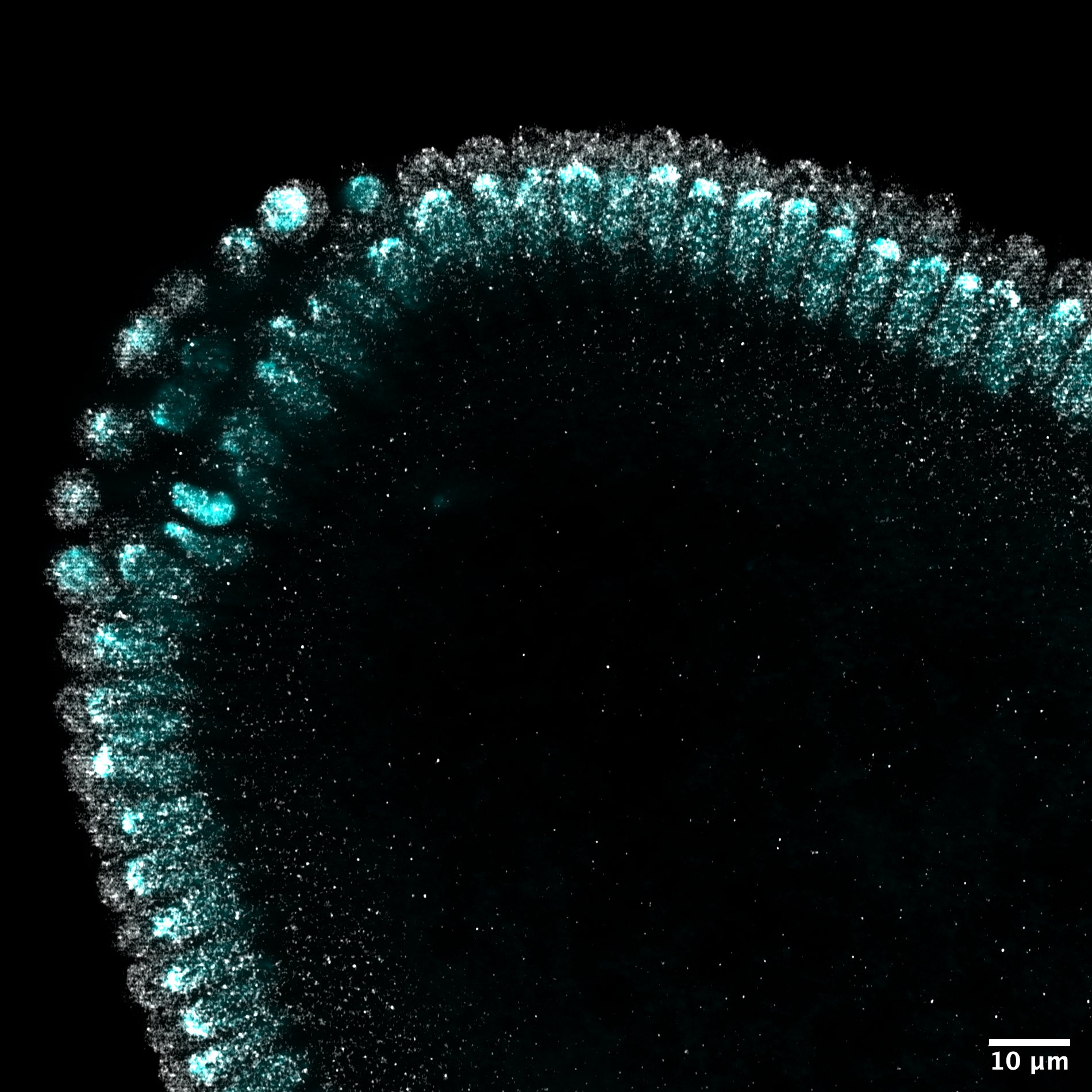

Supplement: Supplementary file 8 — Source data Fig. 1 [file 44318_2024_127_MOESM8_ESM.zip › figure1/figure1h/Su(var)3-9_GFP/figure 1h_Su(var)3-9-GFP_cycle 14_GFP_DAPI.jpg]

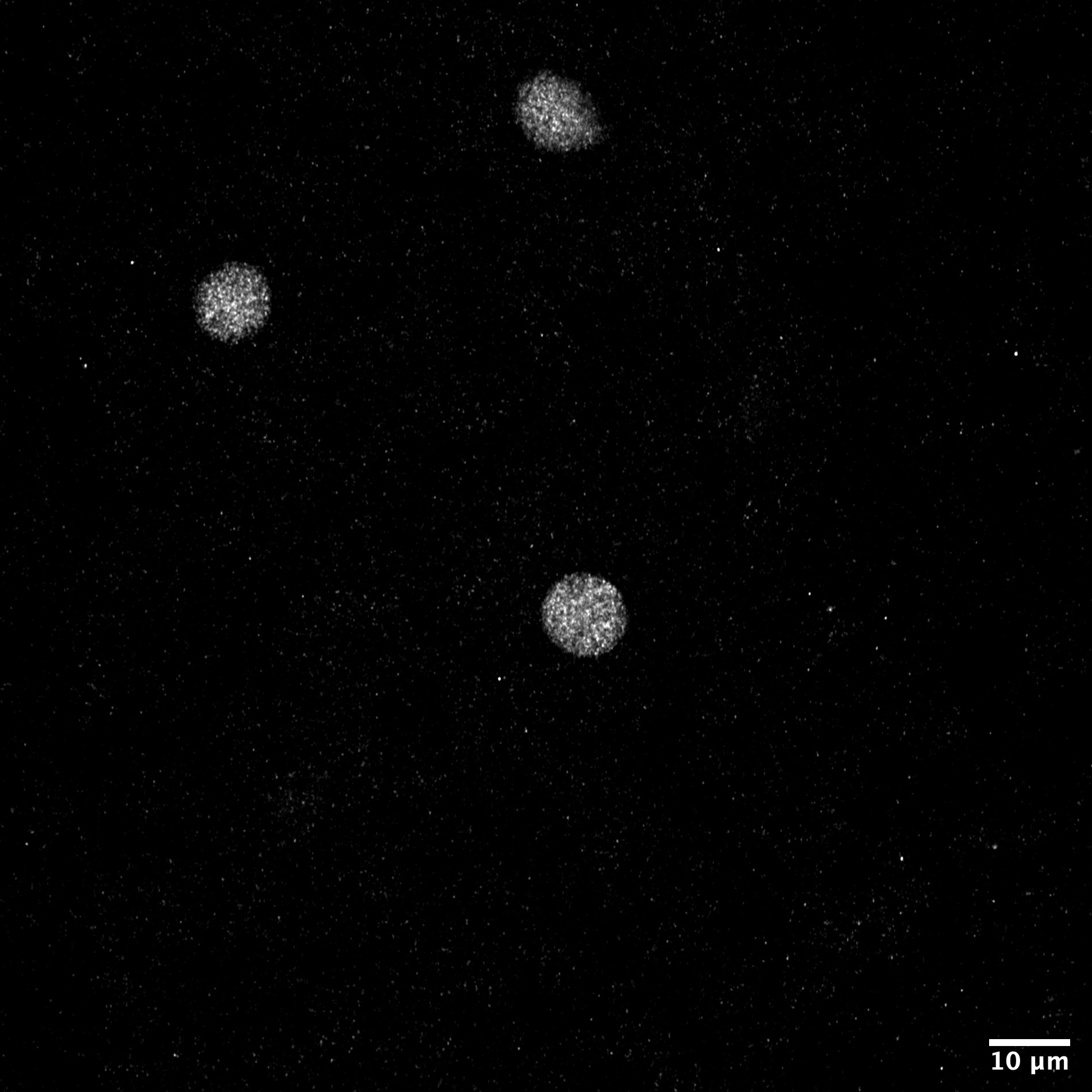

Supplement: Supplementary file 8 — Source data Fig. 1 [file 44318_2024_127_MOESM8_ESM.zip › figure1/figure1h/Su(var)3-9_GFP/figure 1h_Su(var)3-9-GFP_cycle 4-5_GFP.jpg]

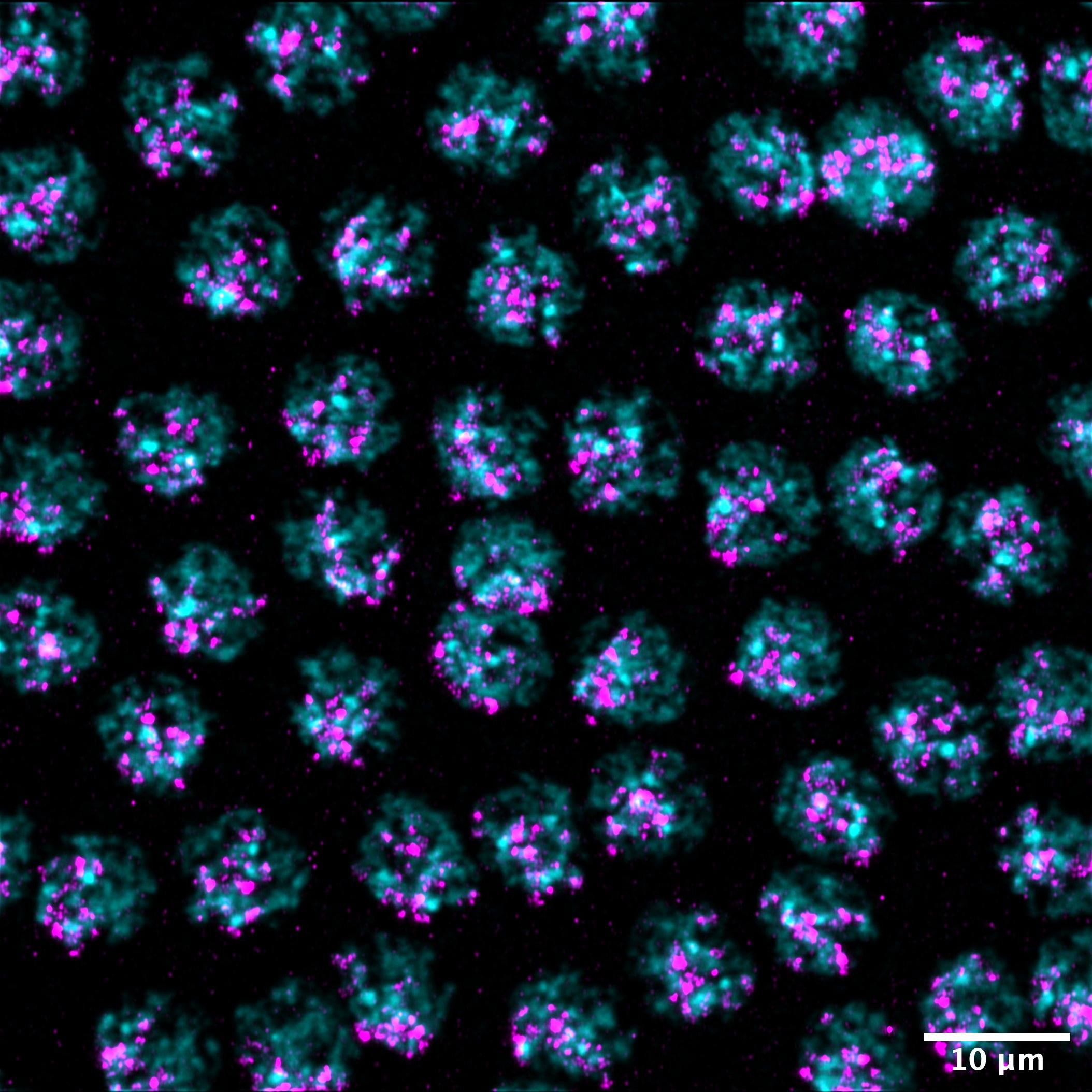

Supplement: Supplementary file 9 — Source data Fig. 2 [file 44318_2024_127_MOESM9_ESM.zip › figure2/figure2c/figure 2c_ctr_H3k9me3_DAPI_cycle10-11.jpg]

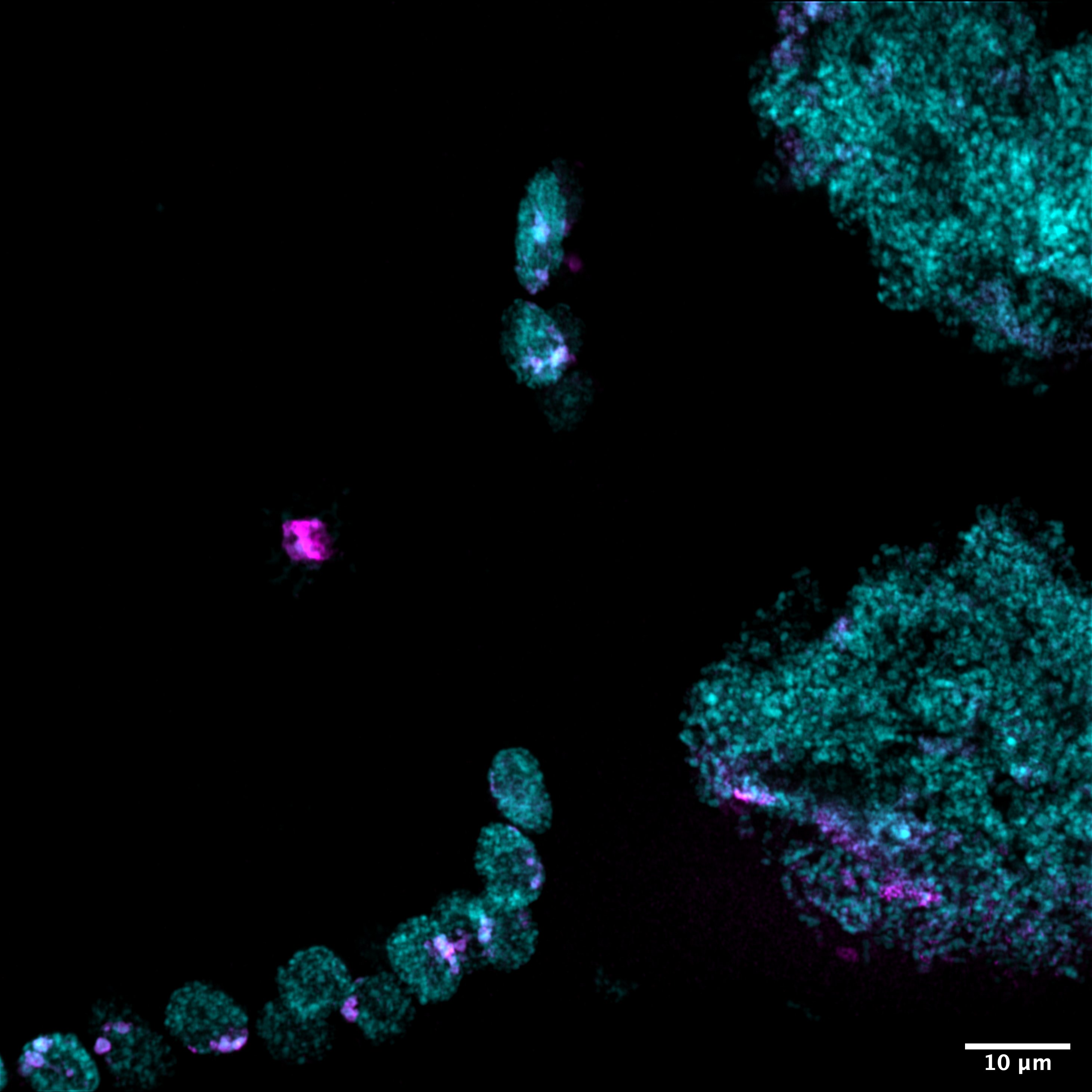

Supplement: Supplementary file 9 — Source data Fig. 2 [file 44318_2024_127_MOESM9_ESM.zip › figure2/figure2c/figure 2c_ctr_H3K9me3_DAPI_stage10A_ovary.jpg]

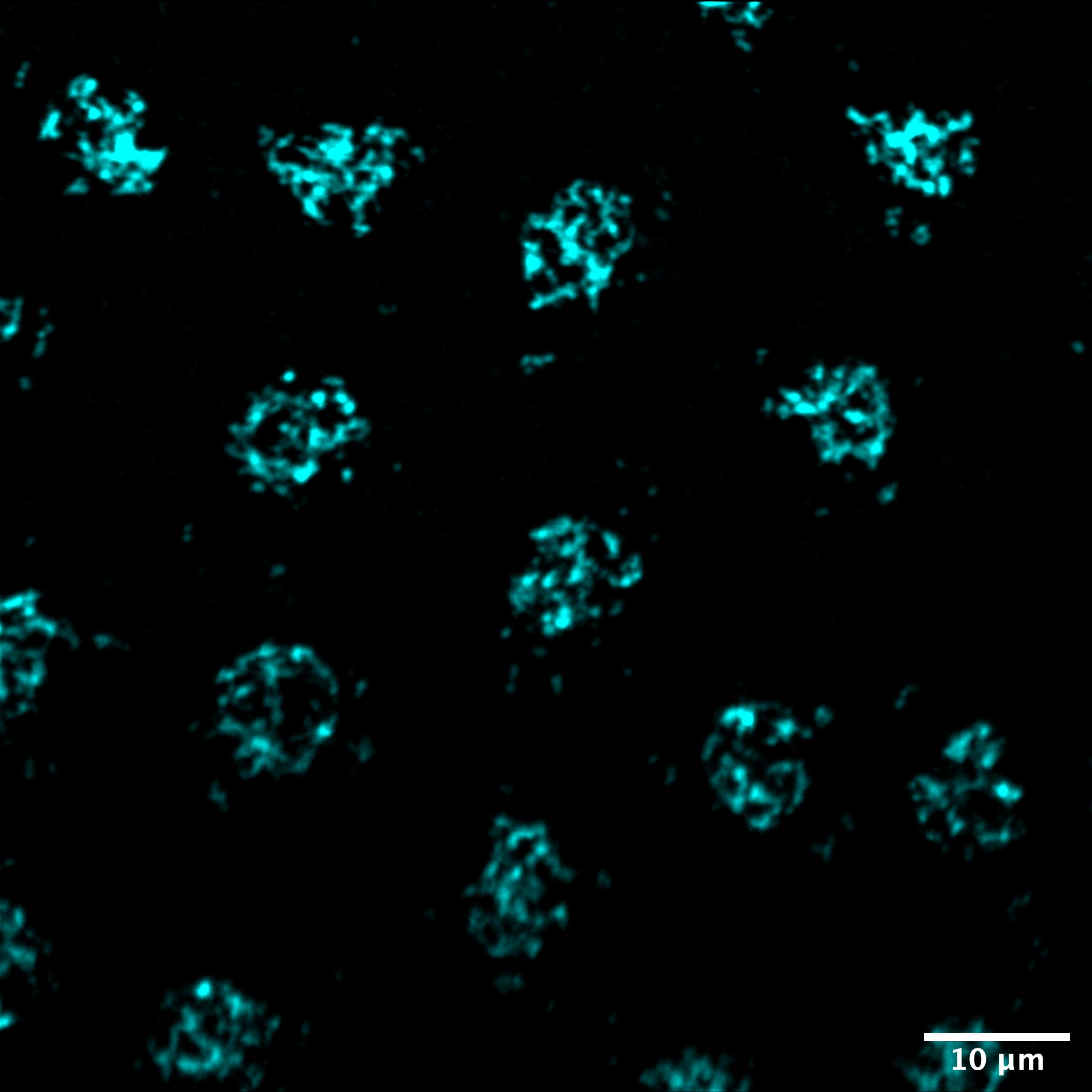

Supplement: Supplementary file 9 — Source data Fig. 2 [file 44318_2024_127_MOESM9_ESM.zip › figure2/figure2c/figure 2c_TM_H3k9me3_DAPI_cycle10.jpg]

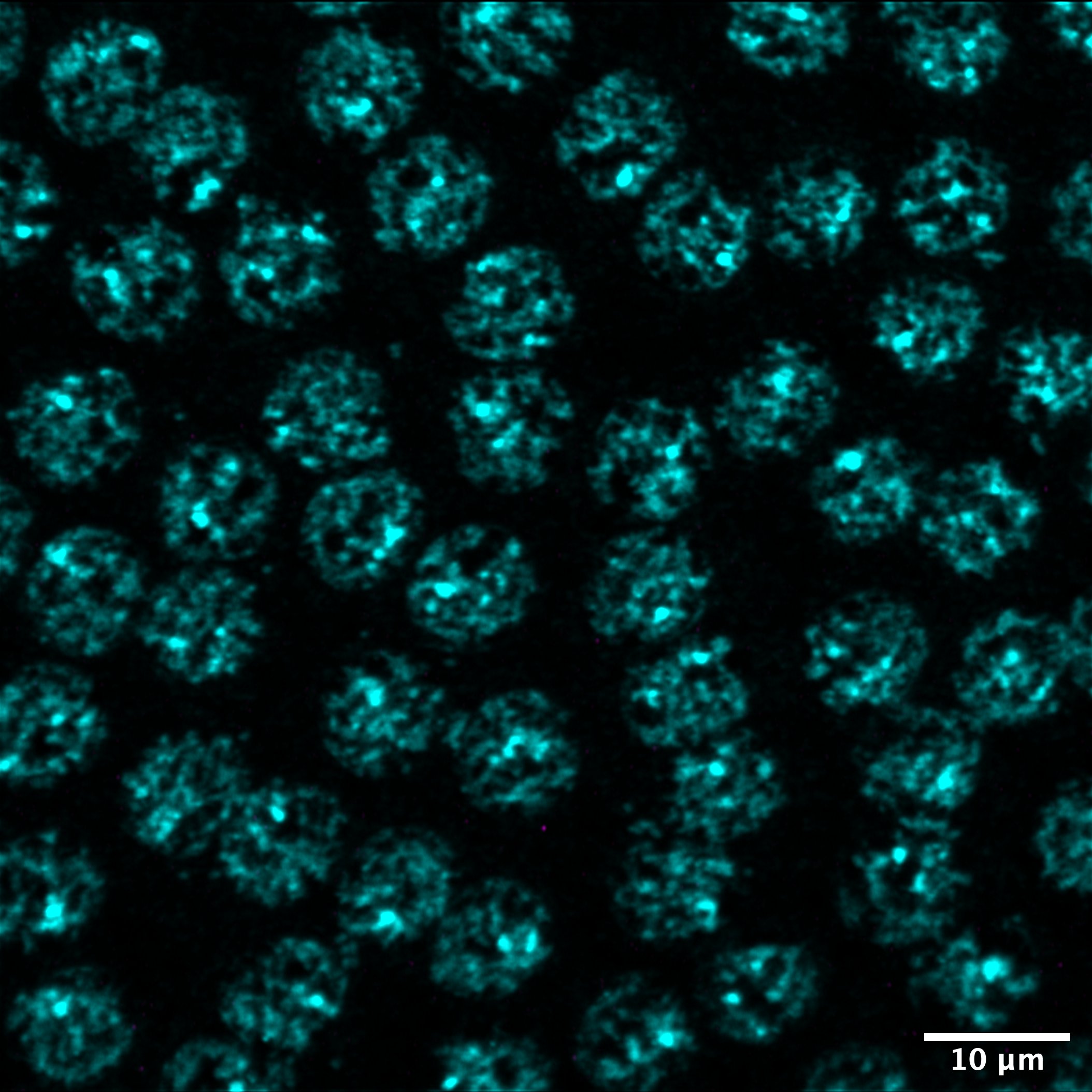

Supplement: Supplementary file 9 — Source data Fig. 2 [file 44318_2024_127_MOESM9_ESM.zip › figure2/figure2c/figure 2c_TM_H3k9me3_DAPI_cycle11-12.jpg]

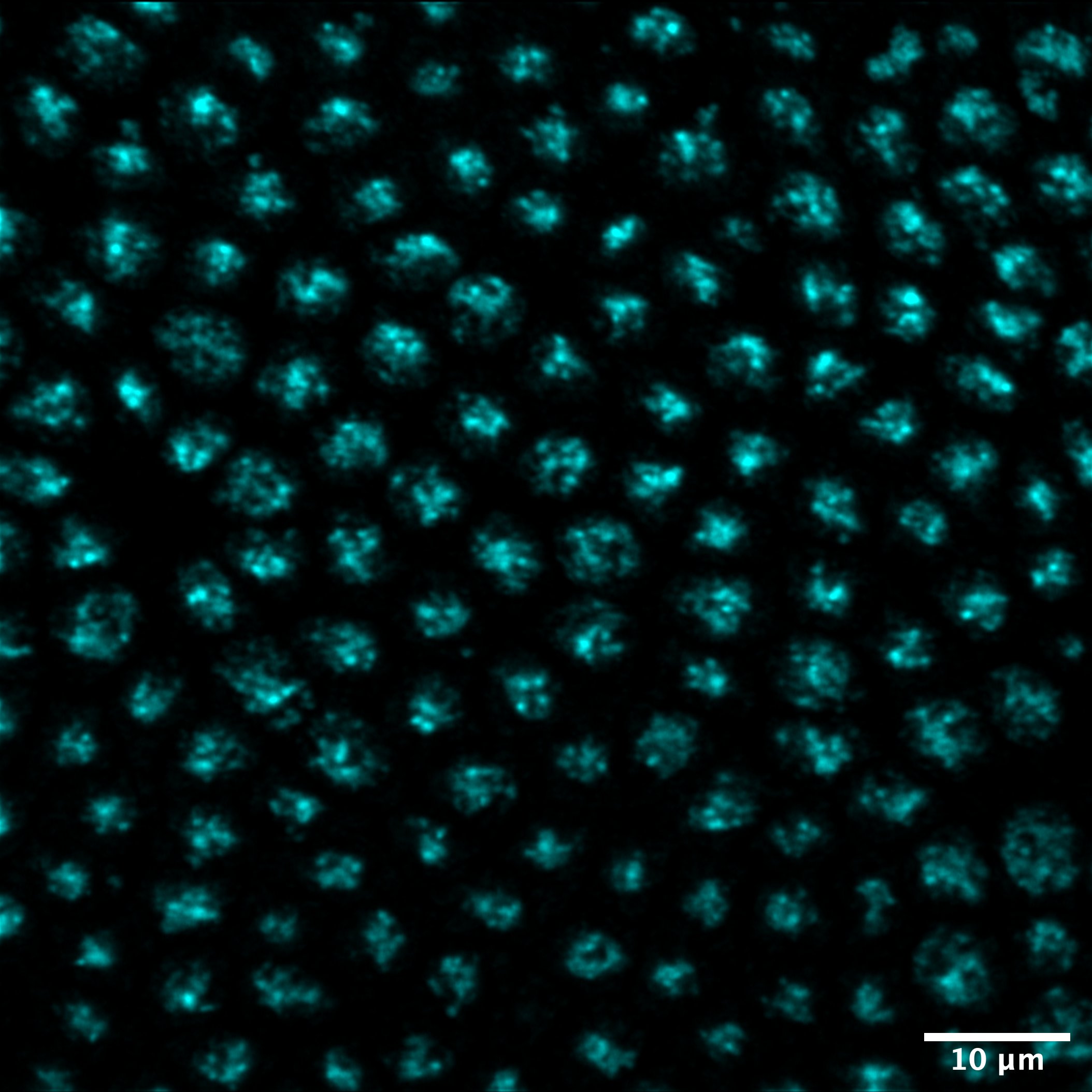

Supplement: Supplementary file 9 — Source data Fig. 2 [file 44318_2024_127_MOESM9_ESM.zip › figure2/figure2c/figure 2c_TM_H3k9me3_DAPI_cycle14.jpg]

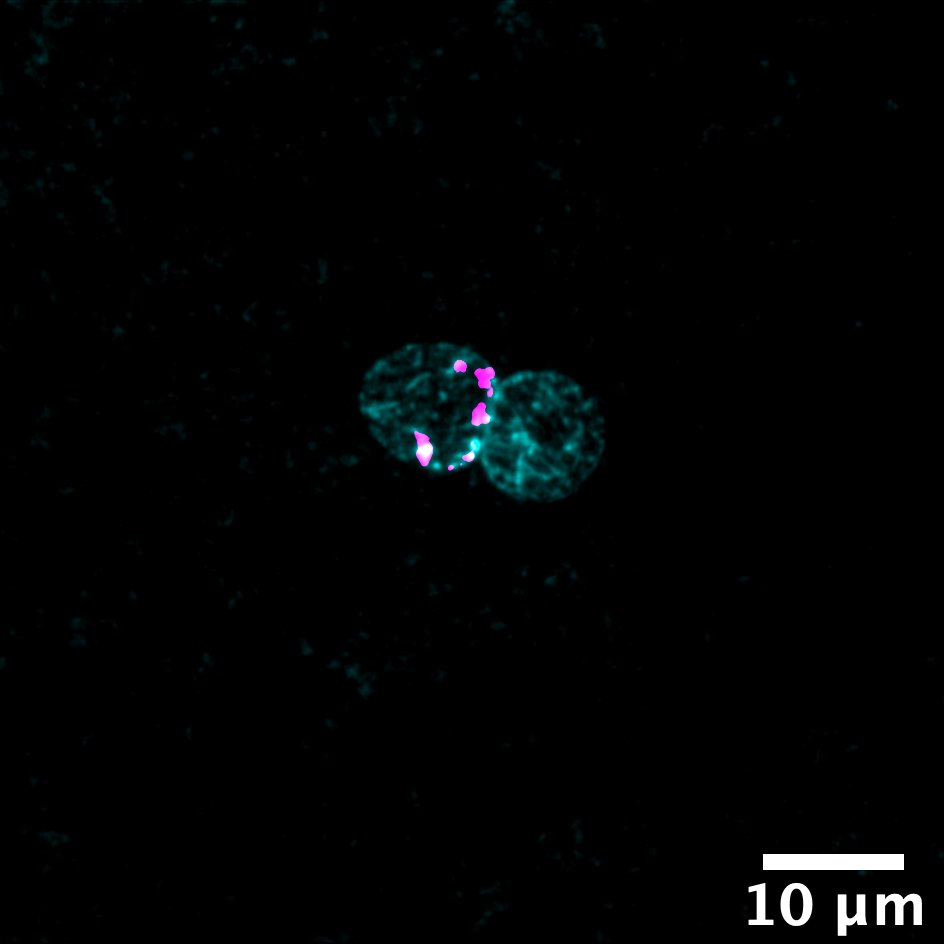

Supplement: Supplementary file 9 — Source data Fig. 2 [file 44318_2024_127_MOESM9_ESM.zip › figure2/figure2c/figure 2c_ctr_H3K9me3_DAPI_apposition.jpg]

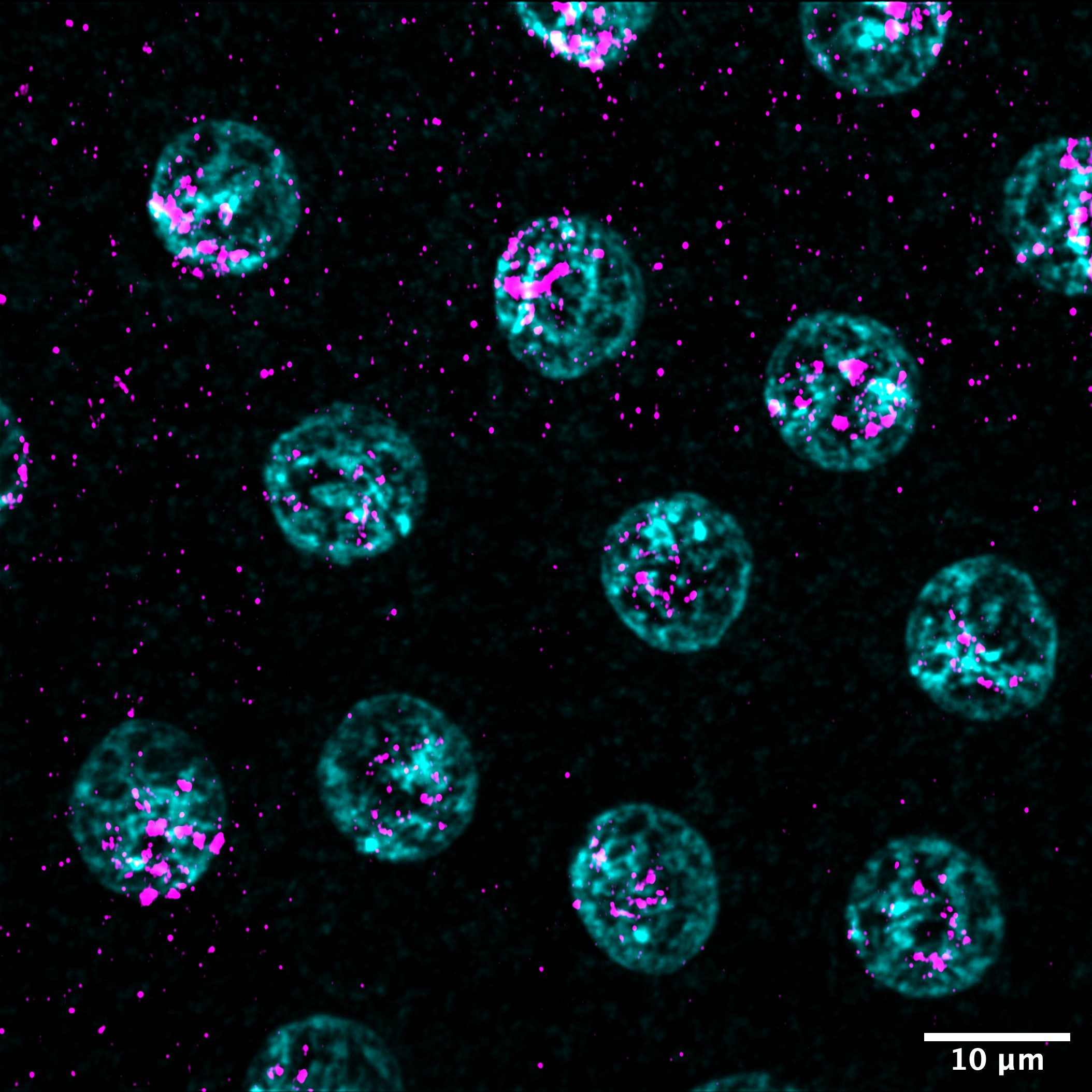

Supplement: Supplementary file 9 — Source data Fig. 2 [file 44318_2024_127_MOESM9_ESM.zip › figure2/figure2c/figure 2c_ctr_H3k9me3_DAPI_cycle10.jpg]

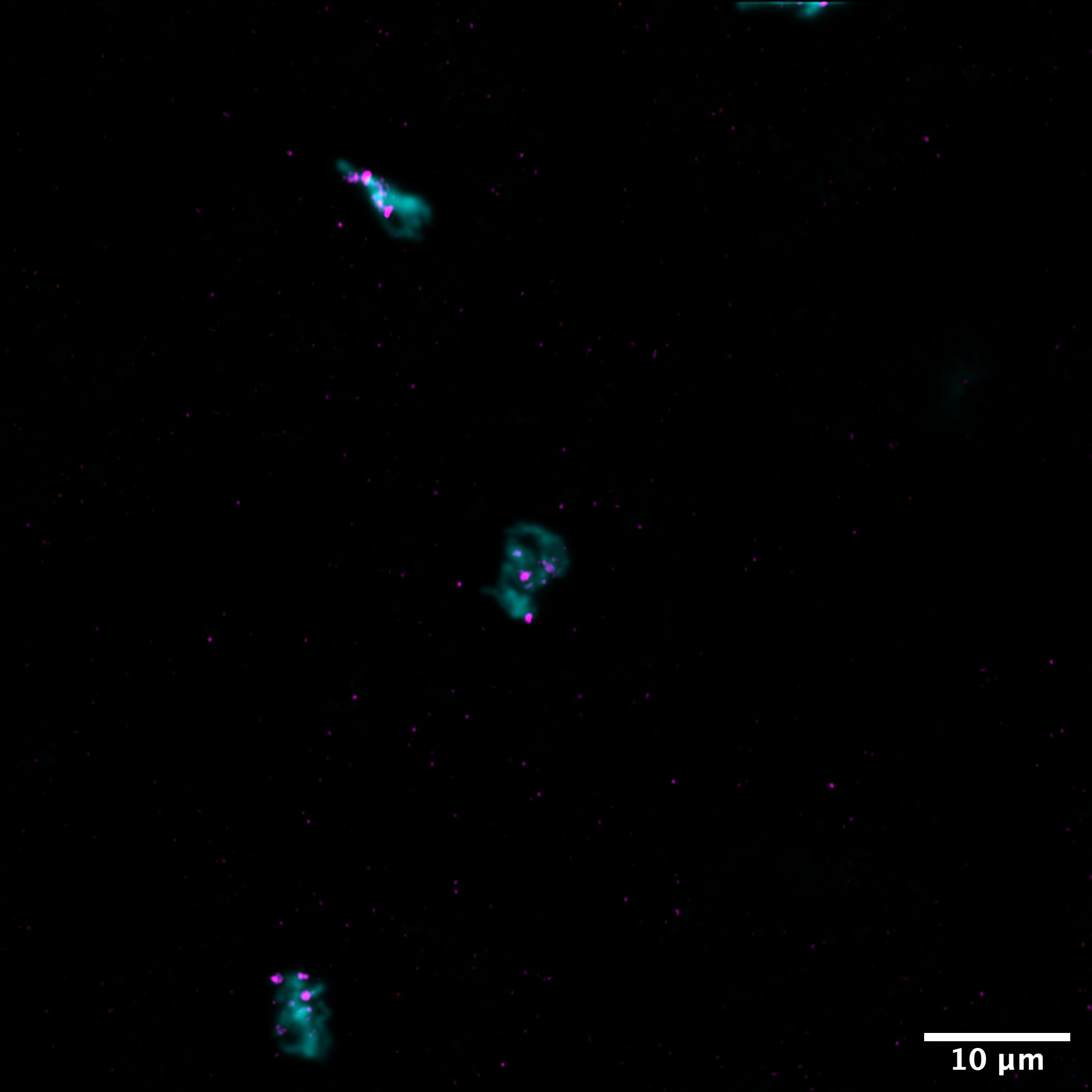

Supplement: Supplementary file 9 — Source data Fig. 2 [file 44318_2024_127_MOESM9_ESM.zip › figure2/figure2c/figure 2c_ctr_H3K9me3_DAPI_cycle7.jpg]

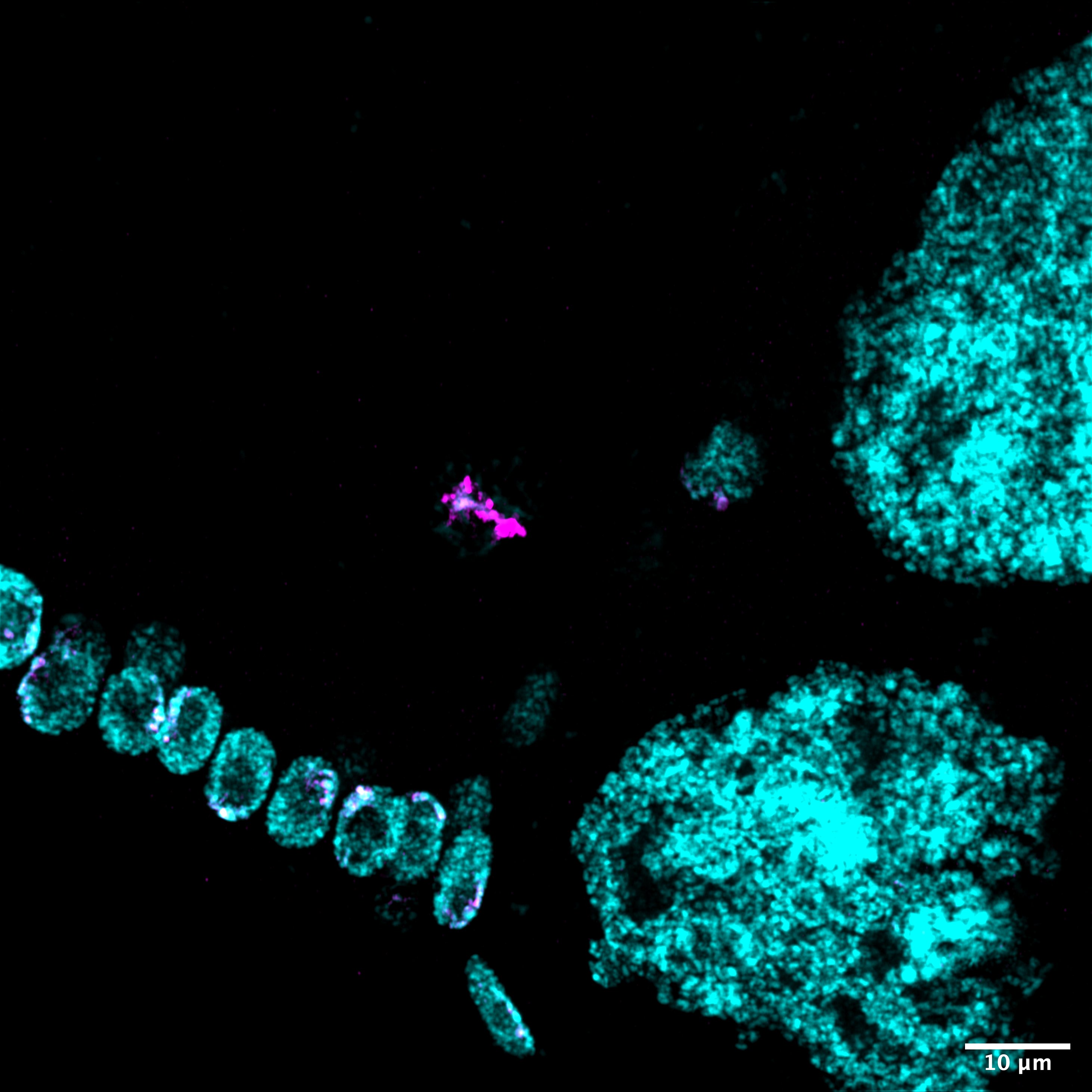

Supplement: Supplementary file 9 — Source data Fig. 2 [file 44318_2024_127_MOESM9_ESM.zip › figure2/figure2c/figure 2c_TM_H3K9me3_DAPI_stage10_ovary.jpg]

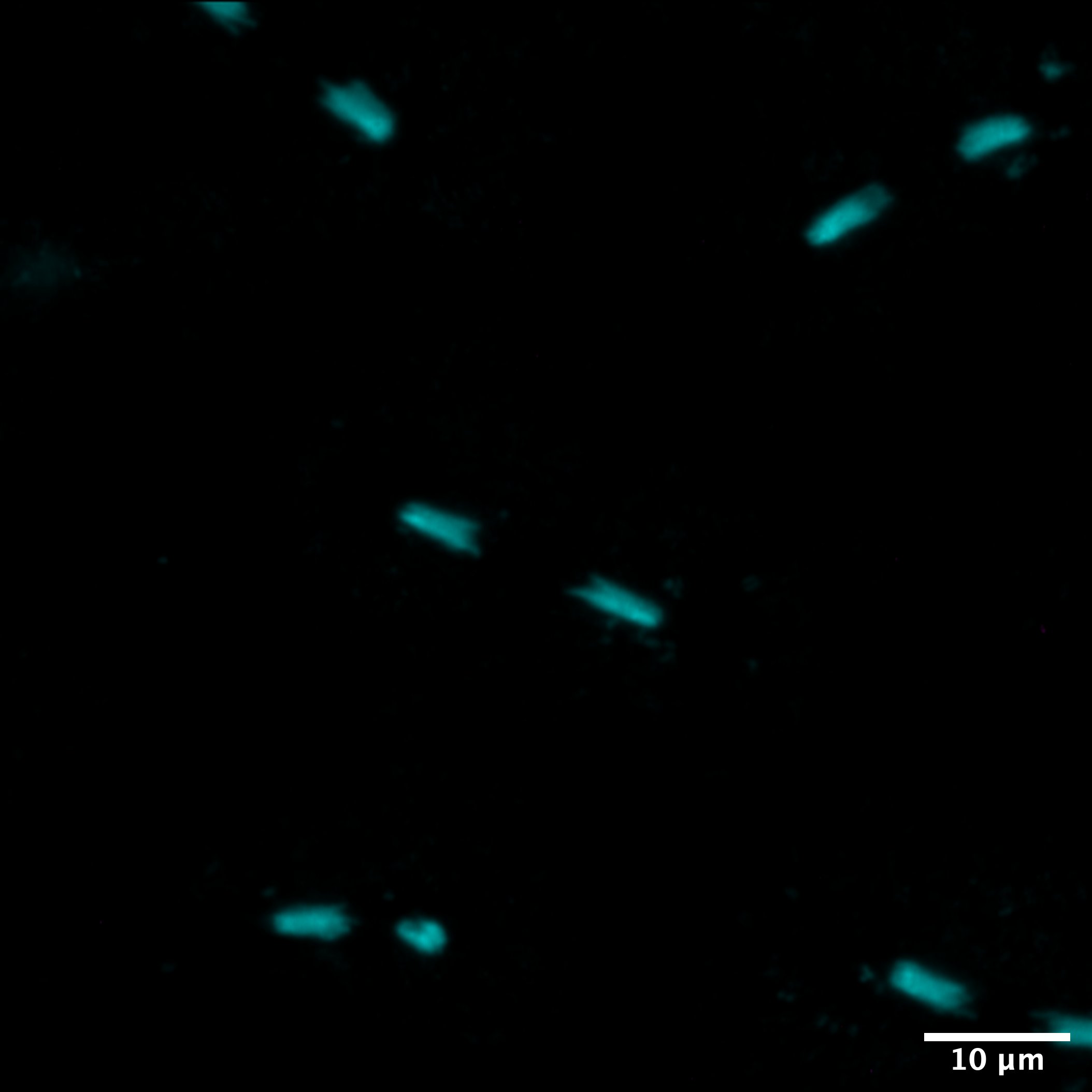

Supplement: Supplementary file 9 — Source data Fig. 2 [file 44318_2024_127_MOESM9_ESM.zip › figure2/figure2c/figure 2c_TM_H3K9me3_DAPI_cycle6-7.jpg]

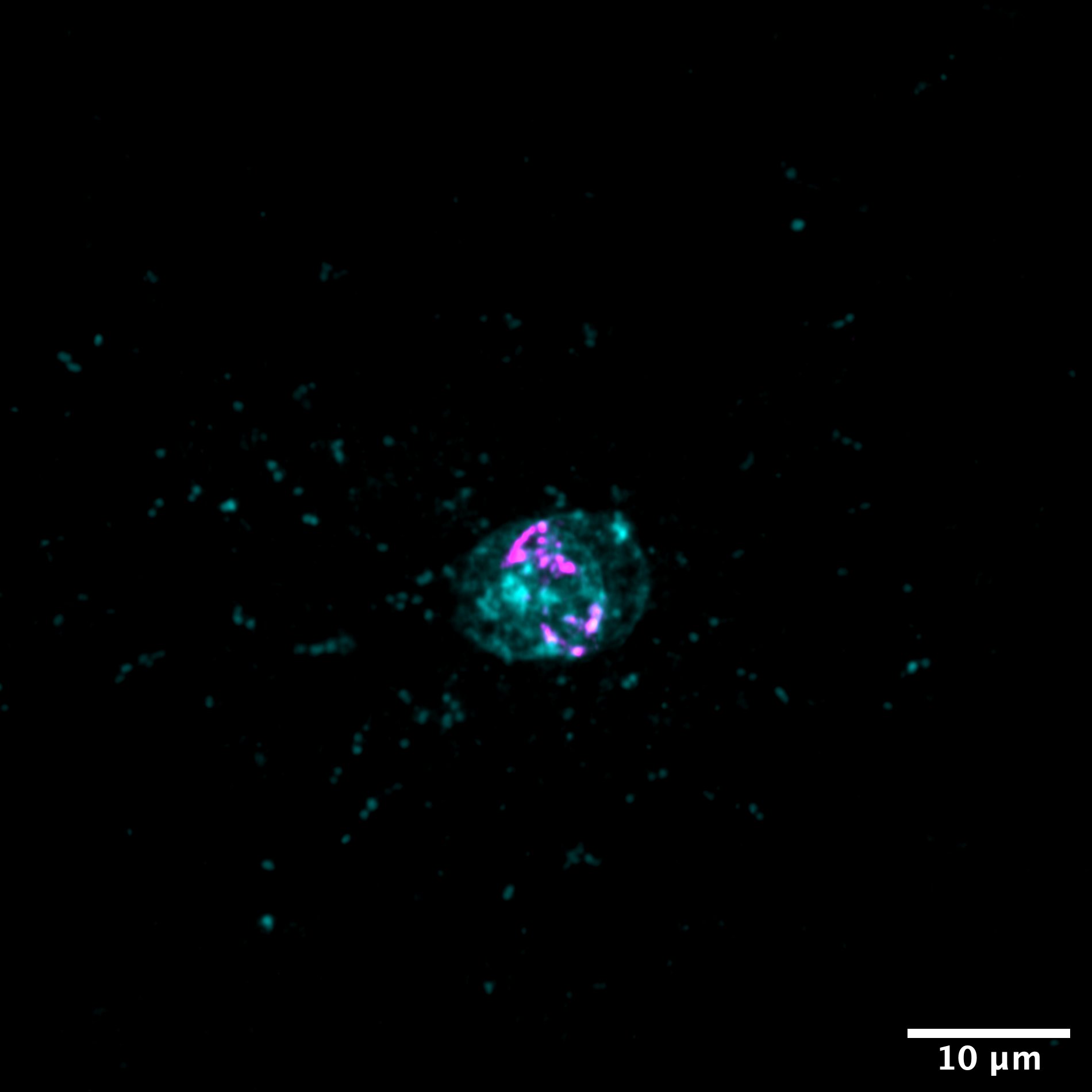

Supplement: Supplementary file 9 — Source data Fig. 2 [file 44318_2024_127_MOESM9_ESM.zip › figure2/figure2c/figure 2c_TM_H3K9me3_DAPI_apposition.jpg]

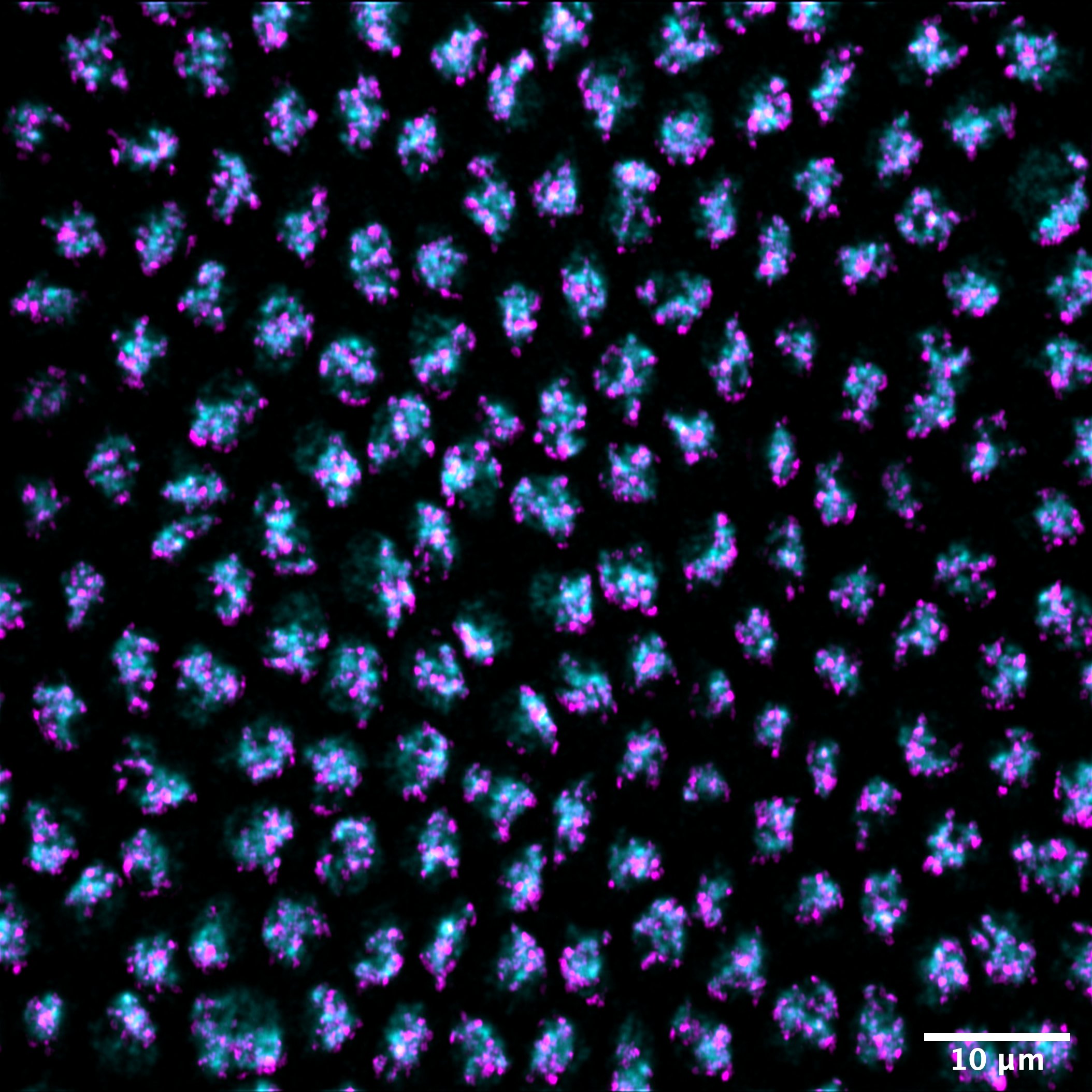

Supplement: Supplementary file 9 — Source data Fig. 2 [file 44318_2024_127_MOESM9_ESM.zip › figure2/figure2c/figure 2c_ctr_H3k9me3_DAPI_cycle14.jpg]

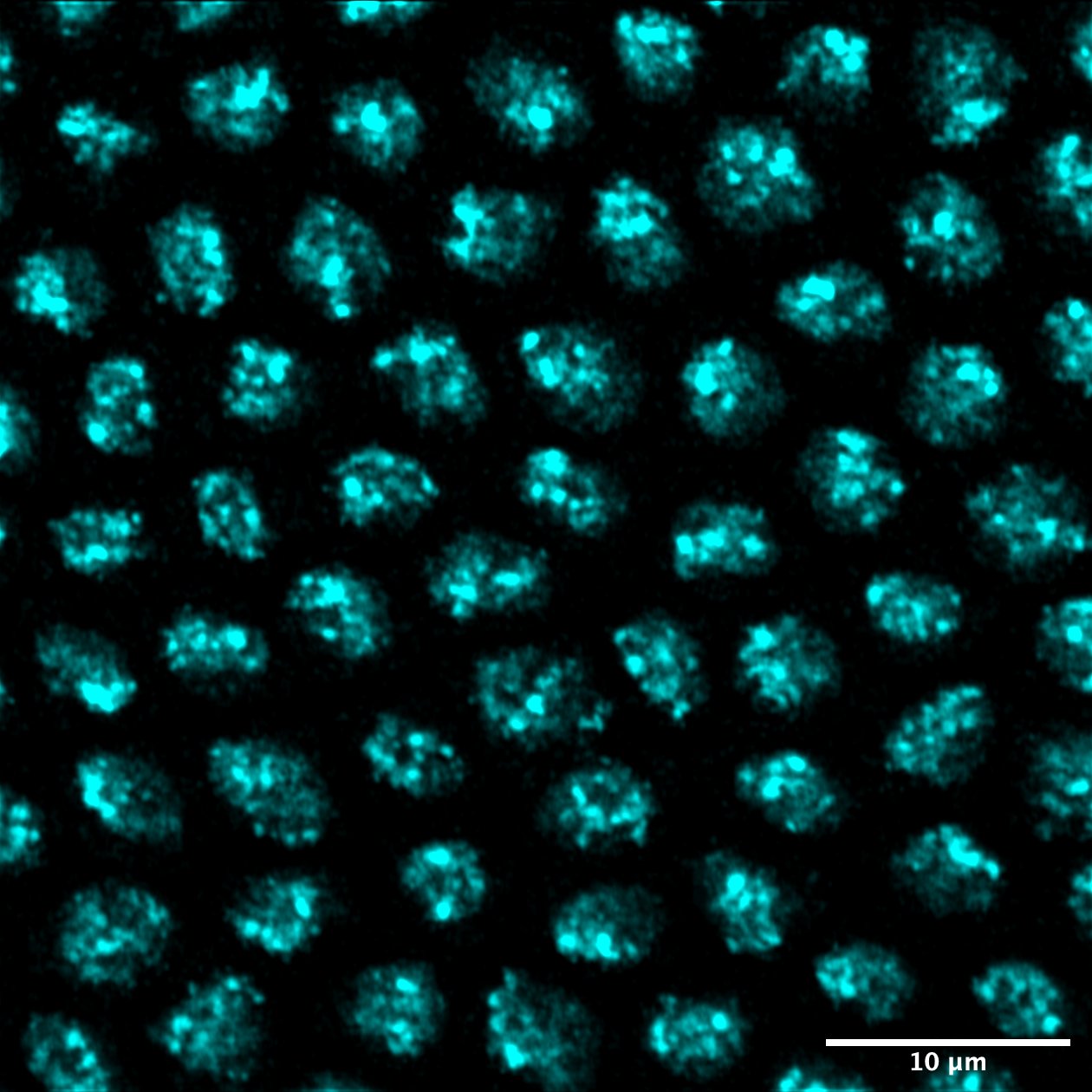

Supplement: Supplementary file 10 — Source data Fig. 3 [file 44318_2024_127_MOESM10_ESM.zip › figure3/figure3d/figure 3d_ctr_DAPI_cycle14.jpg]

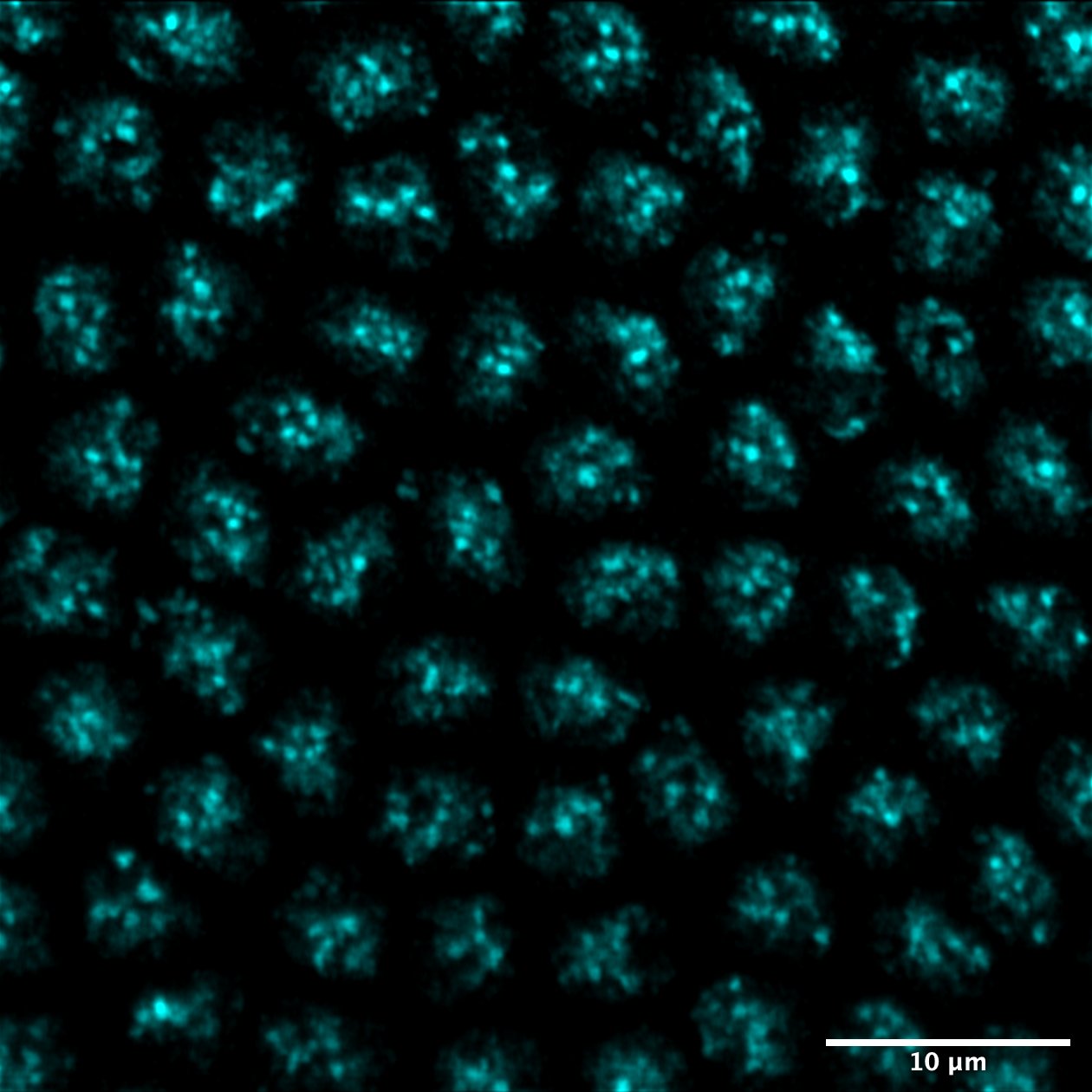

Supplement: Supplementary file 10 — Source data Fig. 3 [file 44318_2024_127_MOESM10_ESM.zip › figure3/figure3d/figure 3d_TM_DAPI_cycle14.jpg]

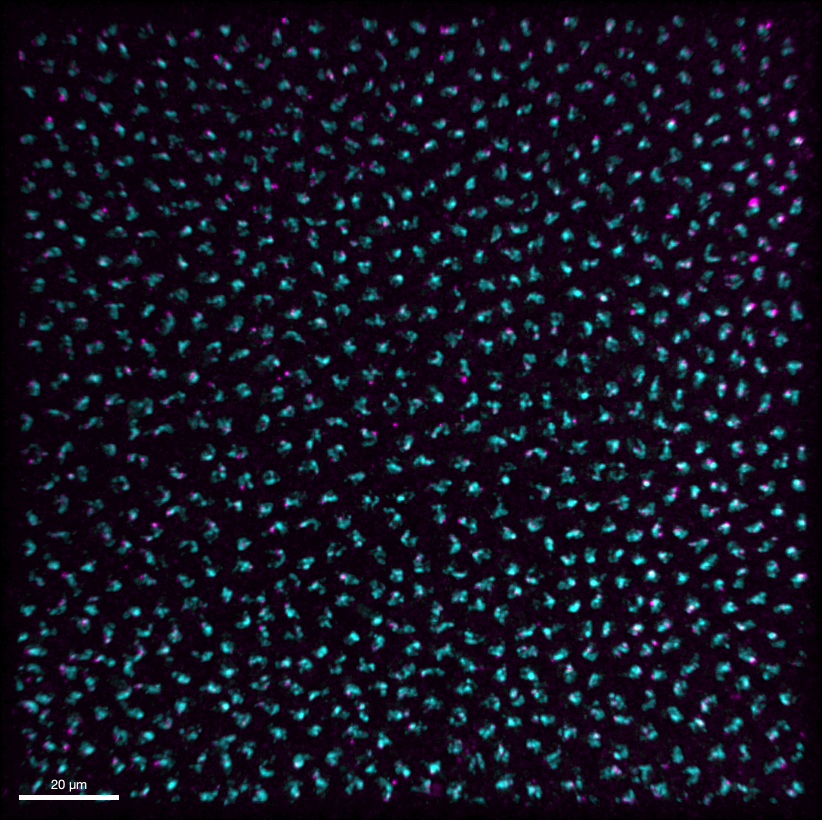

Supplement: Supplementary file 10 — Source data Fig. 3 [file 44318_2024_127_MOESM10_ESM.zip › figure3/figure3c/figure 3c_ctr_SATIII_DAPI_cycle14.tif]

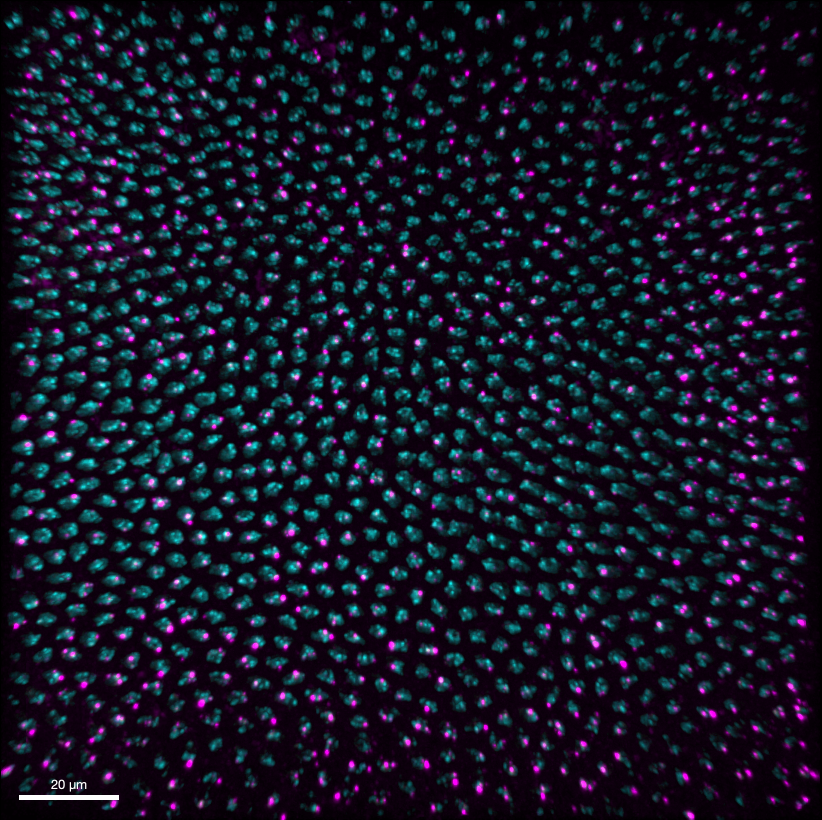

Supplement: Supplementary file 10 — Source data Fig. 3 [file 44318_2024_127_MOESM10_ESM.zip › figure3/figure3c/figure 3c_TM_SATIII_DAPI_cycle14.tif]

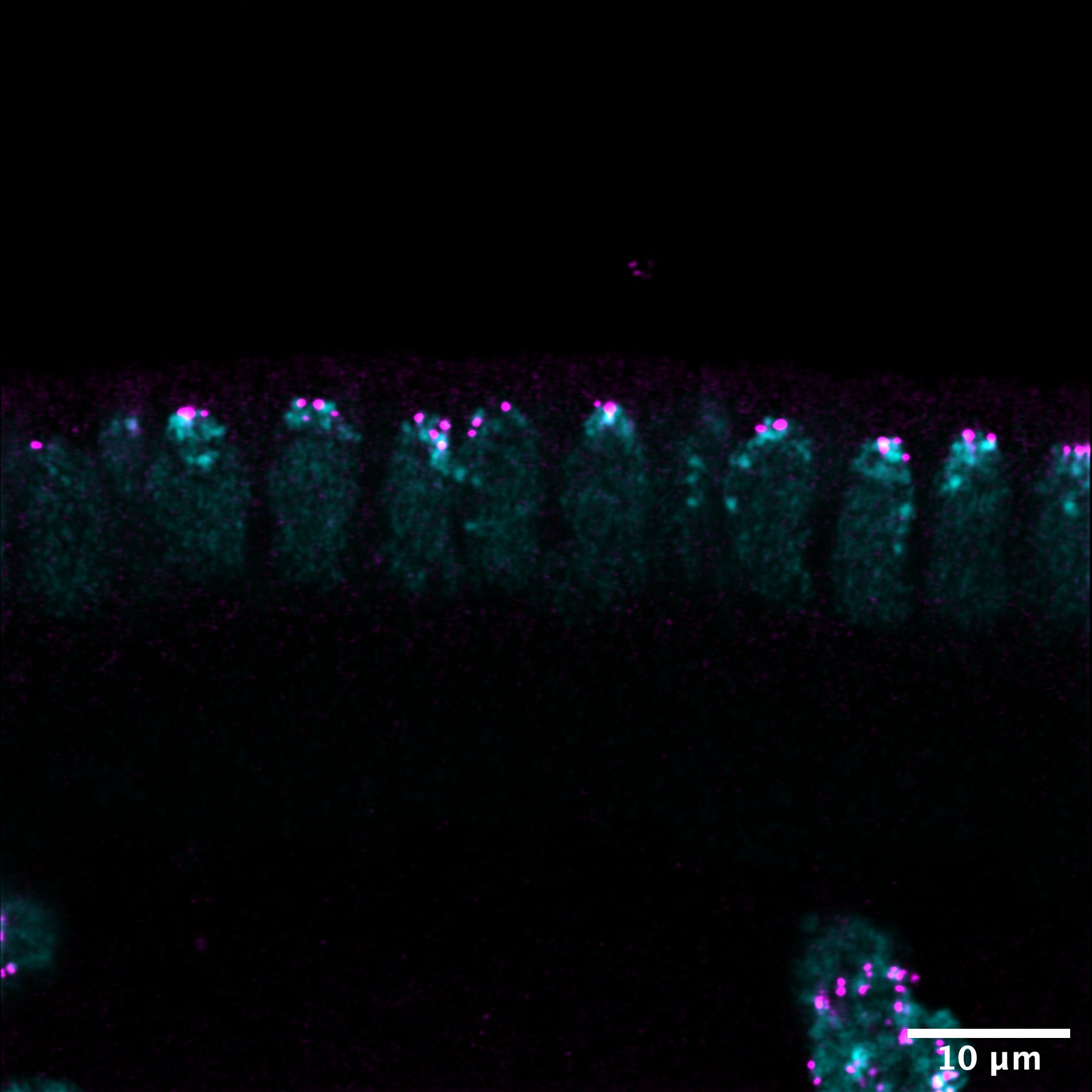

Supplement: Supplementary file 10 — Source data Fig. 3 [file 44318_2024_127_MOESM10_ESM.zip › figure3/figure3e/figure 3e_ctr_CID_DAPI_cycle 14.jpg]

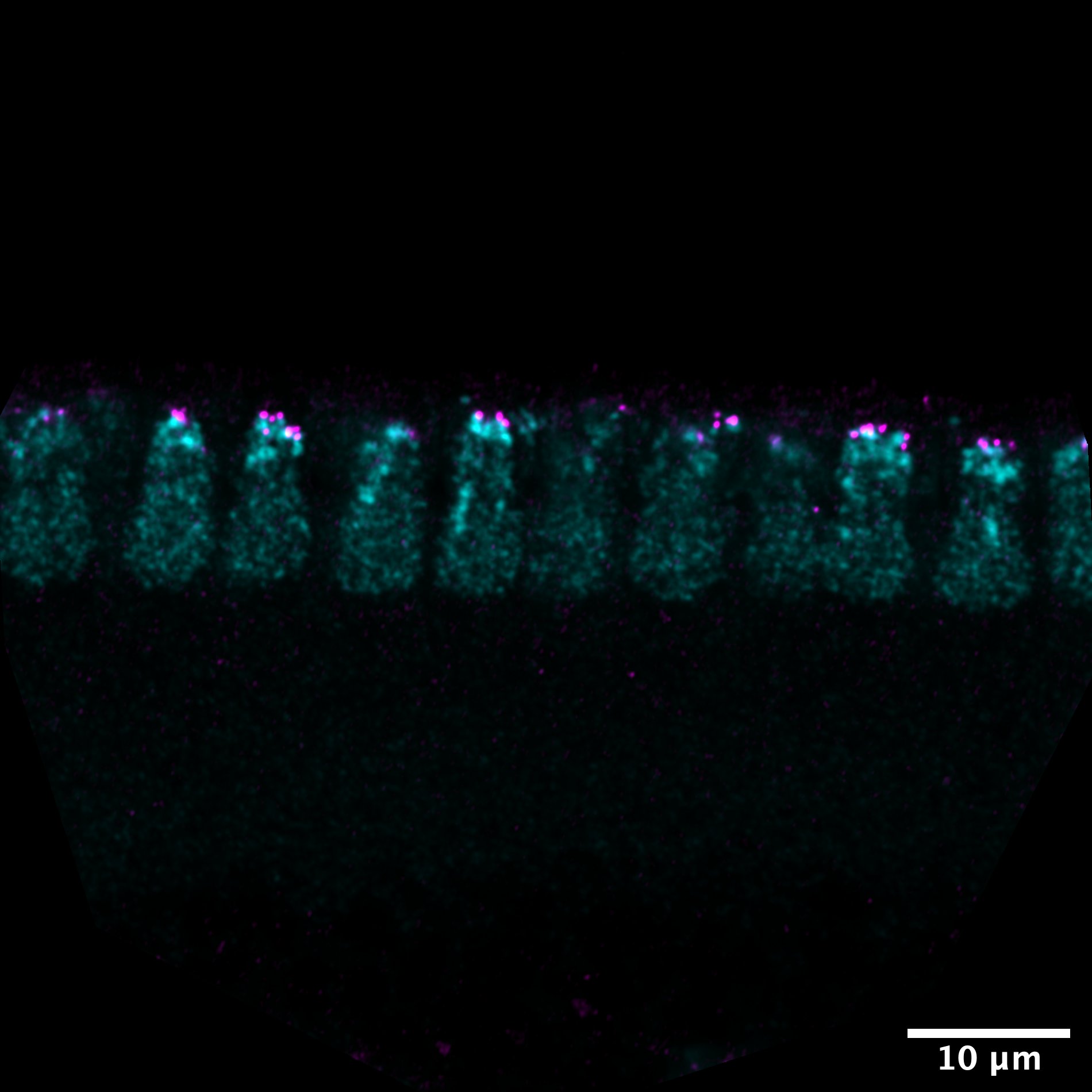

Supplement: Supplementary file 10 — Source data Fig. 3 [file 44318_2024_127_MOESM10_ESM.zip › figure3/figure3e/figure 3e_TM_CID_DAPI_cycle 14.jpg]

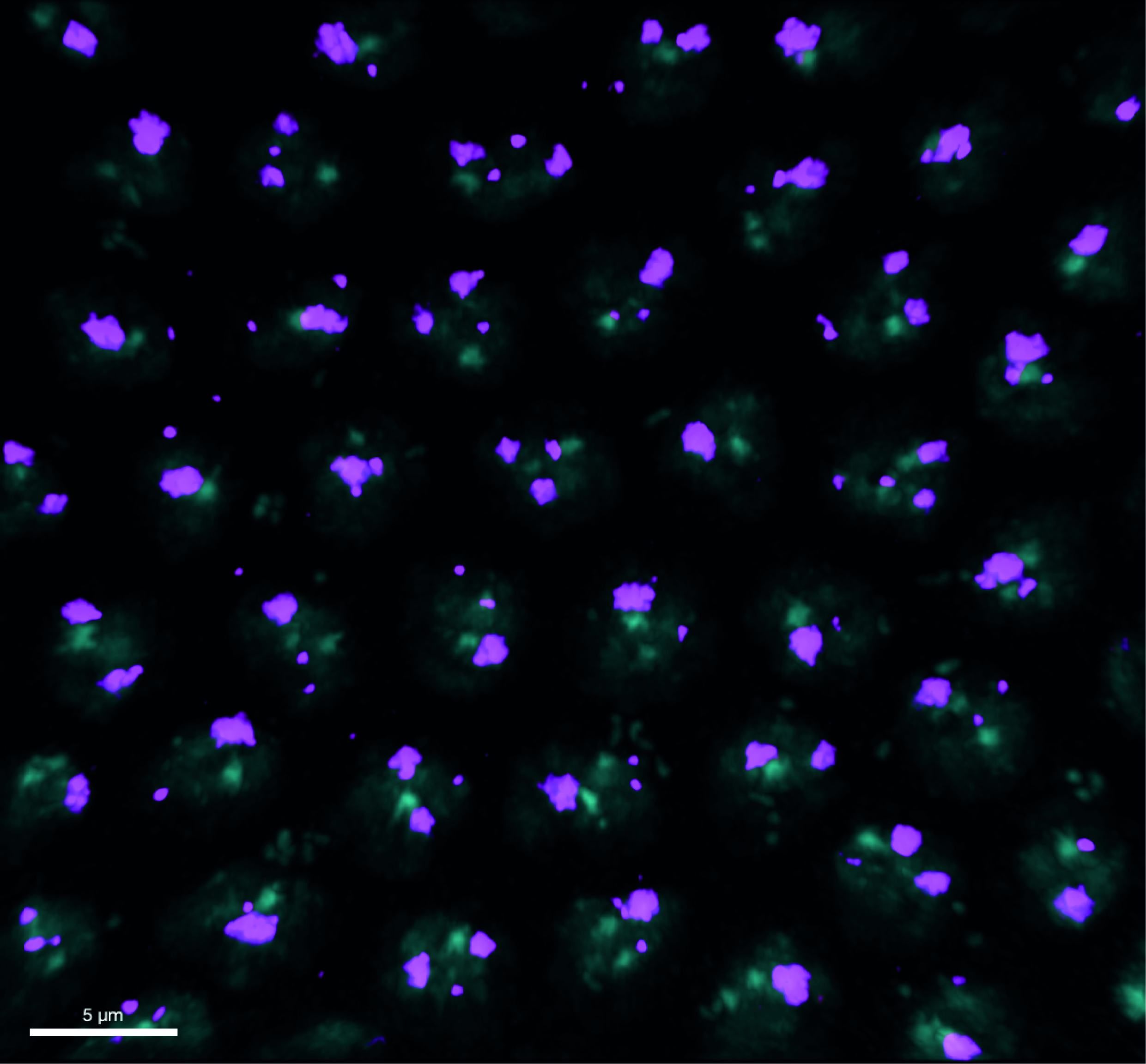

Supplement: Supplementary file 10 — Source data Fig. 3 [file 44318_2024_127_MOESM10_ESM.zip › figure3/figure3f/figure 3f_TM_SATIII_DNAFISH_cycle14.tif]

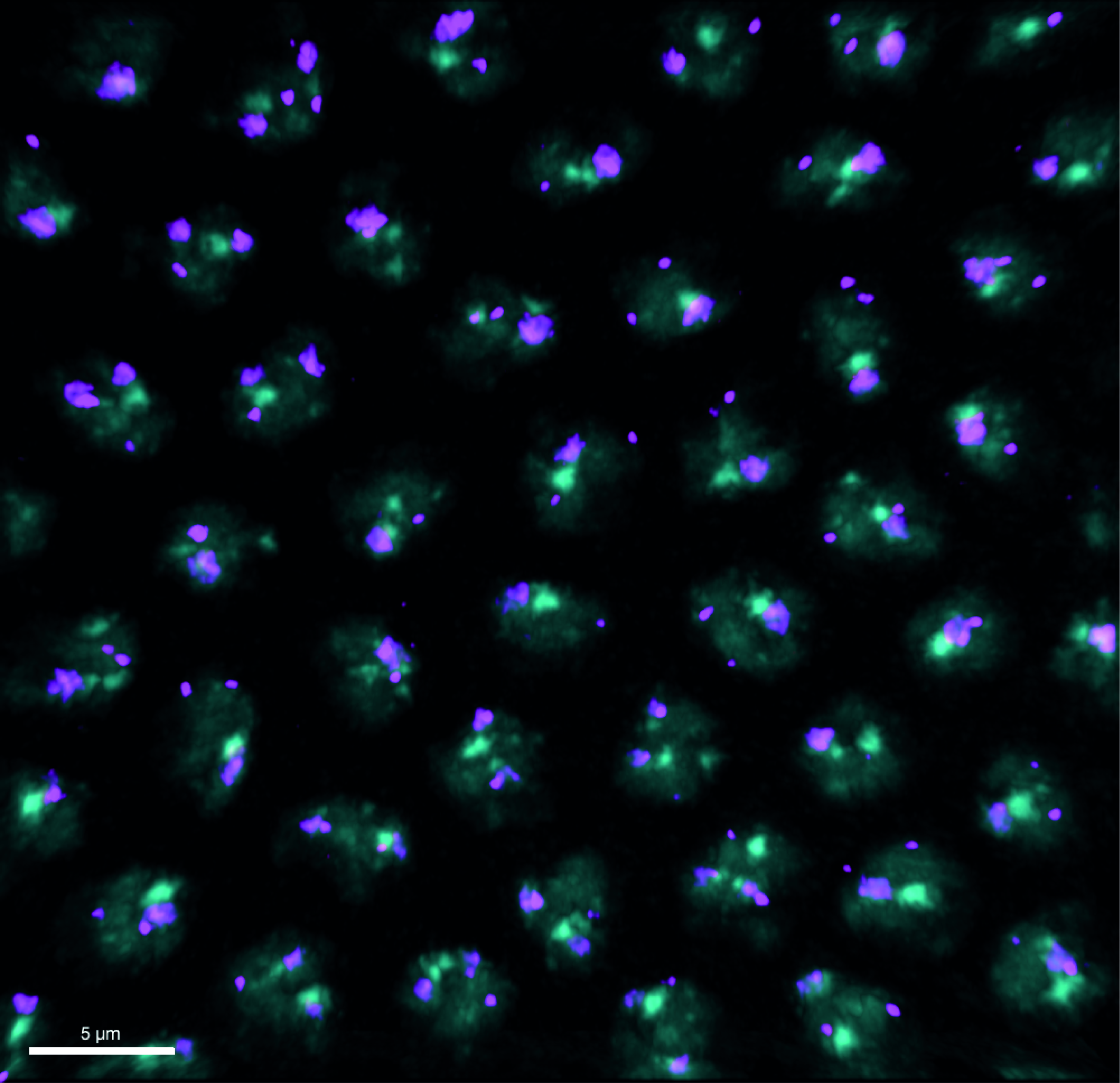

Supplement: Supplementary file 10 — Source data Fig. 3 [file 44318_2024_127_MOESM10_ESM.zip › figure3/figure3f/figure 3f_ctr_SATIII_DNAFISH_cycle14.tif]

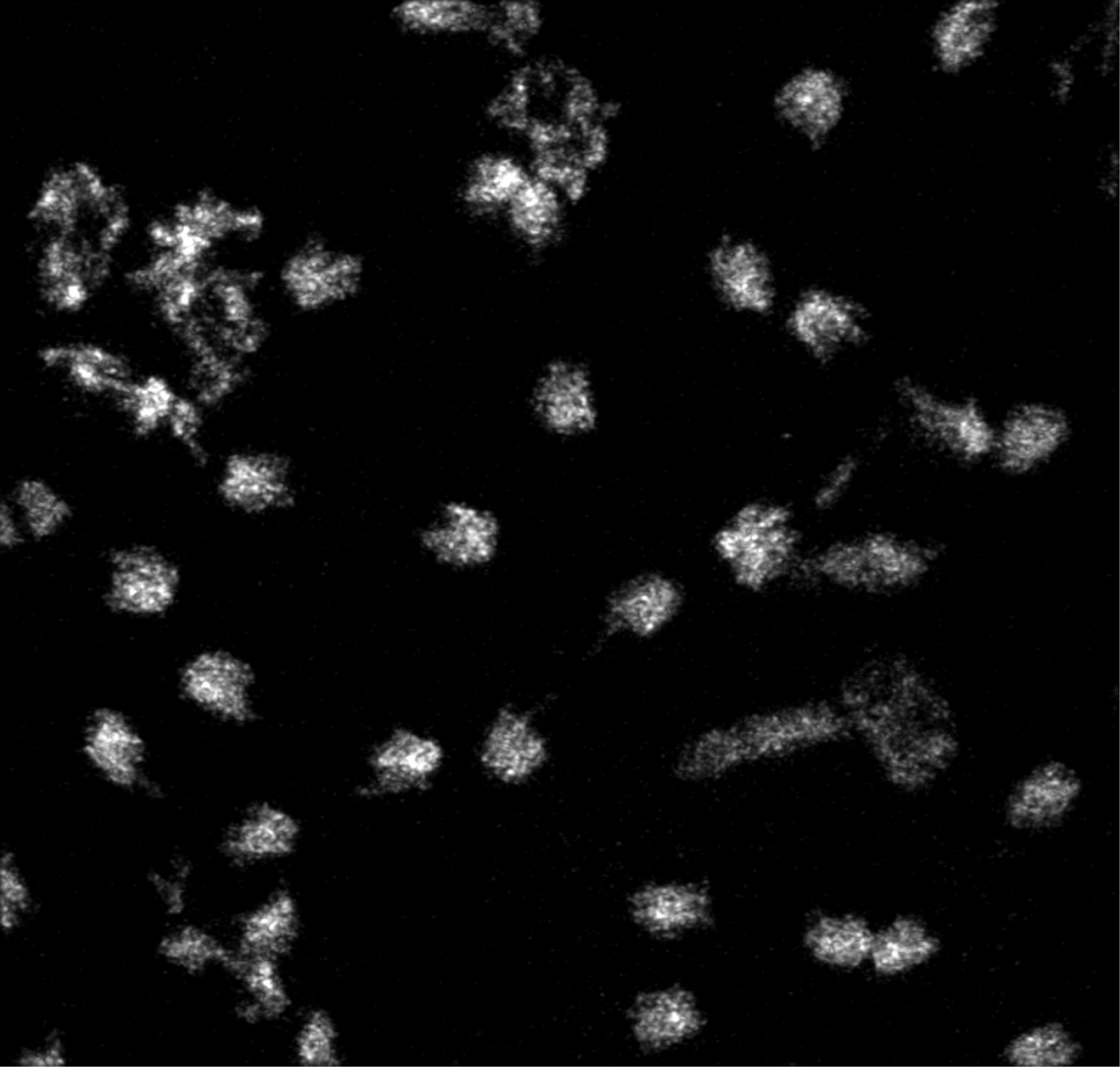

Supplement: Supplementary file 10 — Source data Fig. 3 [file 44318_2024_127_MOESM10_ESM.zip › figure3/figure3a/figure 3a_multipolar.tif]

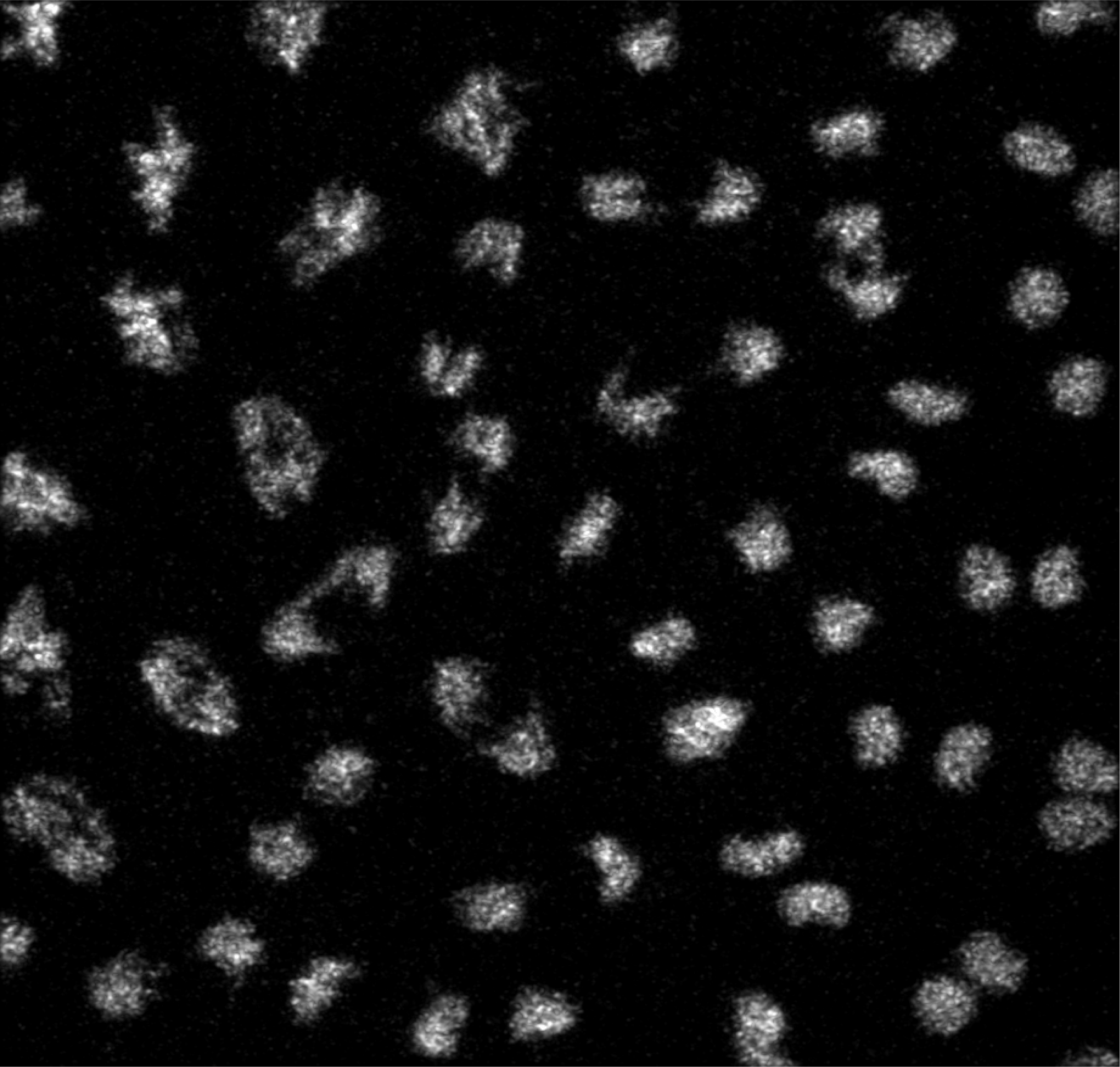

Supplement: Supplementary file 10 — Source data Fig. 3 [file 44318_2024_127_MOESM10_ESM.zip › figure3/figure3a/figure 3a_bridge.tif]

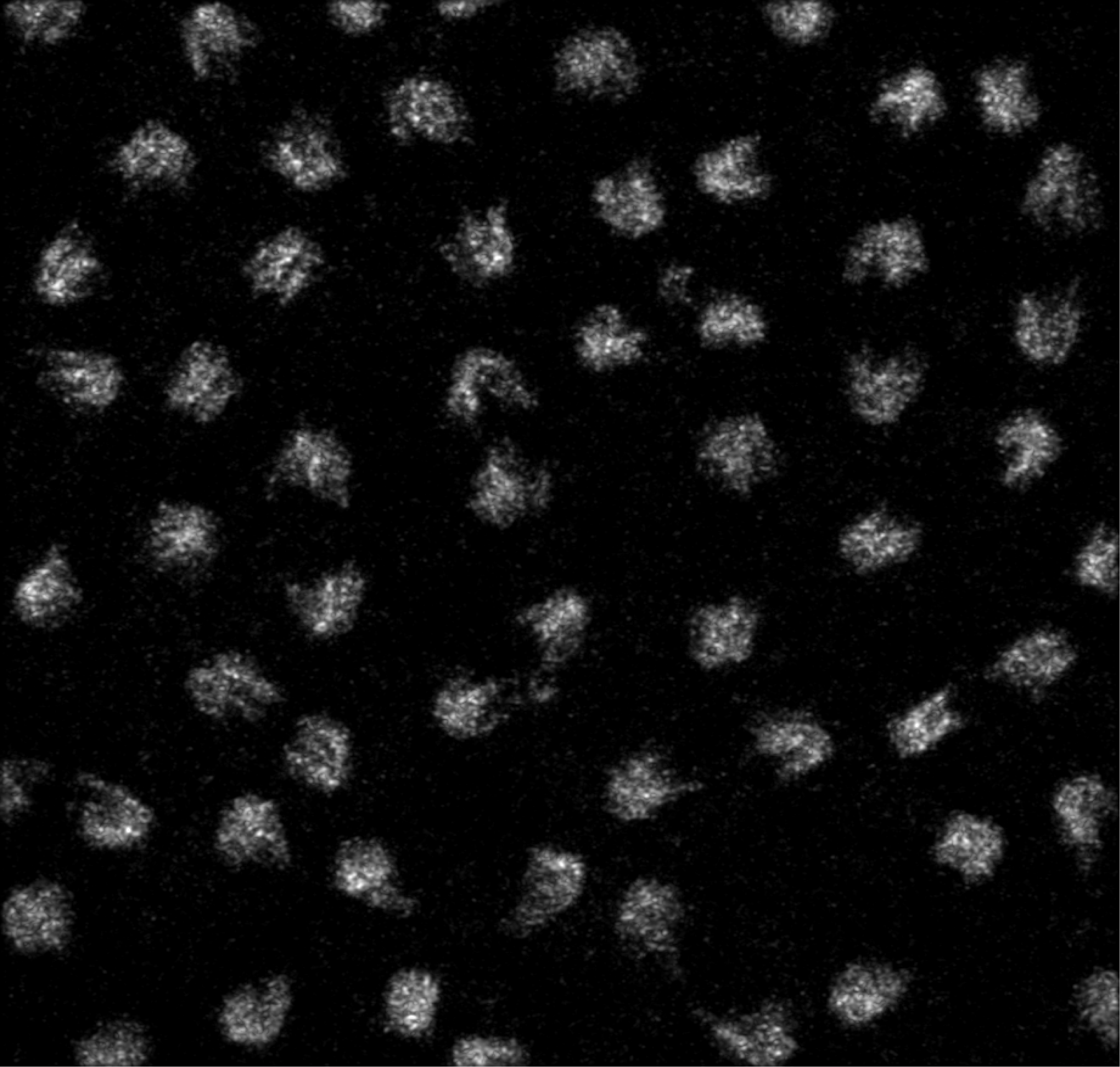

Supplement: Supplementary file 10 — Source data Fig. 3 [file 44318_2024_127_MOESM10_ESM.zip › figure3/figure3a/figure 3a_lagging.tif]

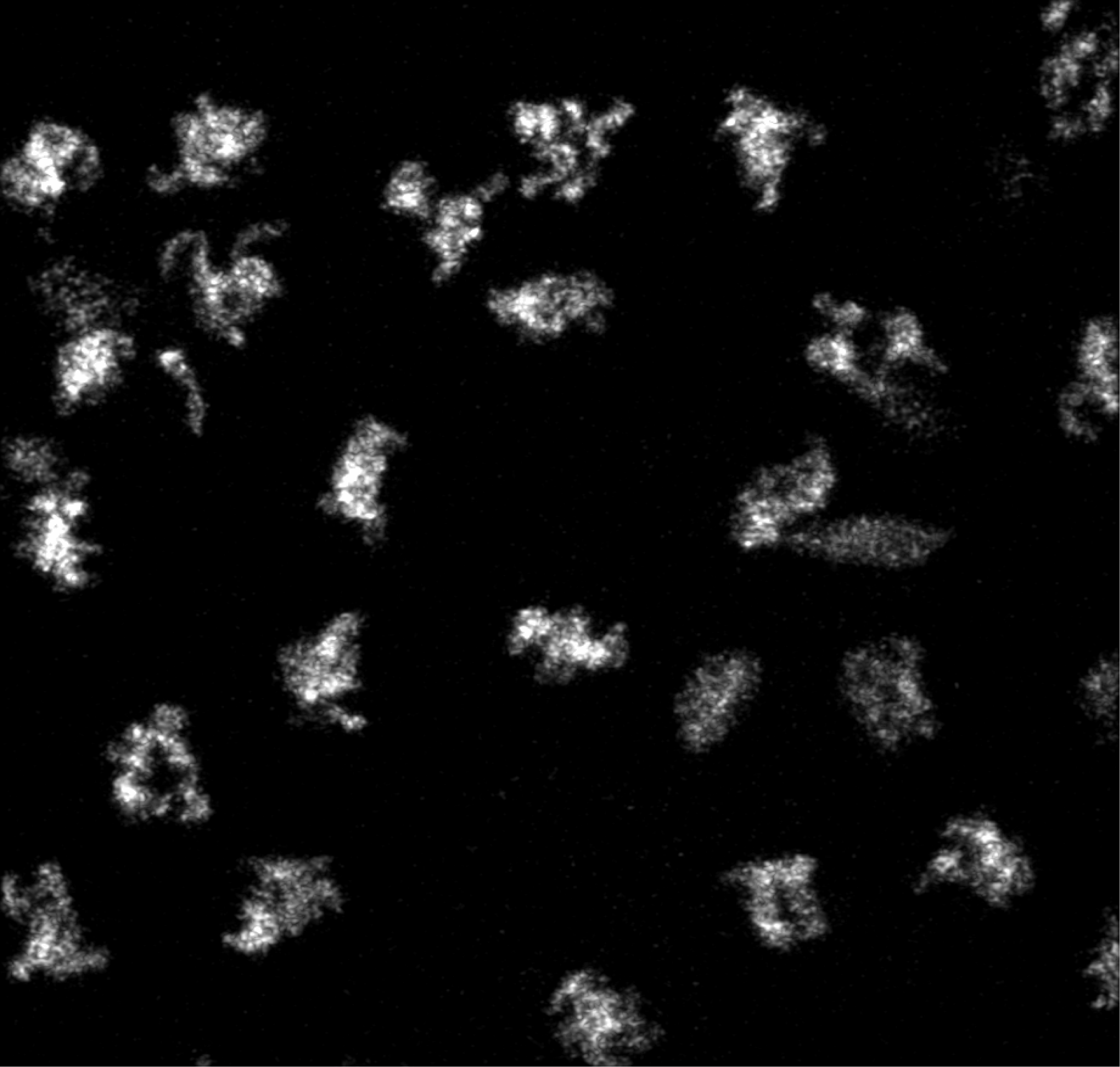

Supplement: Supplementary file 10 — Source data Fig. 3 [file 44318_2024_127_MOESM10_ESM.zip › figure3/figure3a/figure 3a_non-condensed.tif]

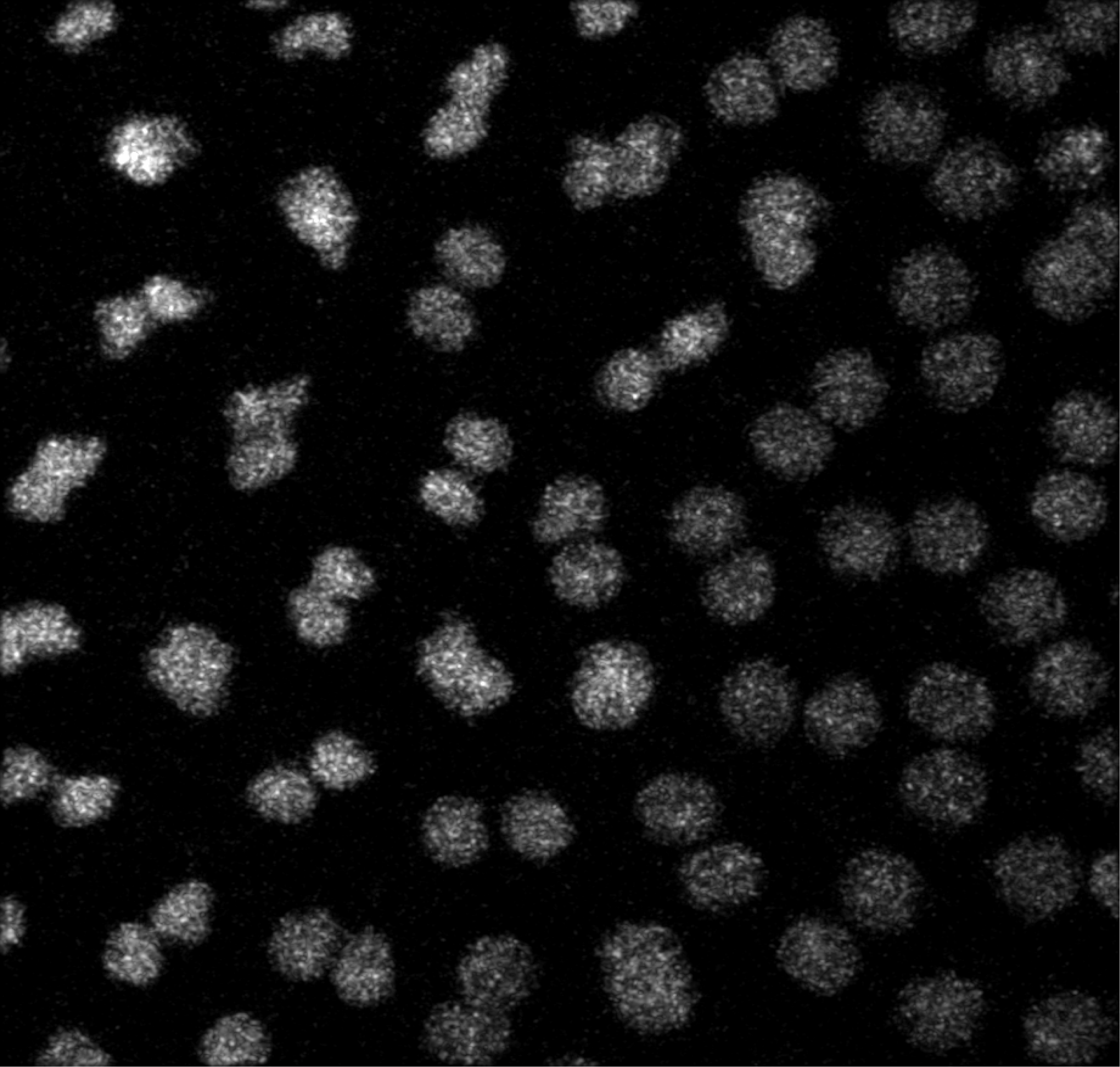

Supplement: Supplementary file 10 — Source data Fig. 3 [file 44318_2024_127_MOESM10_ESM.zip › figure3/figure3a/figure 3a_normal cytokinesis.tif]

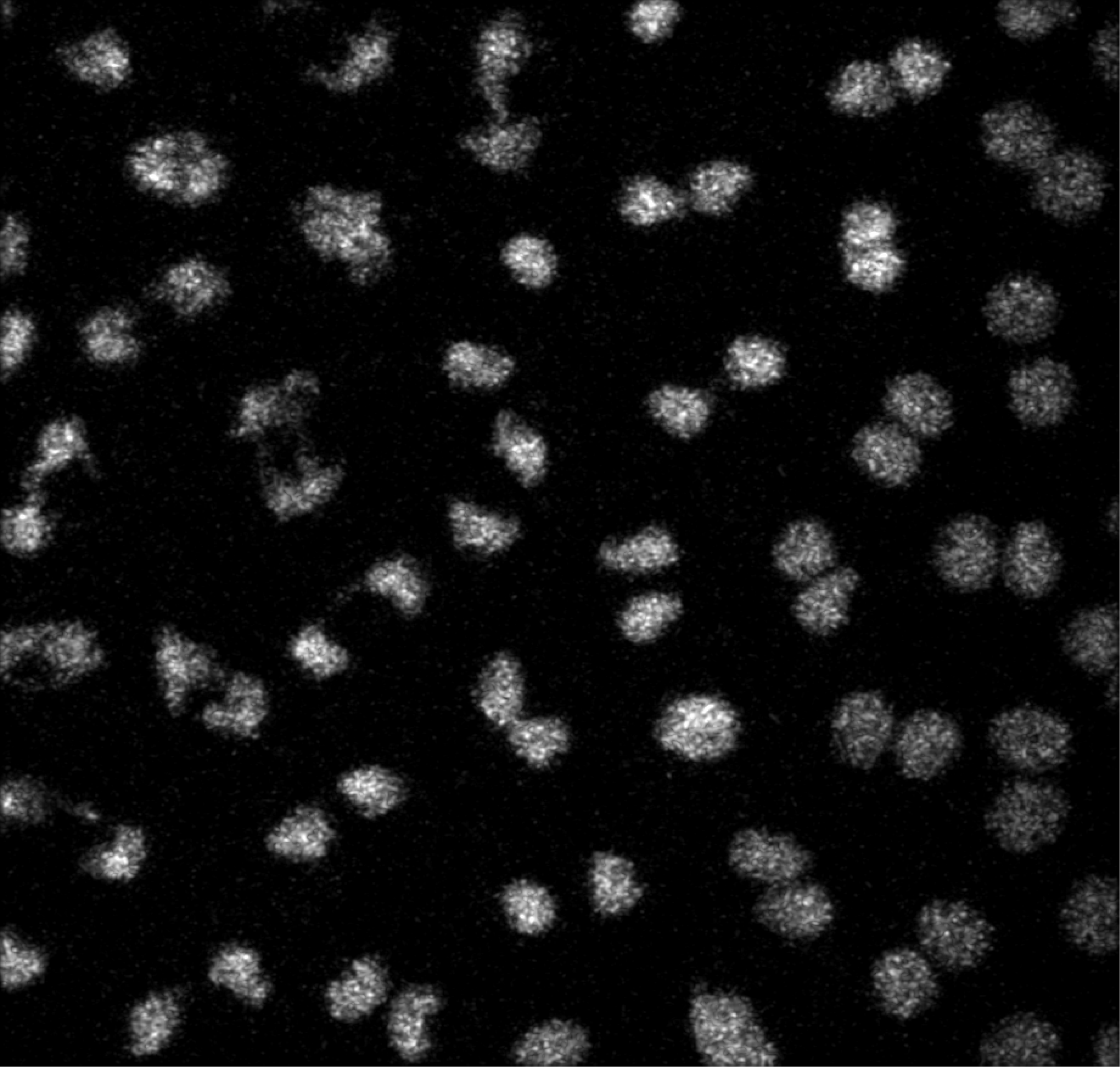

Supplement: Supplementary file 10 — Source data Fig. 3 [file 44318_2024_127_MOESM10_ESM.zip › figure3/figure3a/figure 3a_arrest.tif]

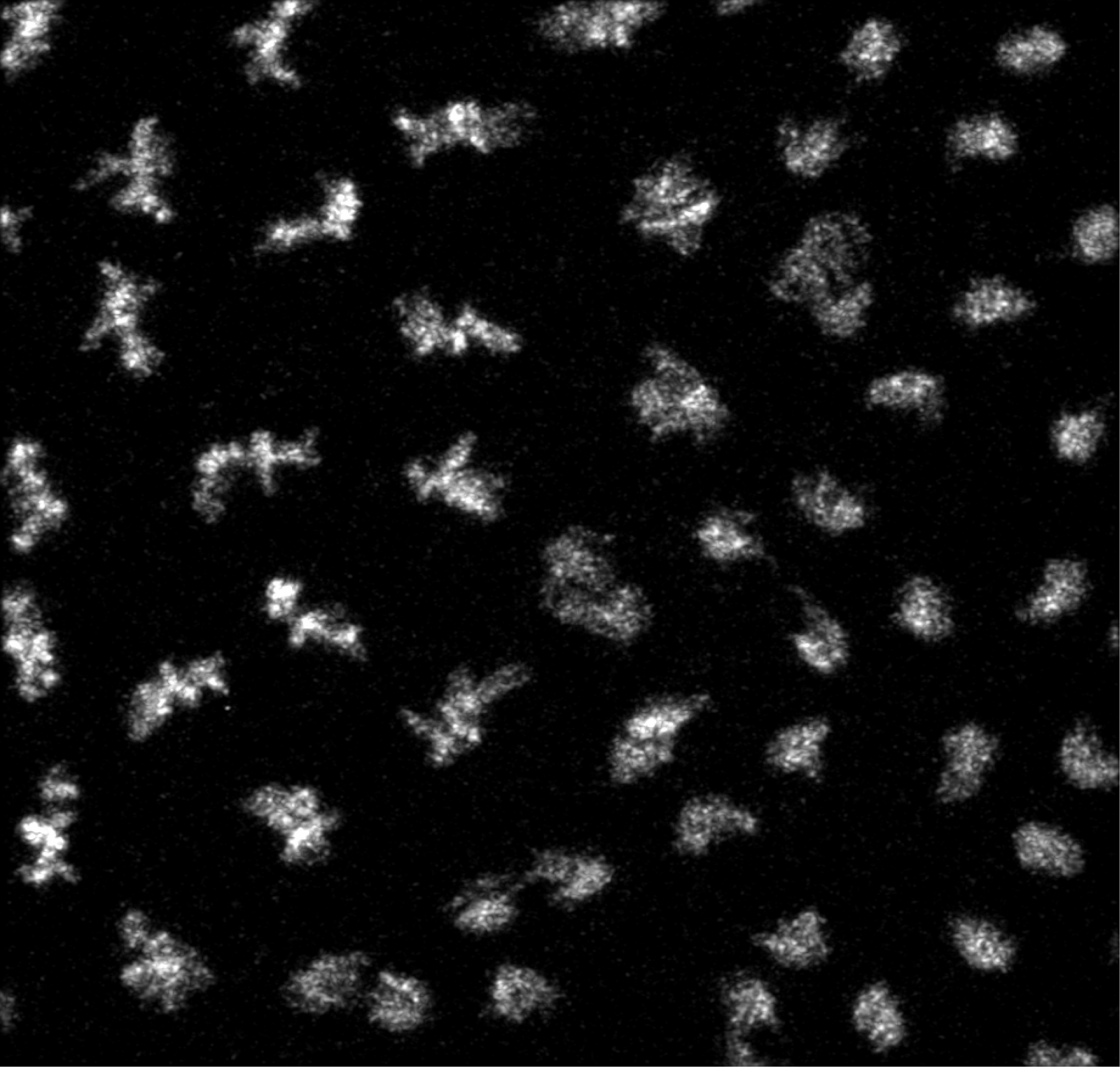

Supplement: Supplementary file 10 — Source data Fig. 3 [file 44318_2024_127_MOESM10_ESM.zip › figure3/figure3a/figure 3a_fibre.tif]

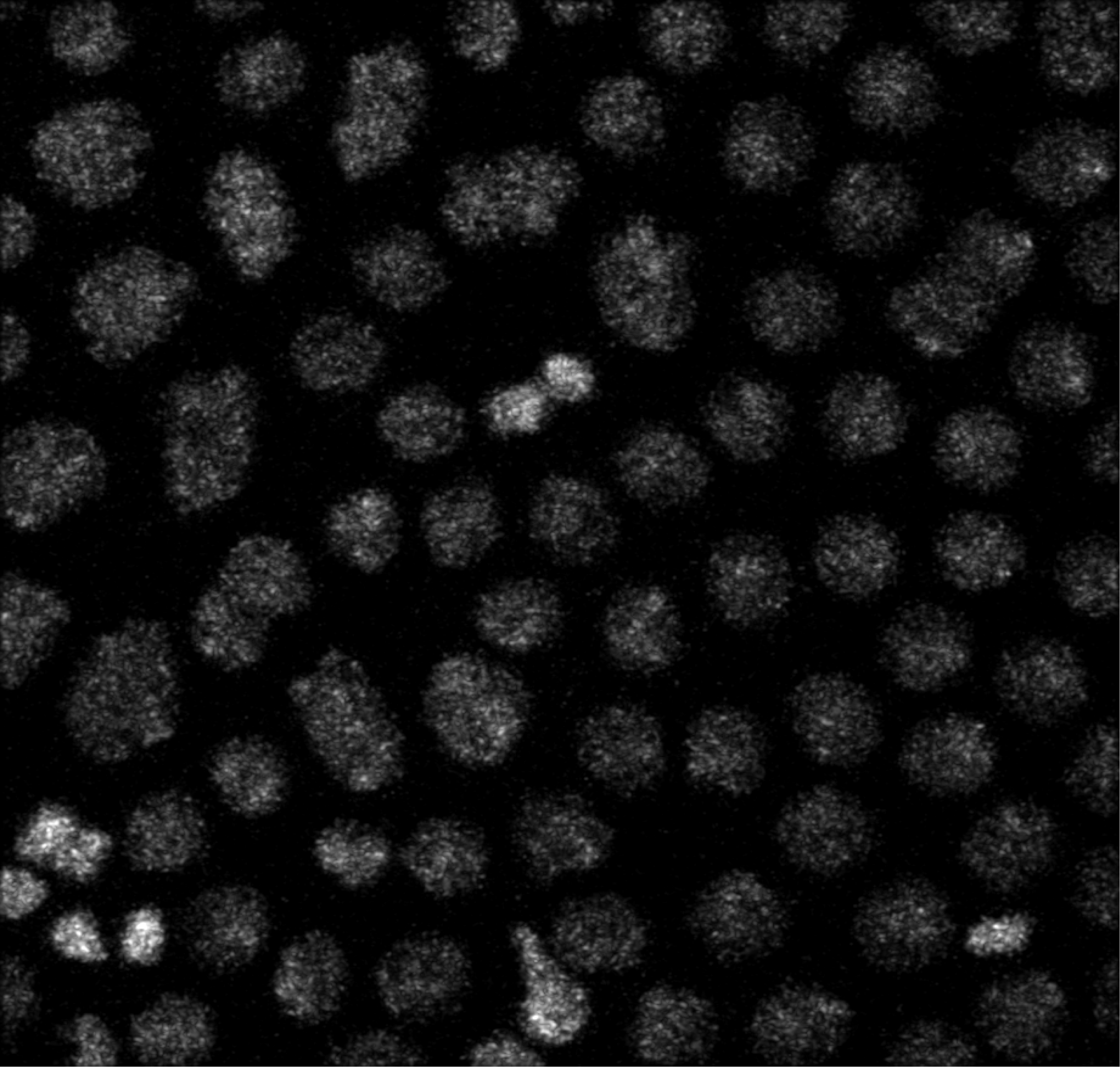

Supplement: Supplementary file 10 — Source data Fig. 3 [file 44318_2024_127_MOESM10_ESM.zip › figure3/figure3a/figure 3a_apoptosis.tif]

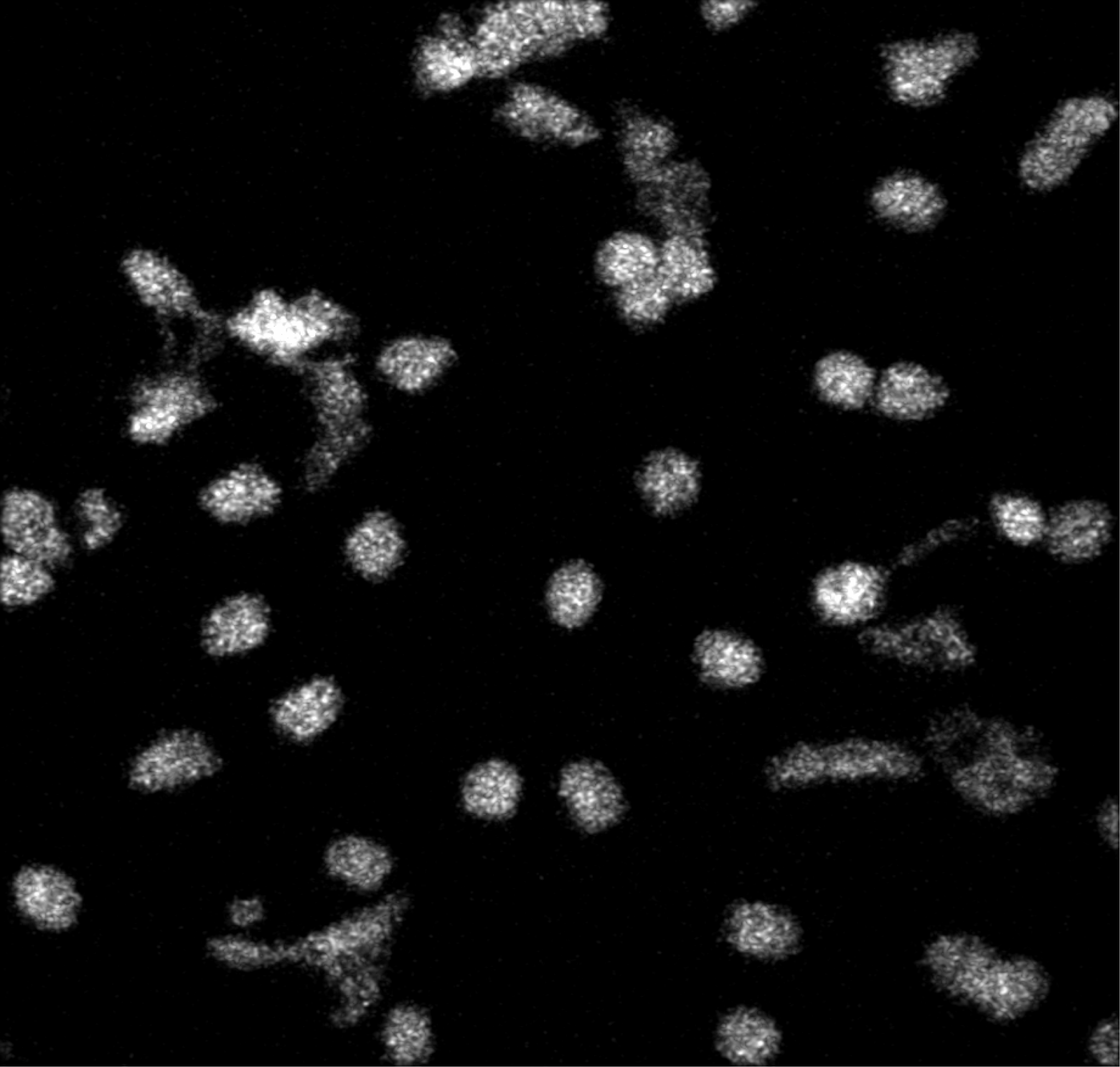

Supplement: Supplementary file 10 — Source data Fig. 3 [file 44318_2024_127_MOESM10_ESM.zip › figure3/figure3a/figure 3a_micronuclei.tif]

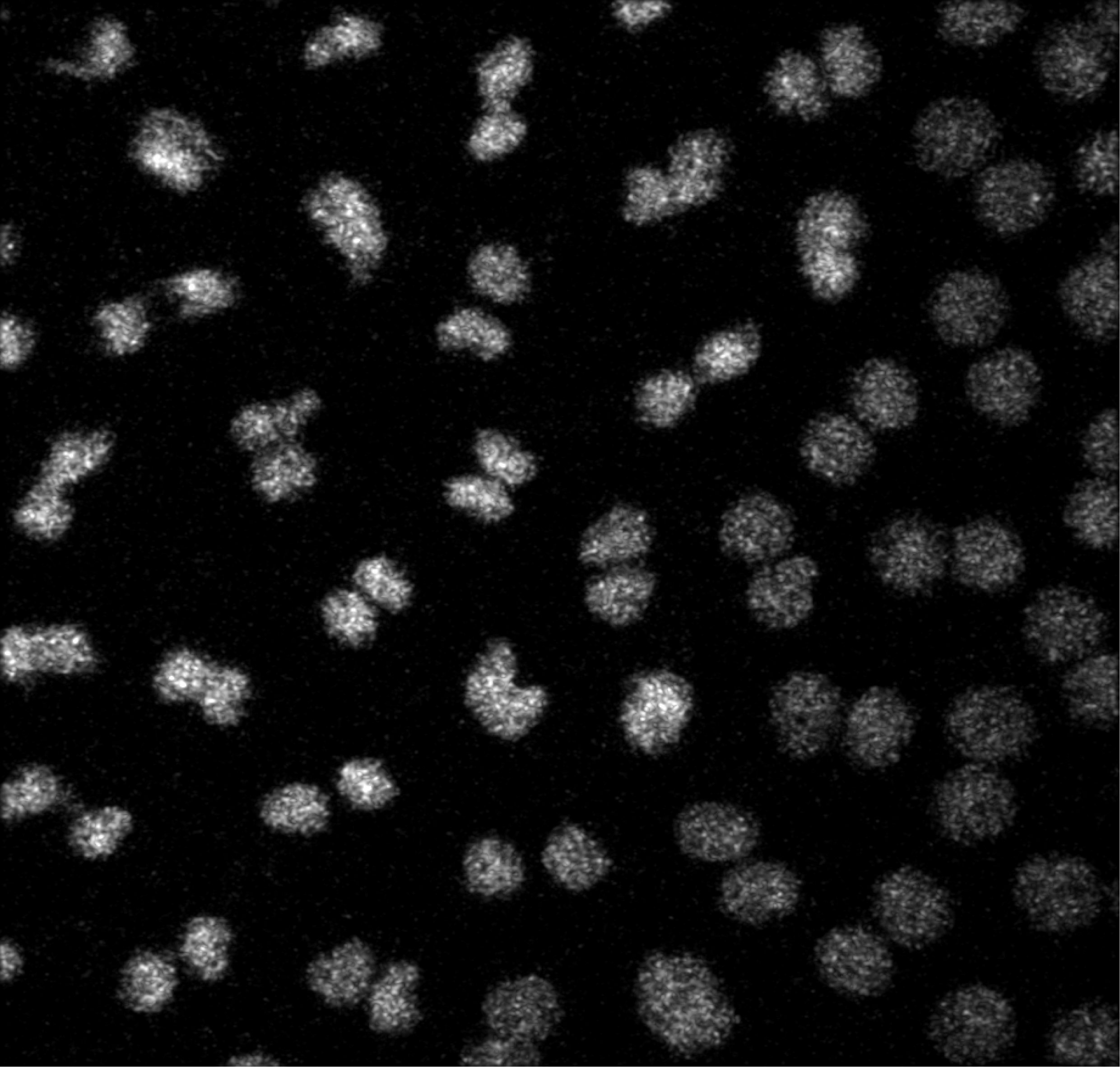

Supplement: Supplementary file 10 — Source data Fig. 3 [file 44318_2024_127_MOESM10_ESM.zip › figure3/figure3a/figure 3a_fusion.tif]

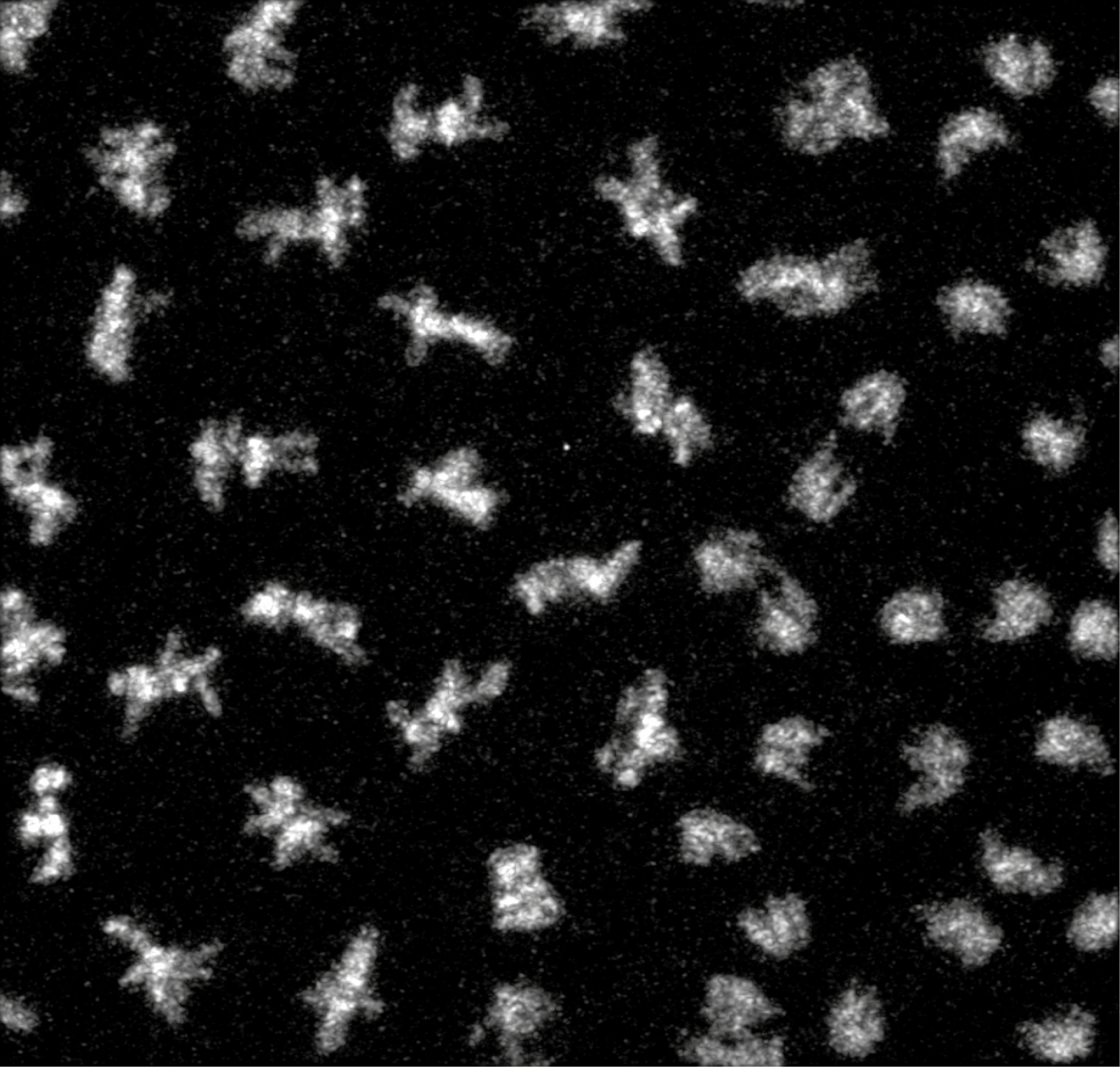

Supplement: Supplementary file 10 — Source data Fig. 3 [file 44318_2024_127_MOESM10_ESM.zip › figure3/figure3a/figure 3a_normal mitosis.tif]

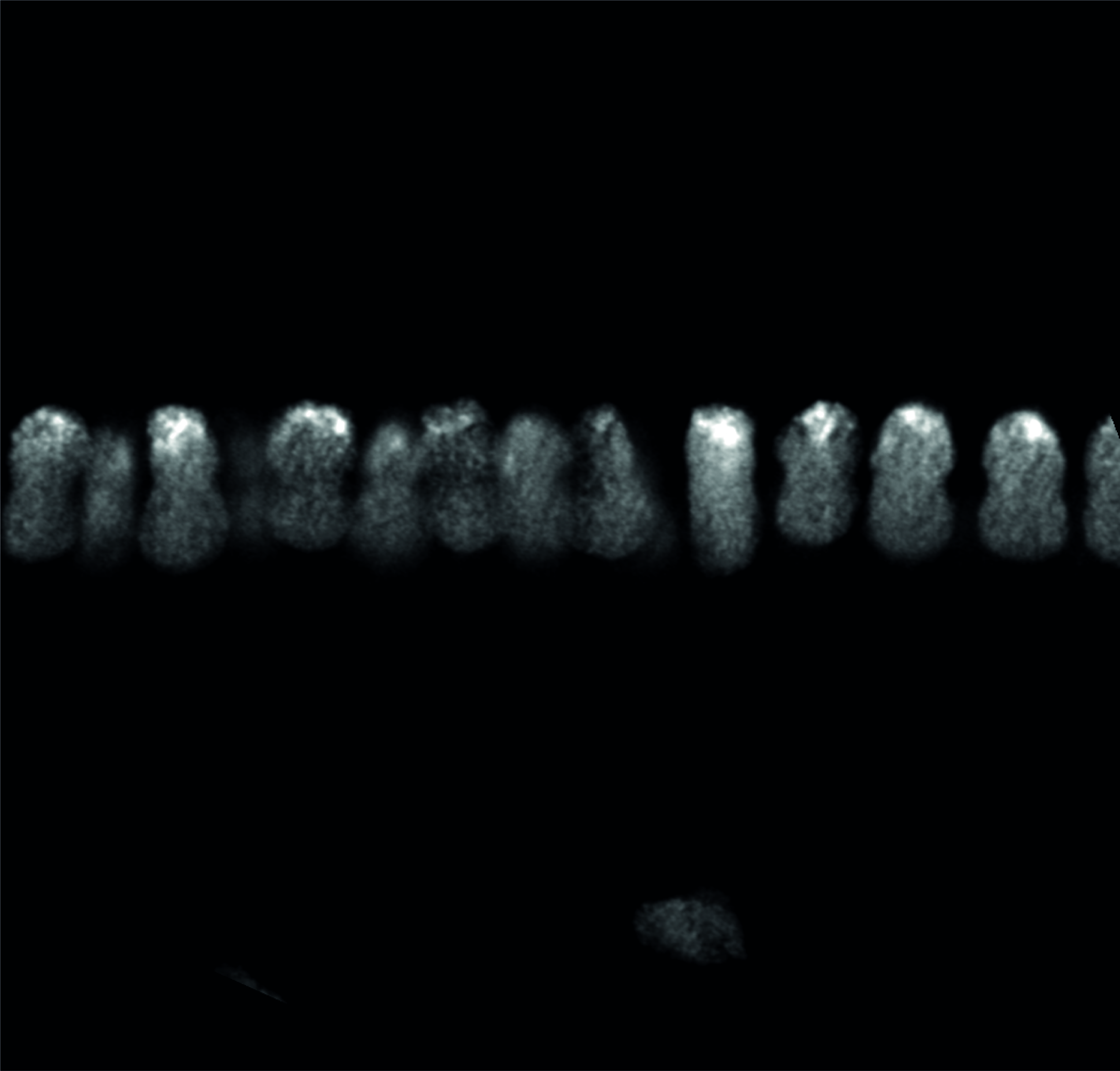

Supplement: Supplementary file 11 — Source data Fig. 4 [file 44318_2024_127_MOESM11_ESM.zip › figure4/figure4c/figure 4c_ctr_HP1_cycle14.tif]

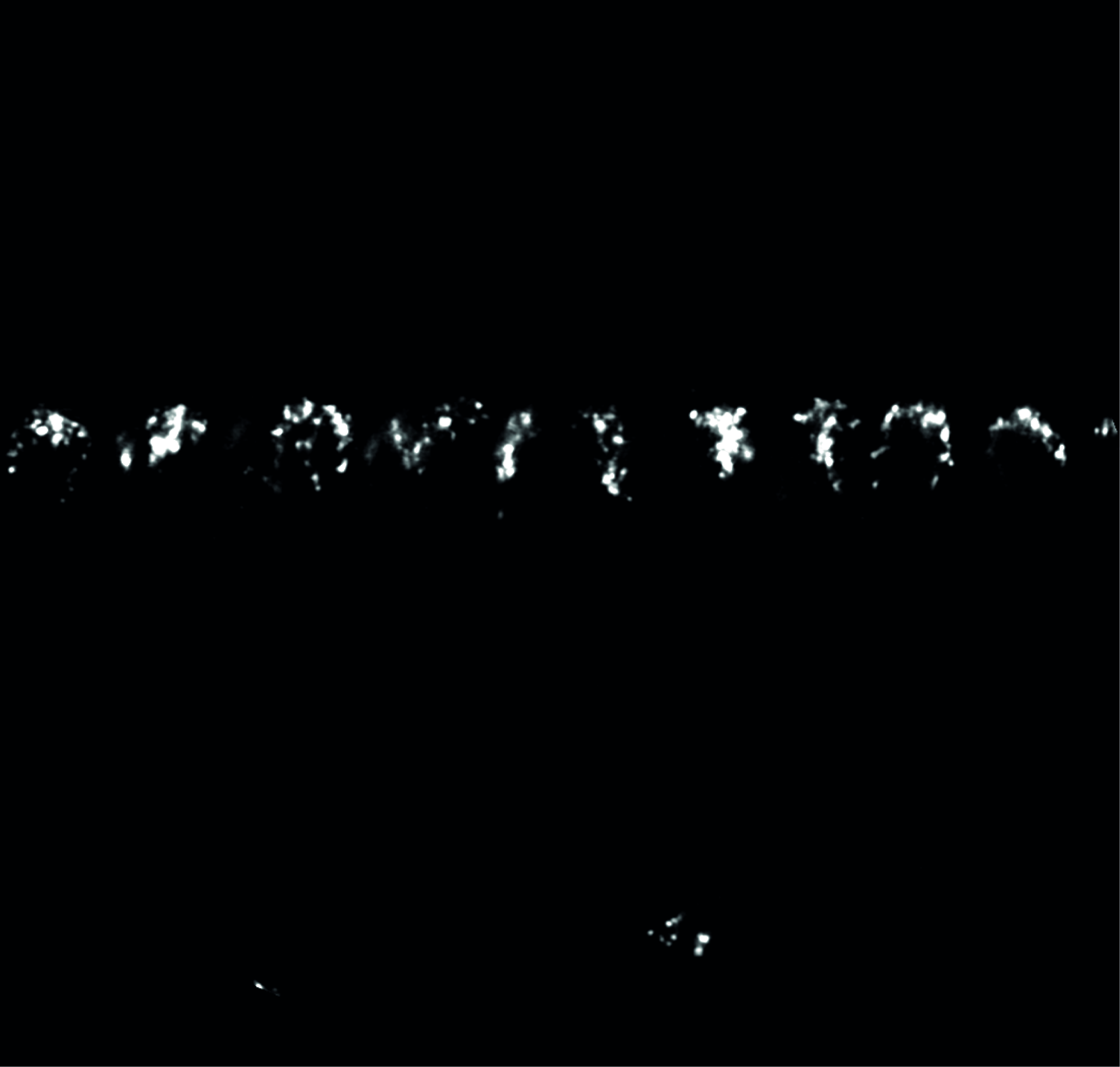

Supplement: Supplementary file 11 — Source data Fig. 4 [file 44318_2024_127_MOESM11_ESM.zip › figure4/figure4c/figure 4c_ctr_H3K9me3_cycle14.tif]

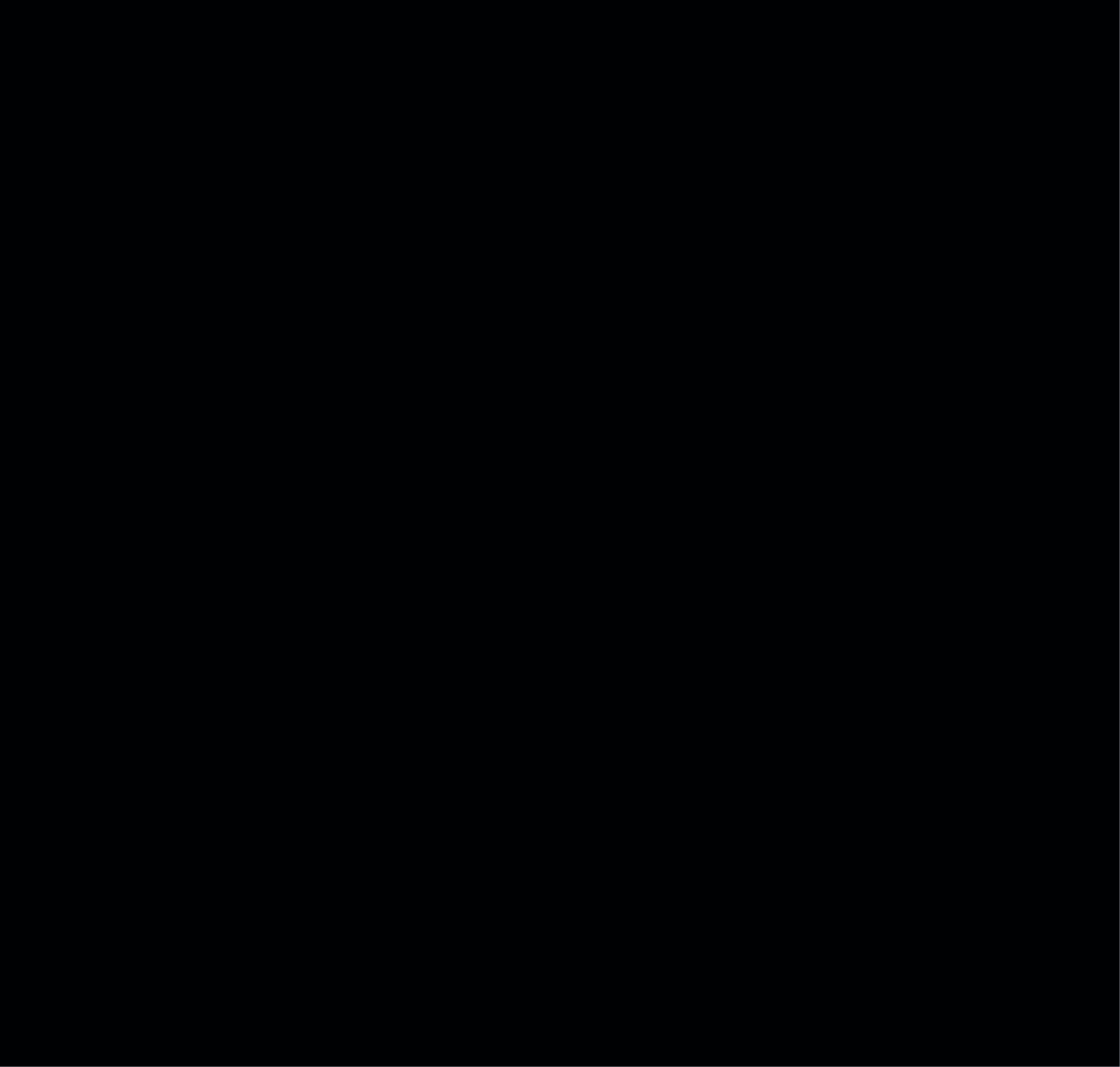

Supplement: Supplementary file 11 — Source data Fig. 4 [file 44318_2024_127_MOESM11_ESM.zip › figure4/figure4c/figure 4c_TM_H3K9me3_cycle14.tif]

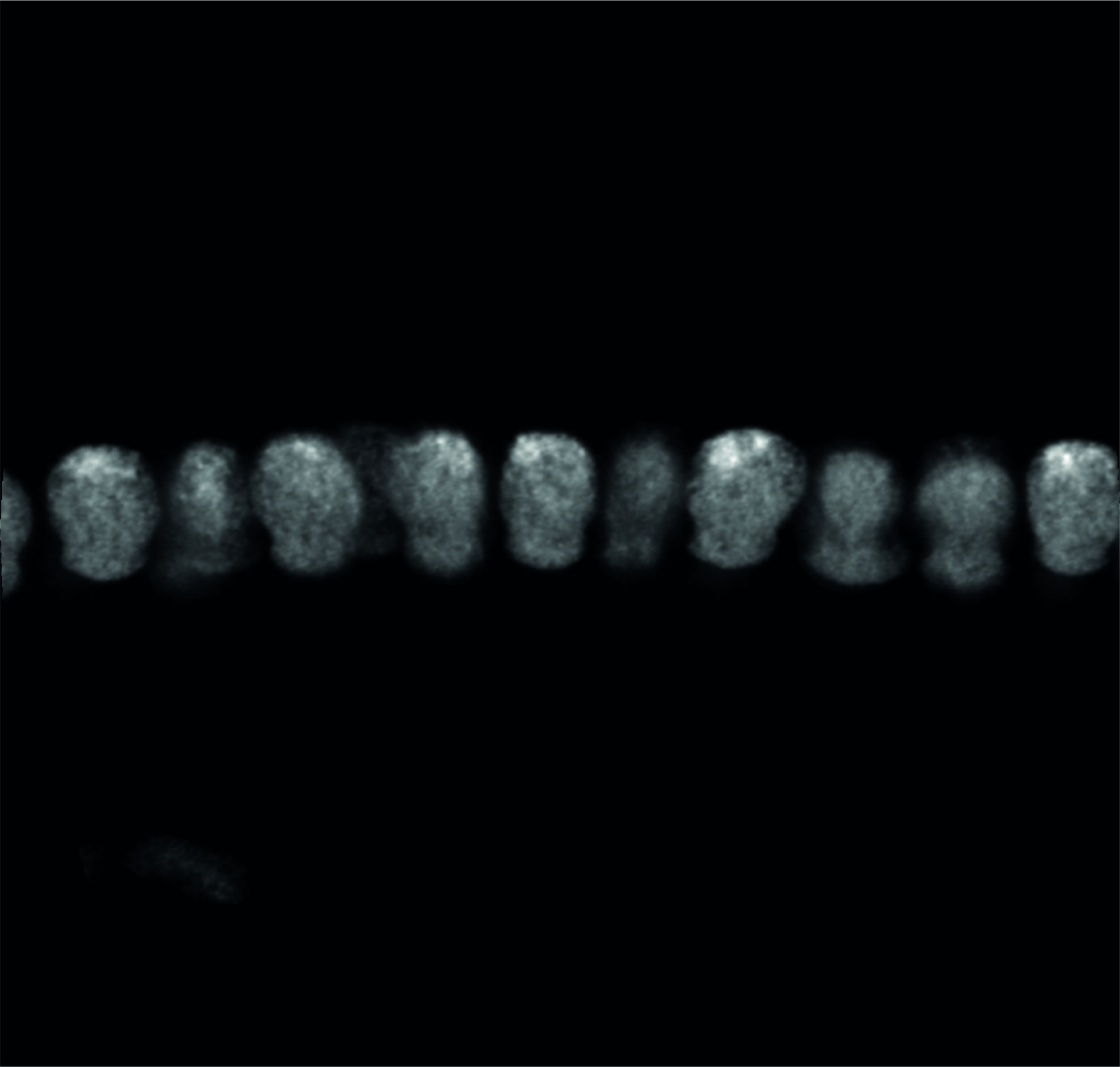

Supplement: Supplementary file 11 — Source data Fig. 4 [file 44318_2024_127_MOESM11_ESM.zip › figure4/figure4c/figure 4c_TM_HP1_cycle14.tif]

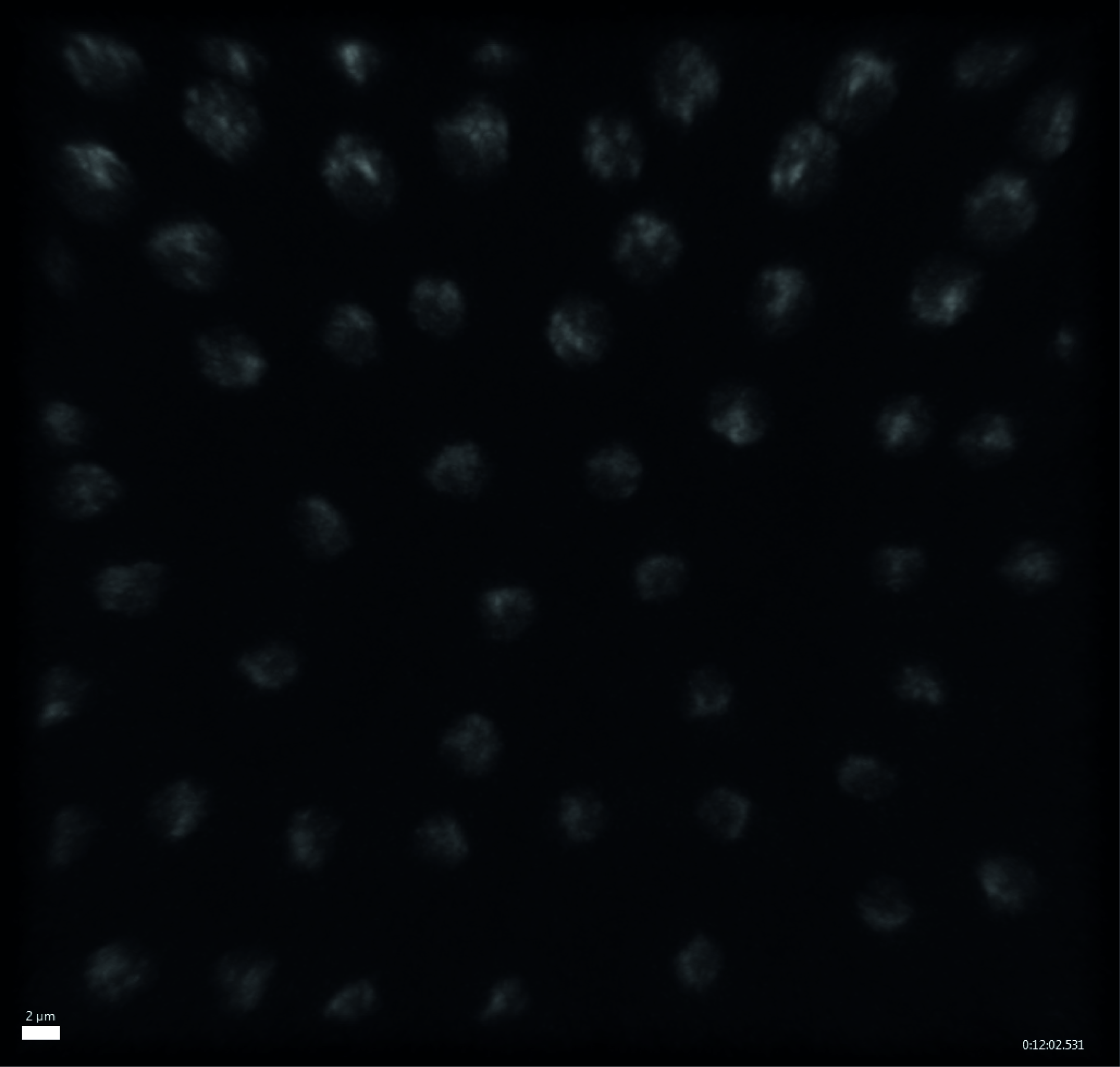

Supplement: Supplementary file 12 — Source data Fig. 5 [file 44318_2024_127_MOESM12_ESM.zip › figure5/figure5b/figure 5b_ctr_0min.tif]

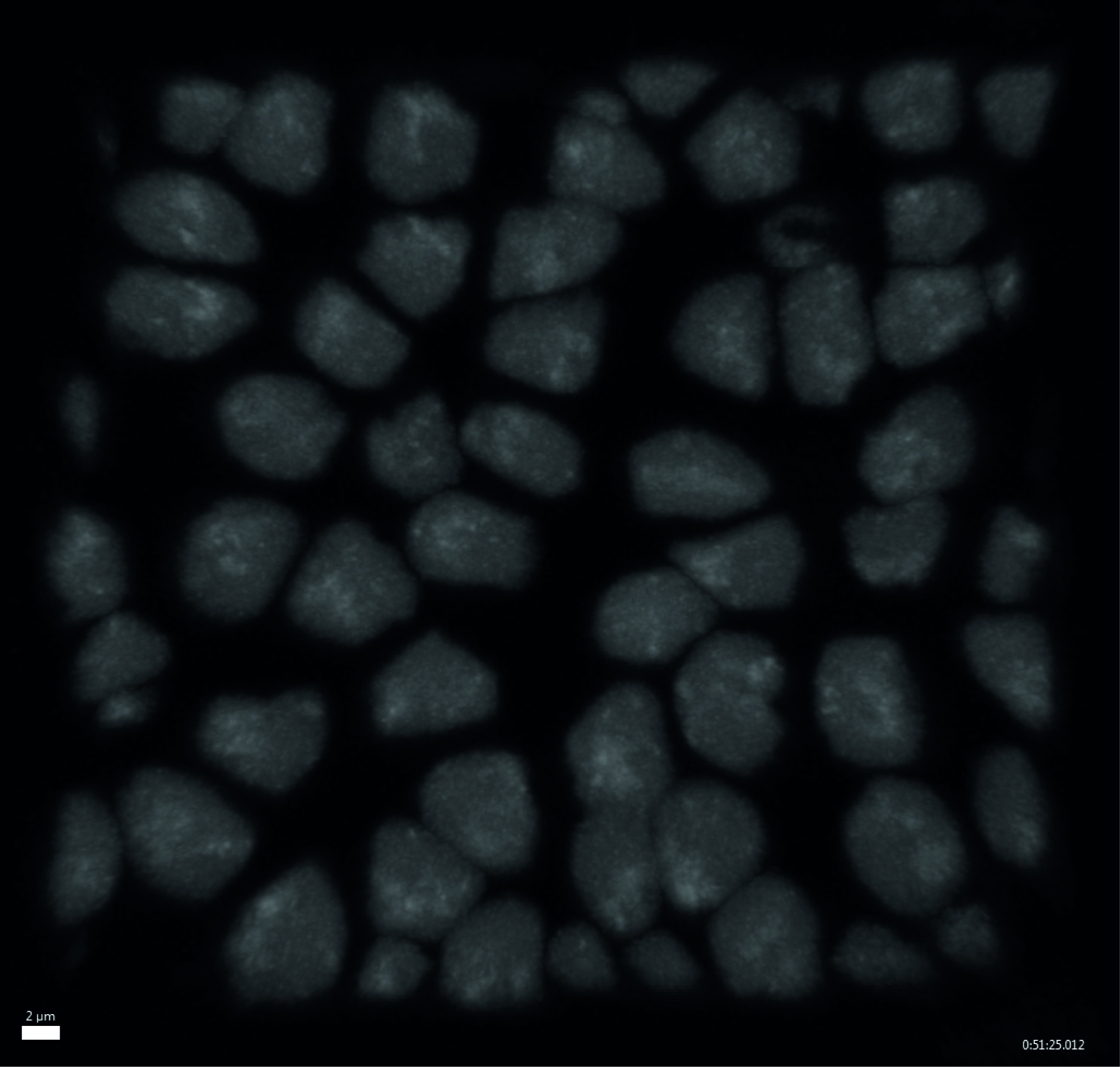

Supplement: Supplementary file 12 — Source data Fig. 5 [file 44318_2024_127_MOESM12_ESM.zip › figure5/figure5b/figure 5b_TM_30min.tif]

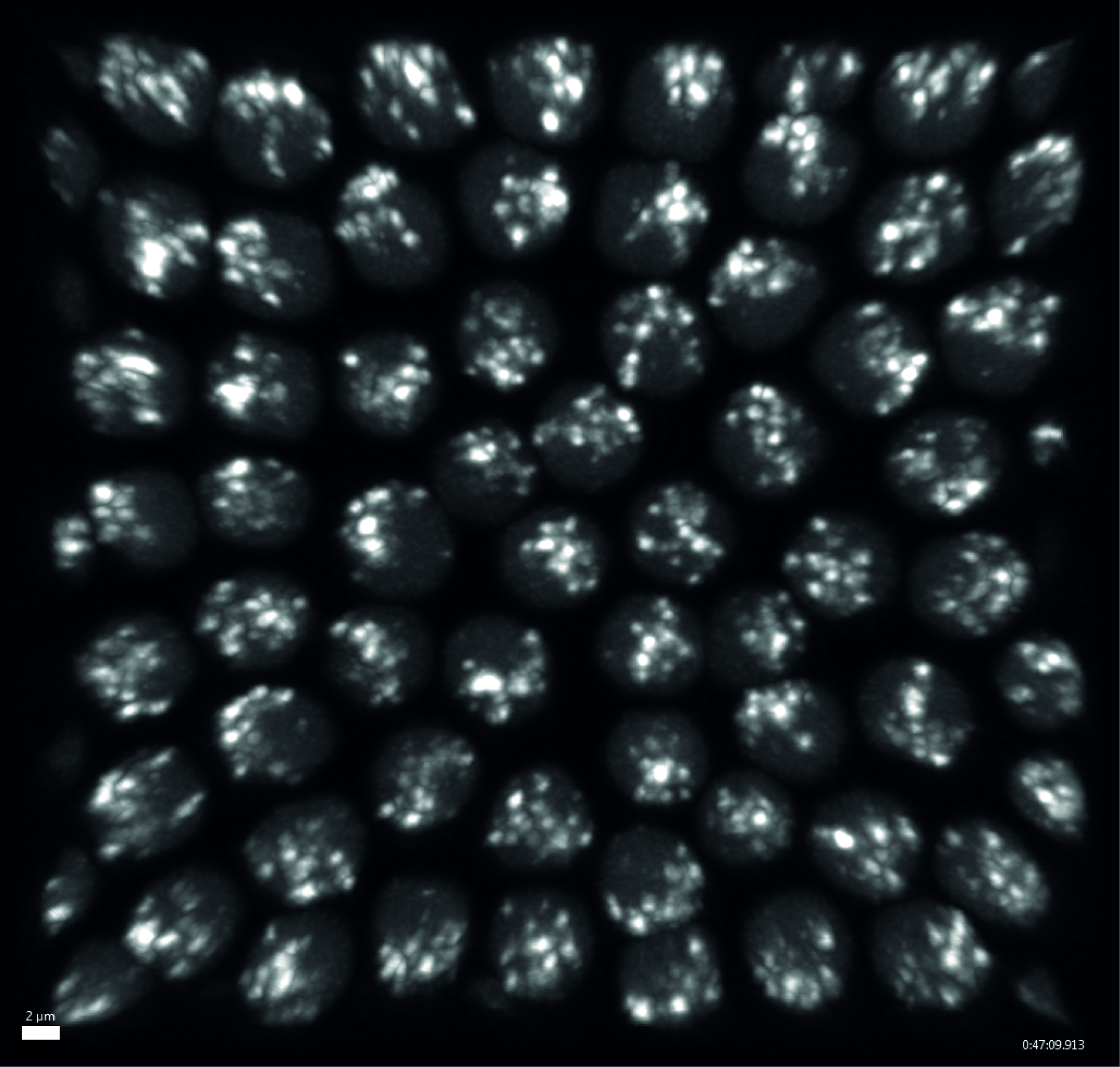

Supplement: Supplementary file 12 — Source data Fig. 5 [file 44318_2024_127_MOESM12_ESM.zip › figure5/figure5b/figure 5b_ctr_35min.tif]

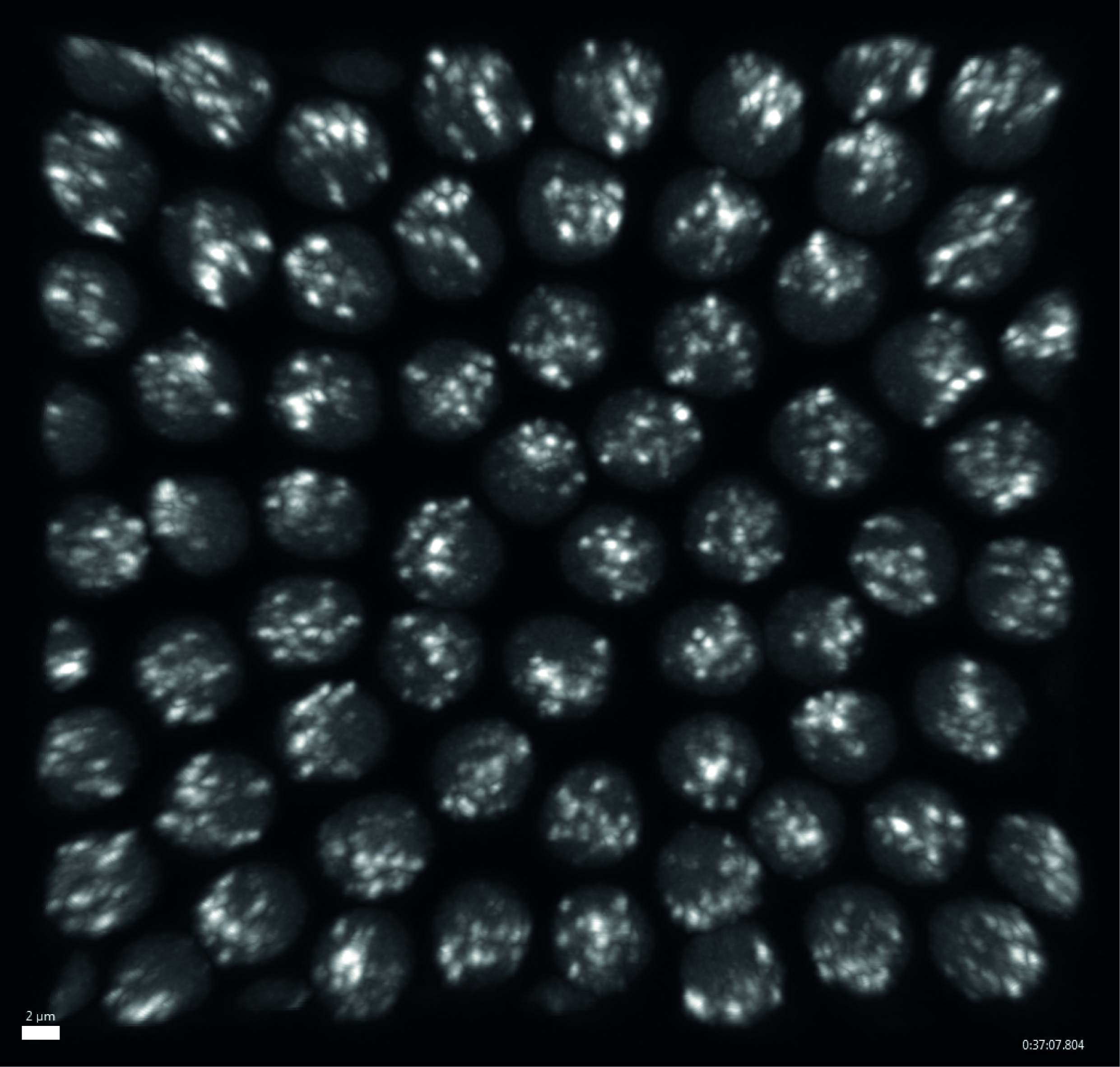

Supplement: Supplementary file 12 — Source data Fig. 5 [file 44318_2024_127_MOESM12_ESM.zip › figure5/figure5b/figure 5b_ctr_25min.tif]

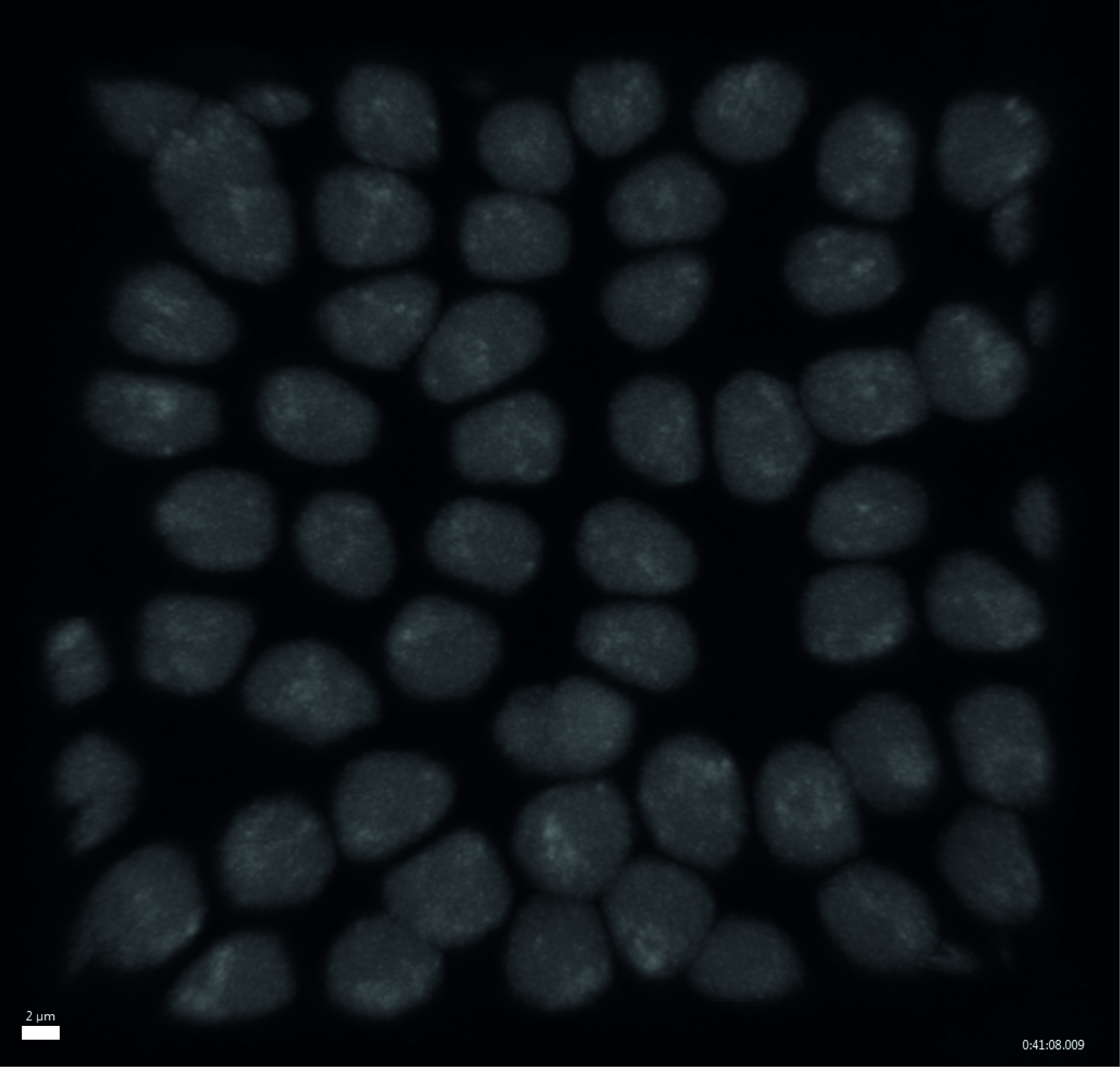

Supplement: Supplementary file 12 — Source data Fig. 5 [file 44318_2024_127_MOESM12_ESM.zip › figure5/figure5b/figure 5b_TM_20min.tif]

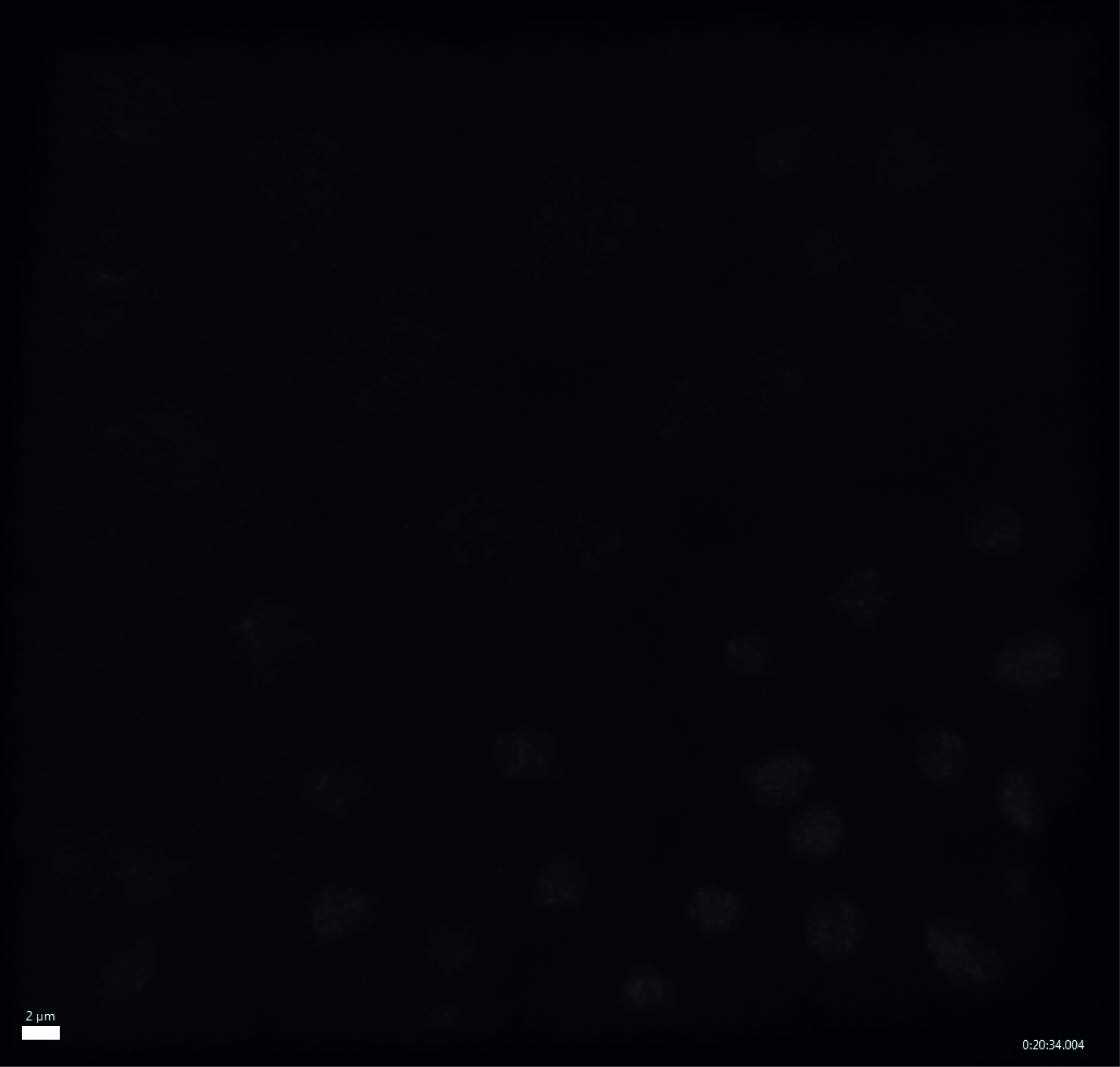

Supplement: Supplementary file 12 — Source data Fig. 5 [file 44318_2024_127_MOESM12_ESM.zip › figure5/figure5b/figure 5b_TM_0min.tif]

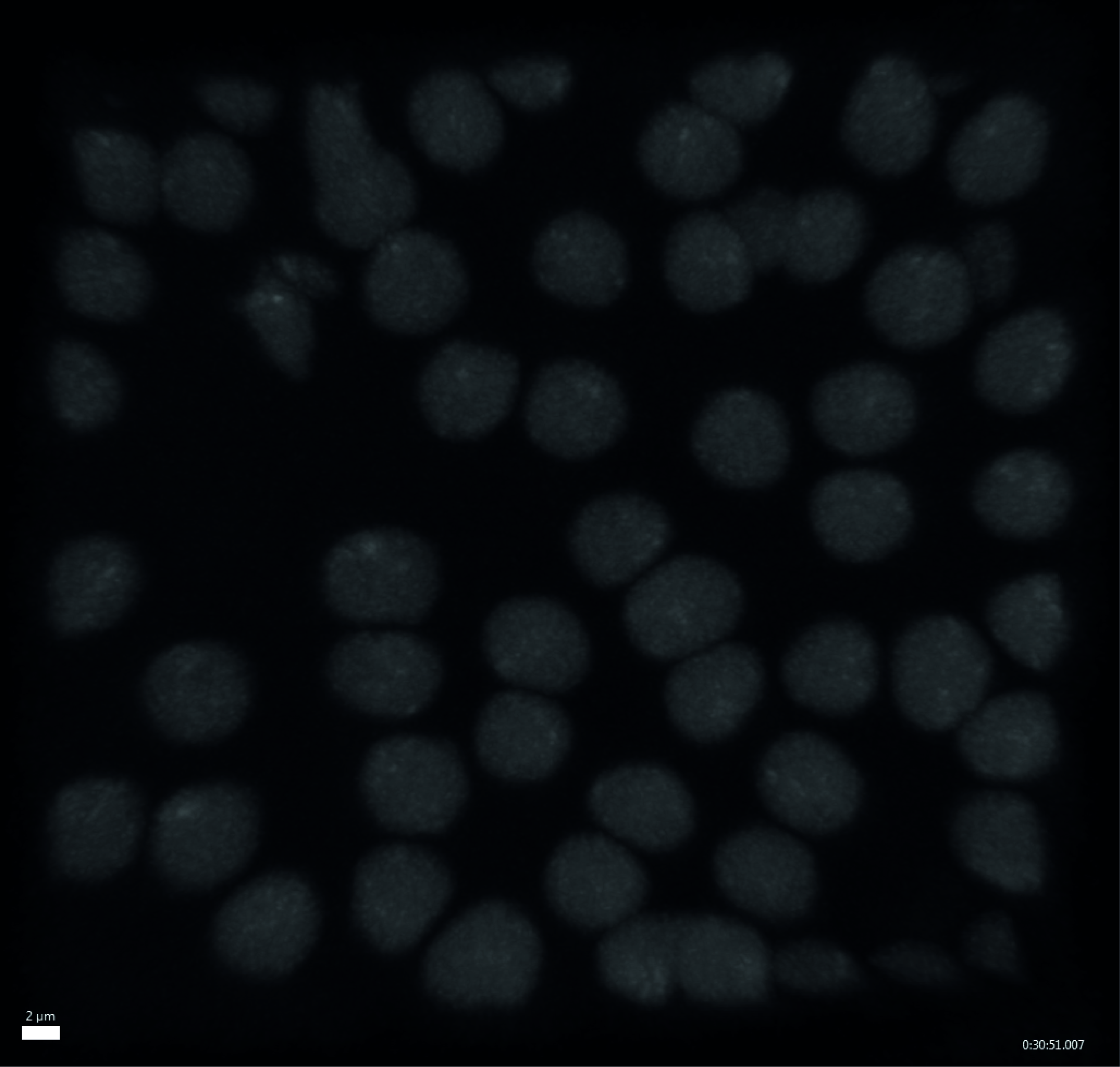

Supplement: Supplementary file 12 — Source data Fig. 5 [file 44318_2024_127_MOESM12_ESM.zip › figure5/figure5b/figure 5b_TM_10min.tif]

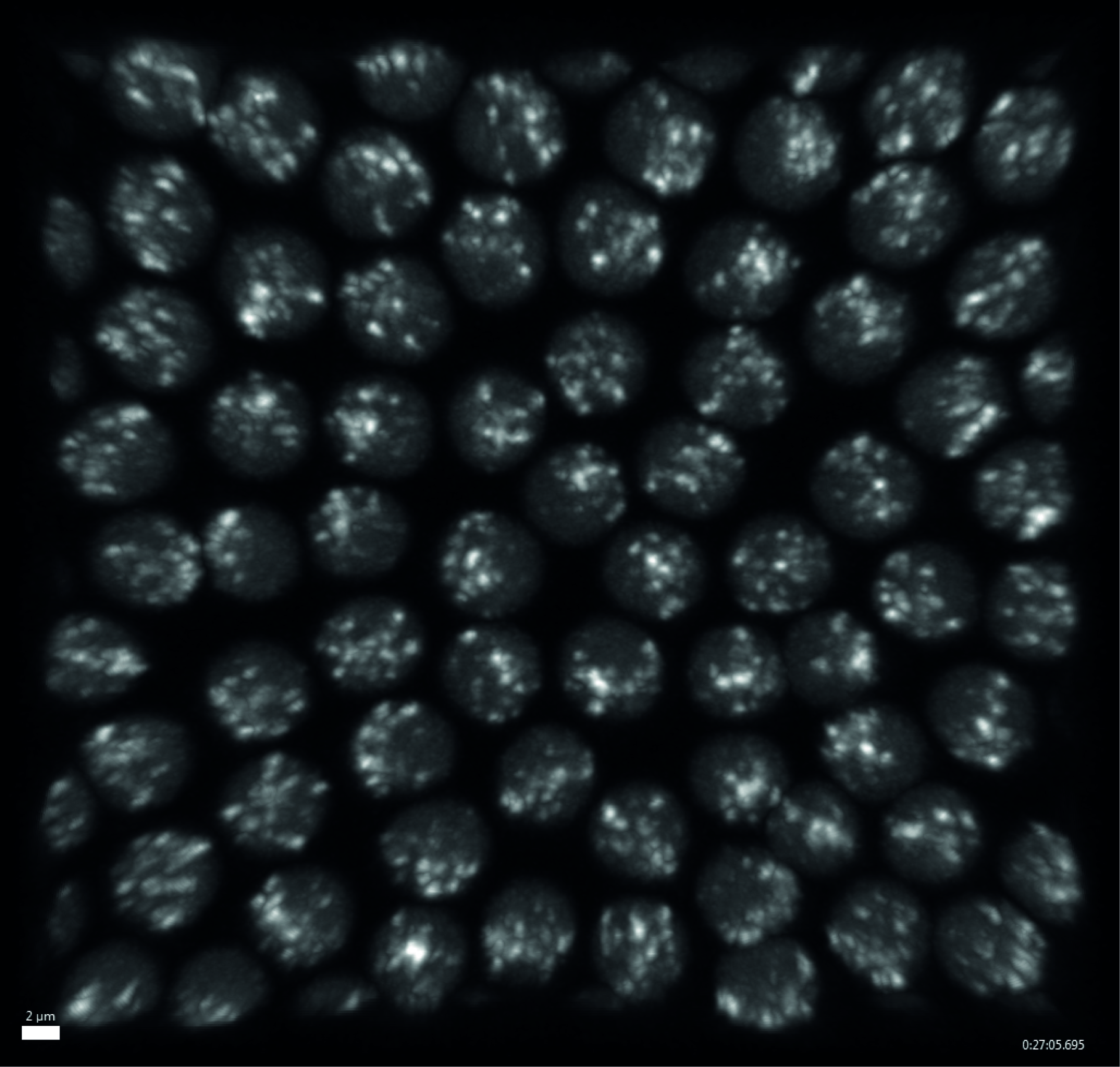

Supplement: Supplementary file 12 — Source data Fig. 5 [file 44318_2024_127_MOESM12_ESM.zip › figure5/figure5b/figure 5b_ctr_15min.tif]

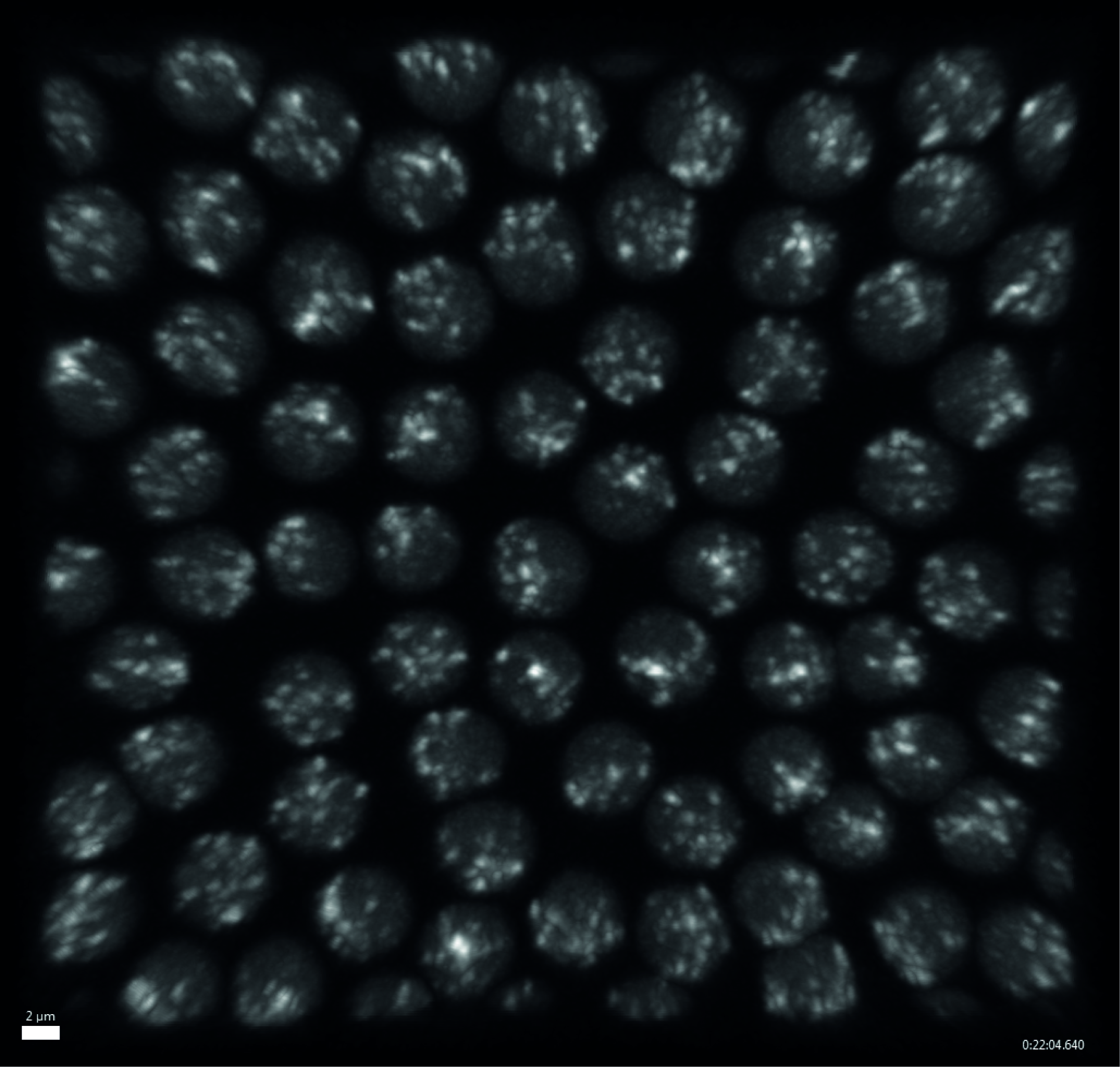

Supplement: Supplementary file 12 — Source data Fig. 5 [file 44318_2024_127_MOESM12_ESM.zip › figure5/figure5b/figure 5b_ctr_10min.tif]

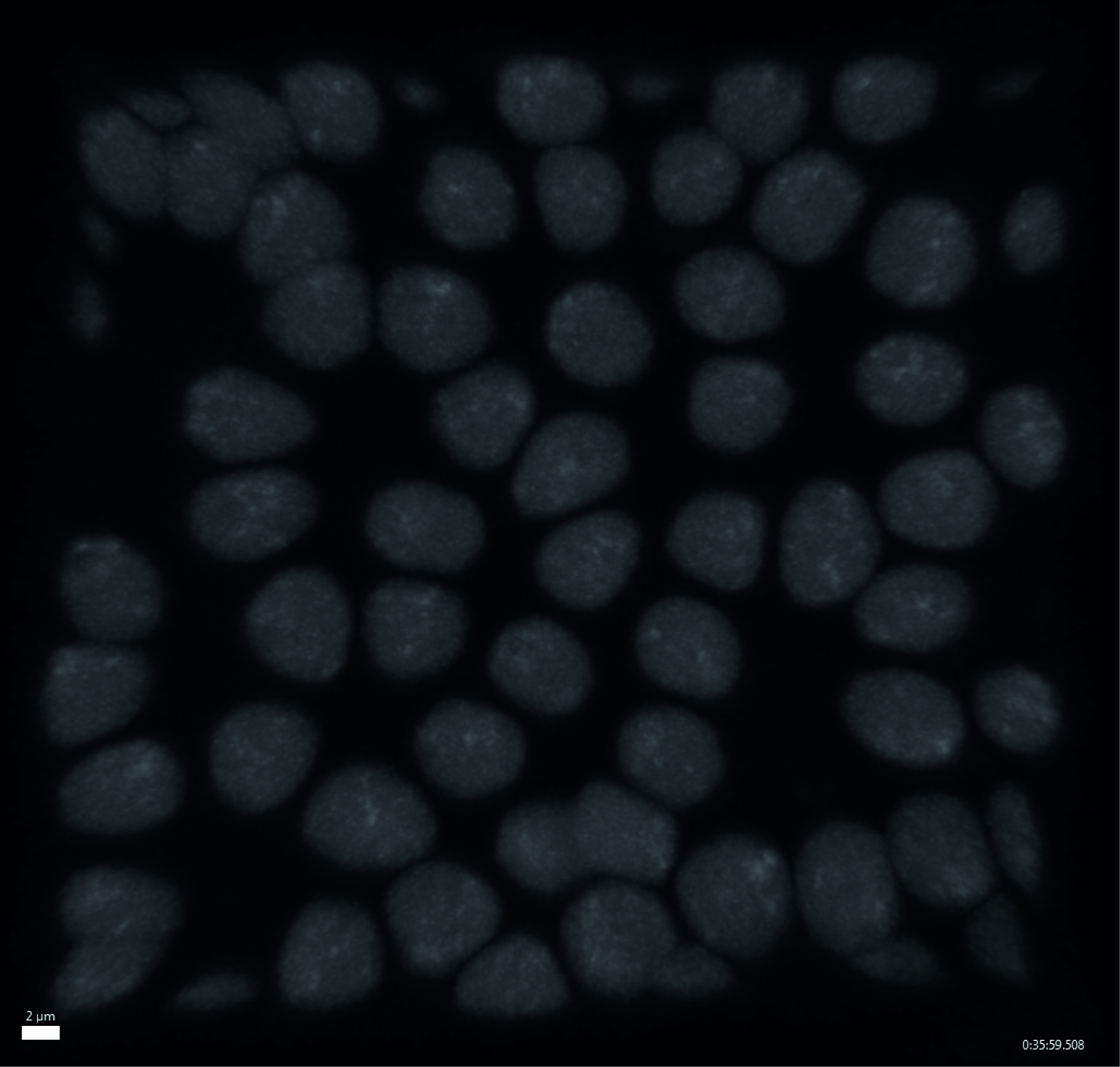

Supplement: Supplementary file 12 — Source data Fig. 5 [file 44318_2024_127_MOESM12_ESM.zip › figure5/figure5b/figure 5b_TM_15min.tif]

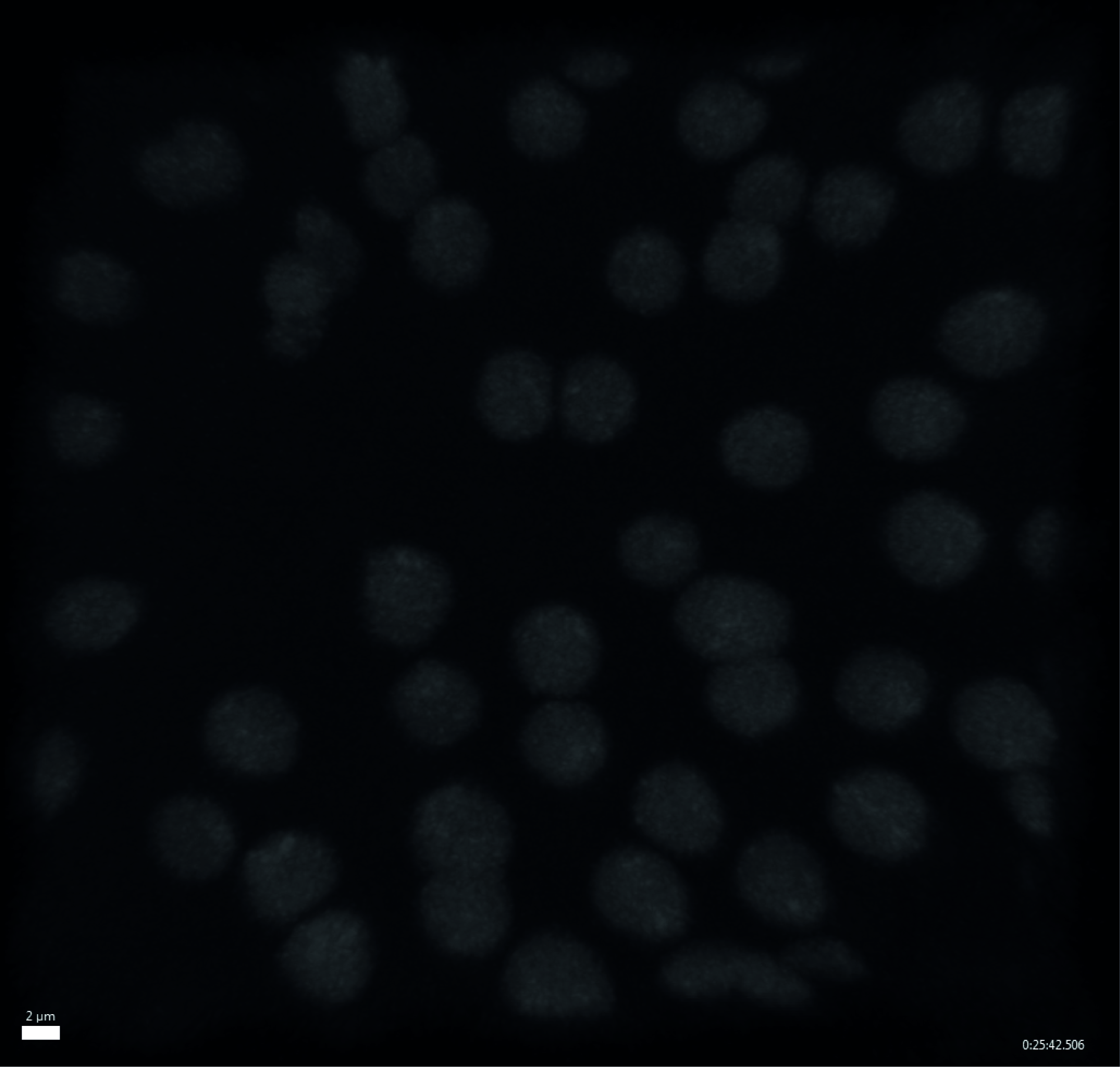

Supplement: Supplementary file 12 — Source data Fig. 5 [file 44318_2024_127_MOESM12_ESM.zip › figure5/figure5b/figure 5b_TM_5min.tif]

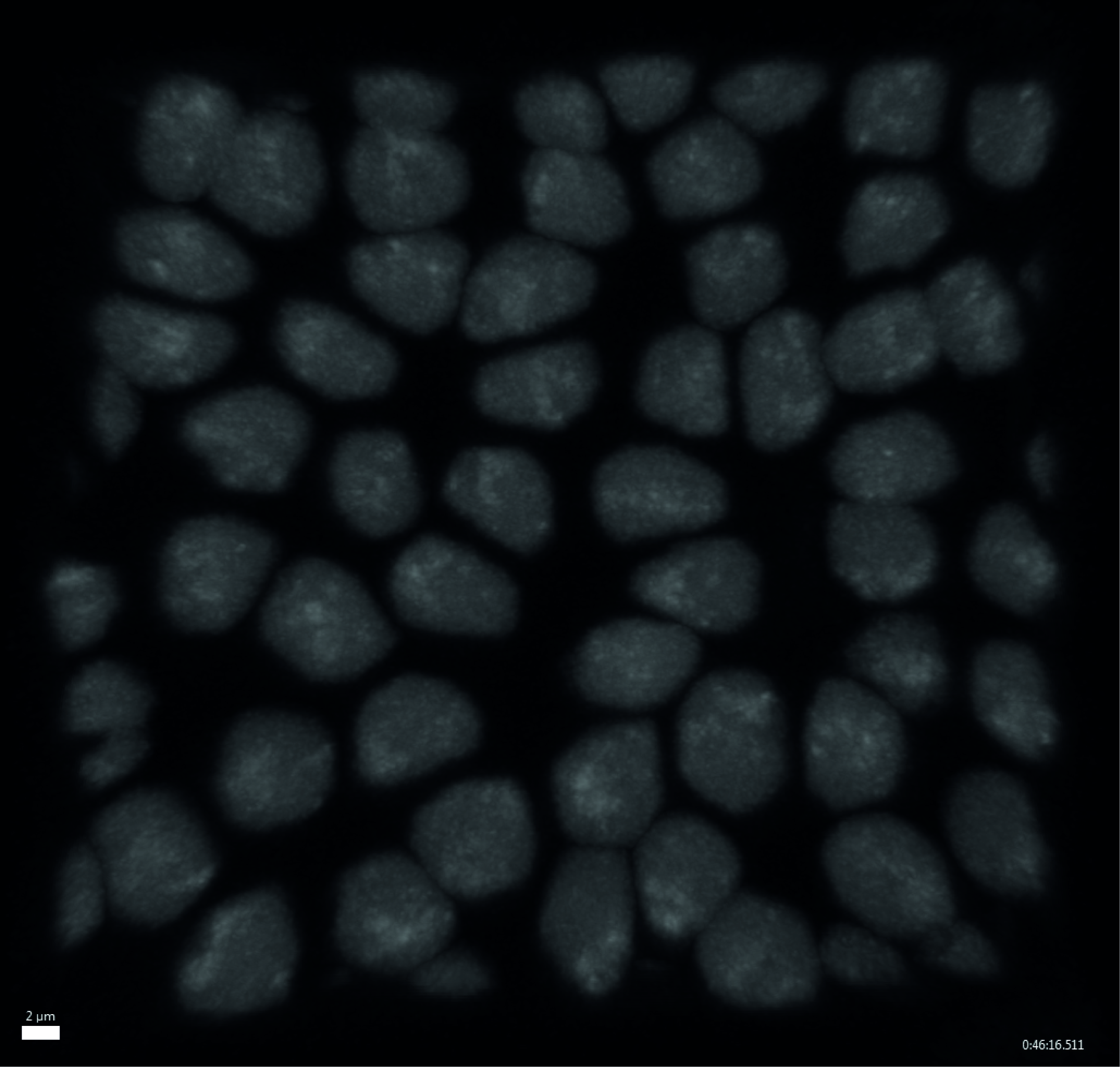

Supplement: Supplementary file 12 — Source data Fig. 5 [file 44318_2024_127_MOESM12_ESM.zip › figure5/figure5b/figure 5b_TM_25min.tif]

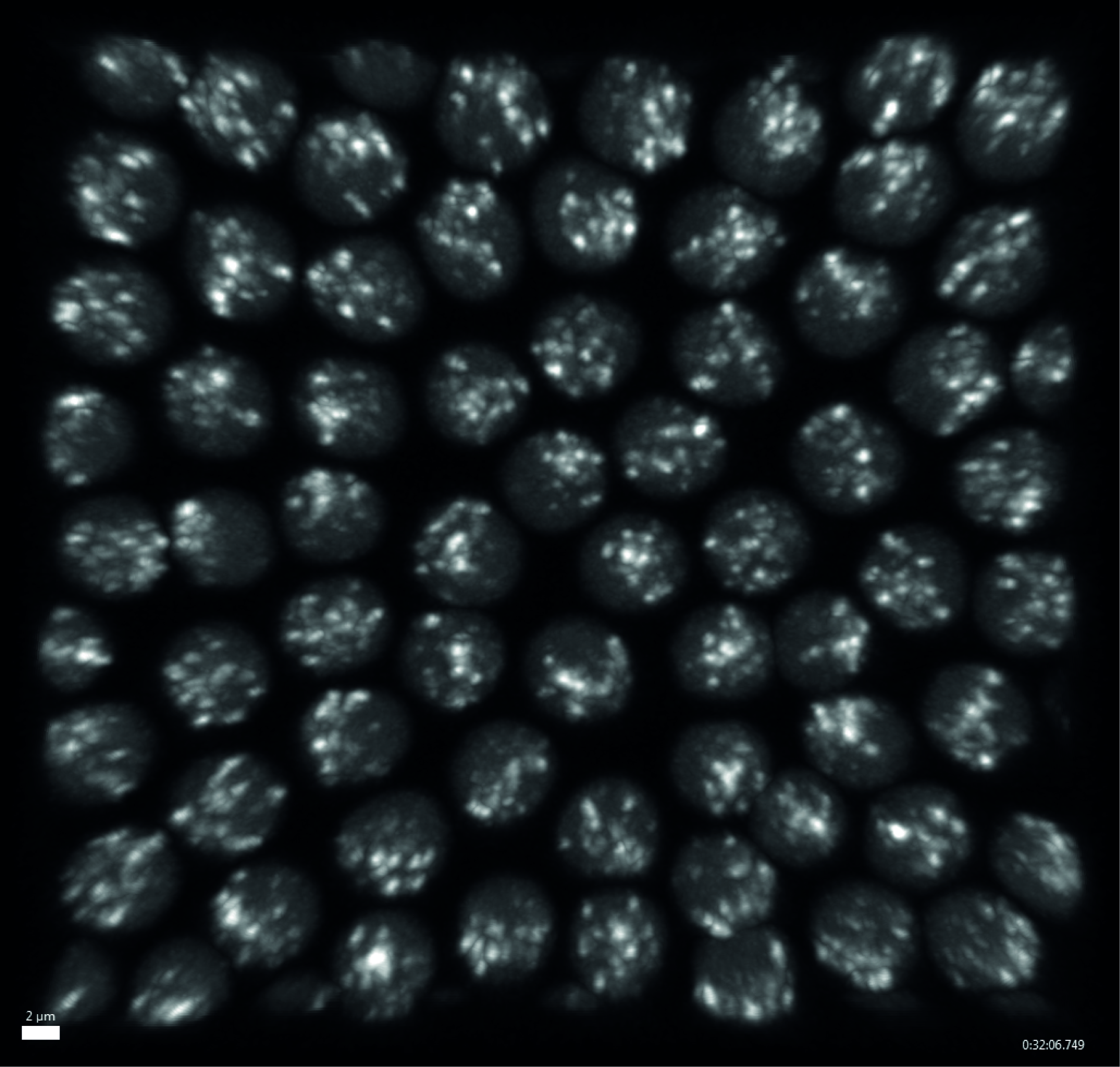

Supplement: Supplementary file 12 — Source data Fig. 5 [file 44318_2024_127_MOESM12_ESM.zip › figure5/figure5b/figure 5b_ctr_20min.tif]

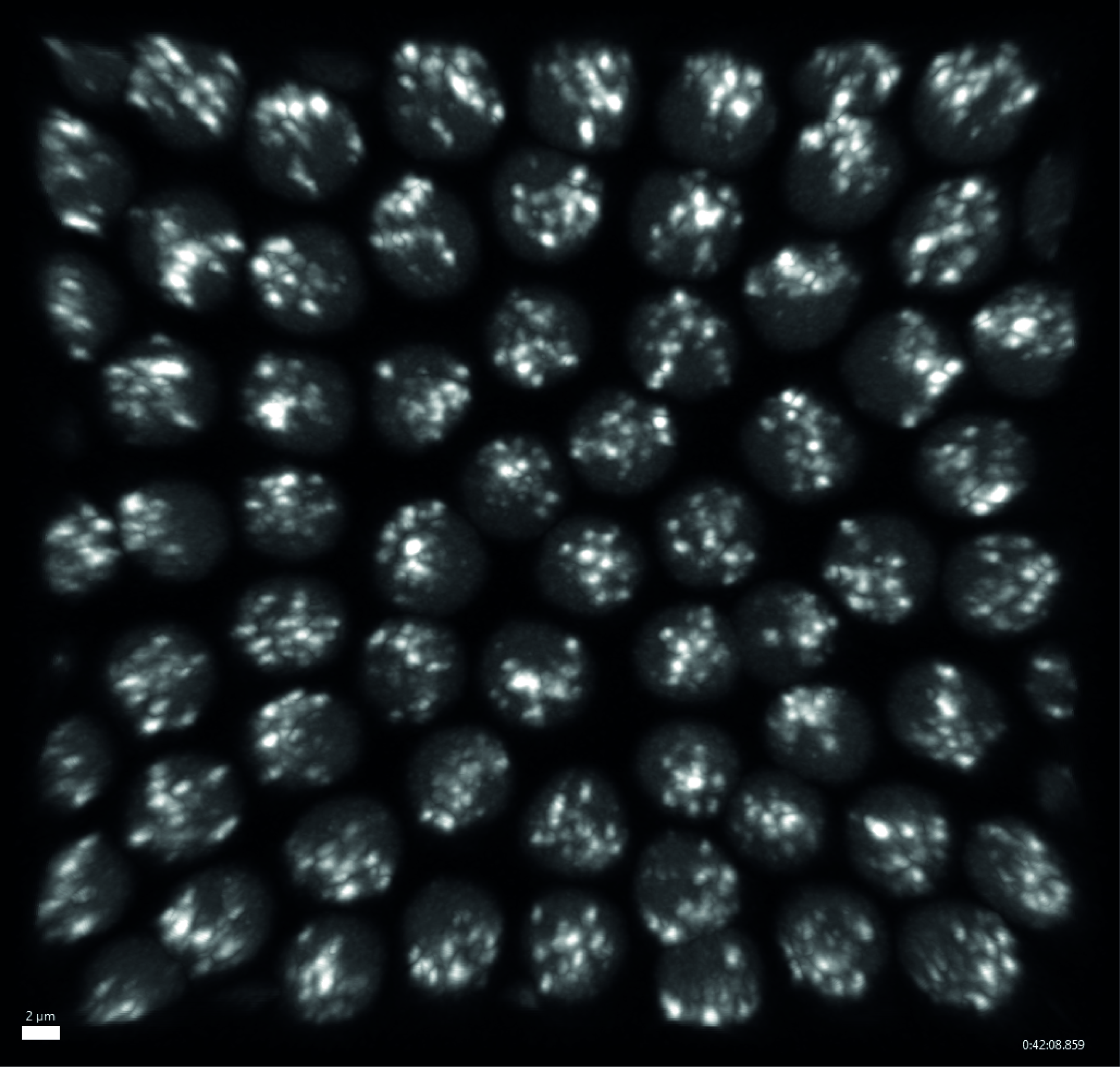

Supplement: Supplementary file 12 — Source data Fig. 5 [file 44318_2024_127_MOESM12_ESM.zip › figure5/figure5b/figure 5b_ctr_30min.tif]

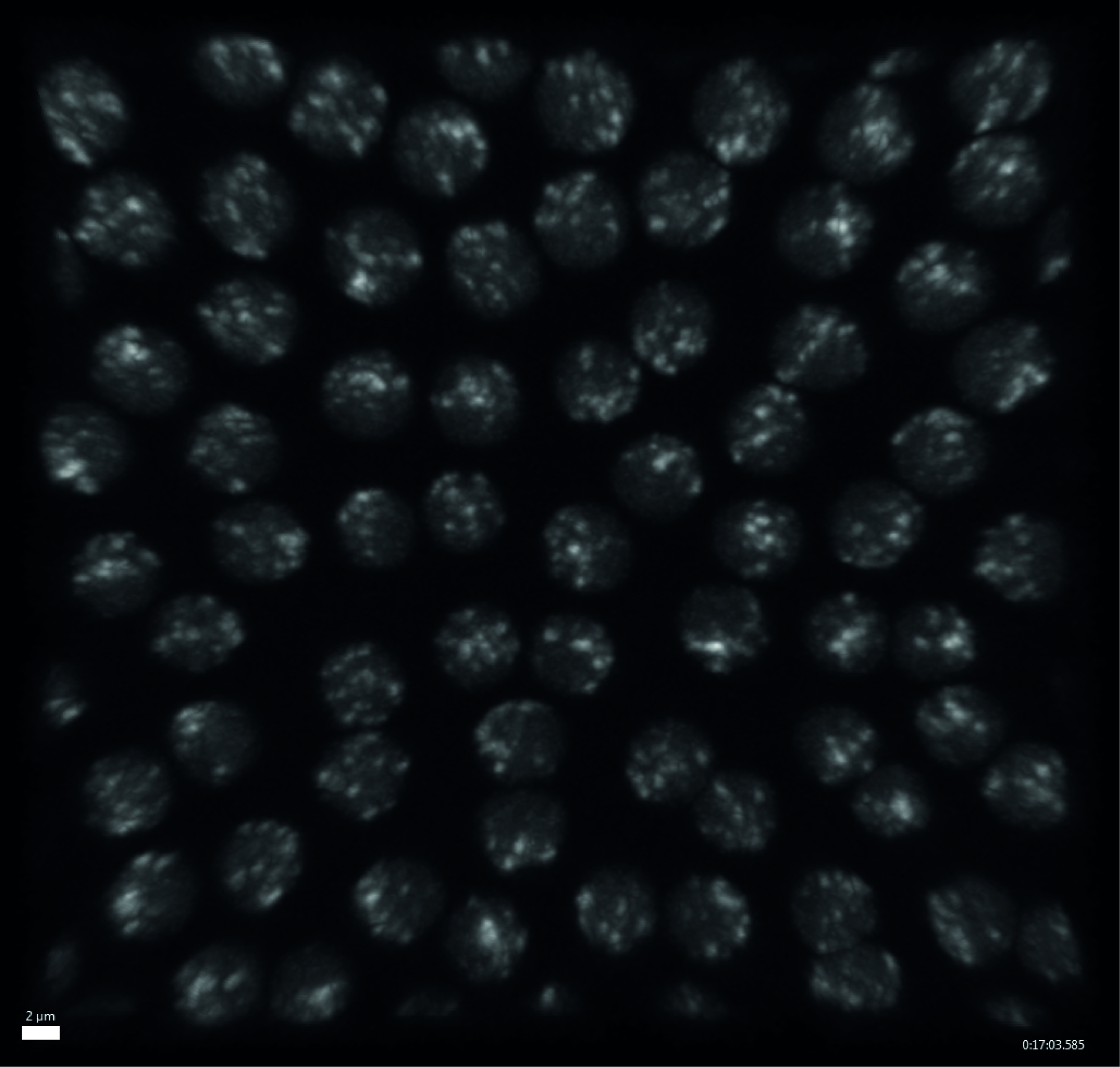

Supplement: Supplementary file 12 — Source data Fig. 5 [file 44318_2024_127_MOESM12_ESM.zip › figure5/figure5b/figure 5b_ctr_5min.tif]

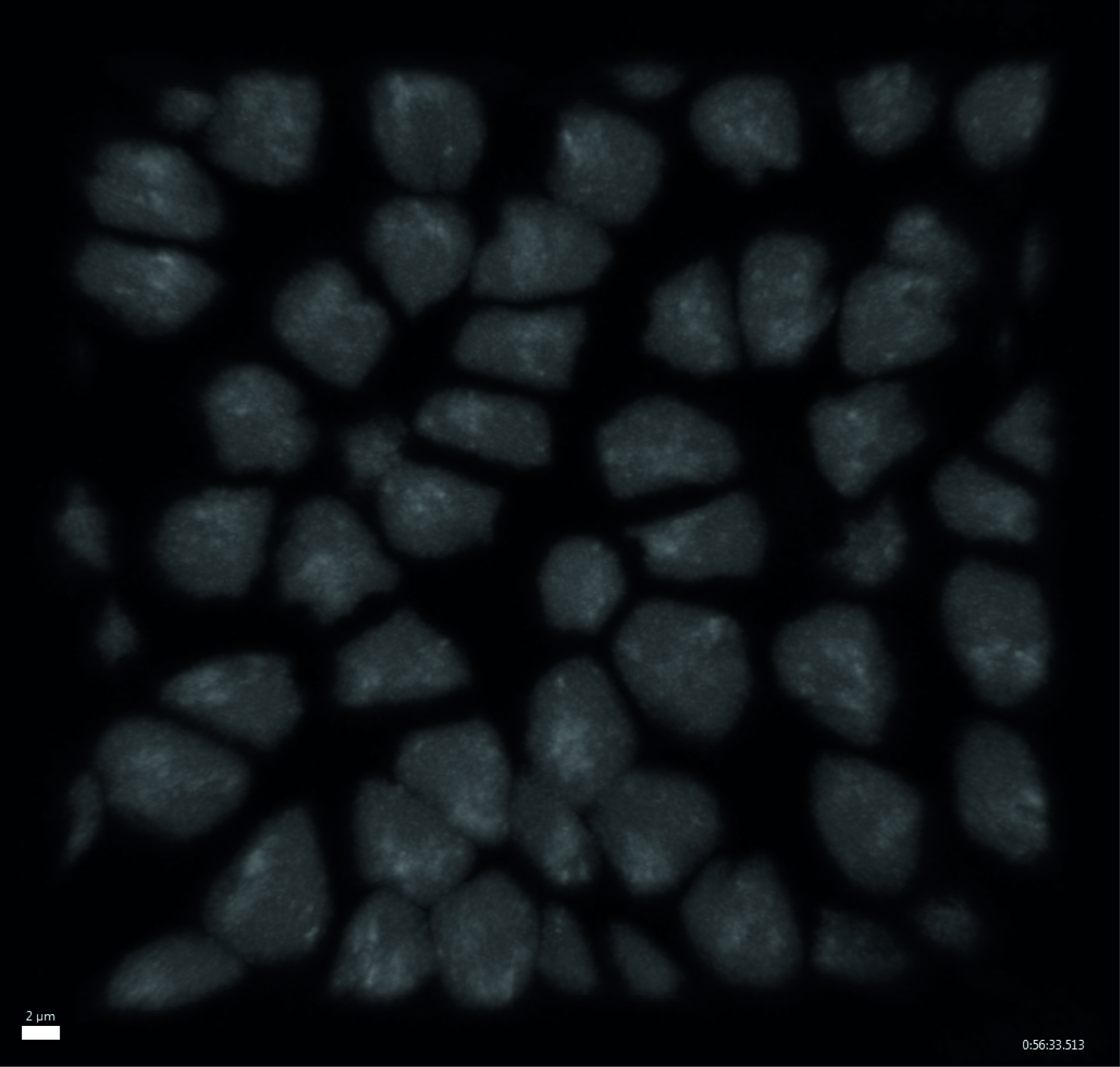

Supplement: Supplementary file 12 — Source data Fig. 5 [file 44318_2024_127_MOESM12_ESM.zip › figure5/figure5b/figure 5b_TM_35min.tif]
