# Supplementary material for: Inheritance of H3K9 methylation regulates genome architecture in Drosophila early embryos
Source: EMBO J. 2024 Jun 3;43(13):8. doi: 10.1038/s44318-024-00127-z (PMC11217351; doi:10.1038/s44318-024-00127-z)
Supplement: Supplementary file 13 — Expanded View Figures [file 44318_2024_127_MOESM13_ESM.pdf]

Expanded View Figures

Figure EV1. H3K9me2/3 signals are specific.

(**A**) Antibody for H3K9me2 and 4‘,6-diaminido-2 phenylindole (DAPI) staining of early embryos at different developmental cycles. For clarity, mitotic chromosomes for some cycles are shown. *n* is 3 embryos per genotype and per stage. Scale bars, 10 µm. (**B**) Heatmaps of H3K9me3 at different developmental stages of early embryogenesis: before cycle 9, cycle 9–13 and ZGA (cycle 14) clustered into pericentromeric regions (PCH) and chromosome arms (chr_arms) of ctr and TM. The last column represents the density of repeats at H3K9me3 ZGA peaks. The signal is ±10 kb centered on H3K9me3 peaks at cycle 14 embryos and ranked by signal intensity at cycle 14. Mean signal of two biological replicates is shown. (**C**) Heatmaps of H3K9me2 at different developmental stages of early embryogenesis: before cycle 9, cycle 9–13 and ZGA (cycle 14) clustered into PCH and chr_arms of ctr and TM. The last column represents the density of repeats at H3K9me3 cycle 14 peaks. The signal is ±10 kb centered on H3K9me2 peaks at cycle 14 embryos and ranked by signal intensity at cycle 14. Mean signal of two biological replicates is shown. (**D**) Quantification of H3K9me3 (left) and H3K9me2 (right) peak number at PCH and chr_arms at before cycle 9, cycle 9–13 and cycle 14 in ctr. (**E**) The fraction of repetitive elements enriched for H3K9me3 peaks at different developmental stages of early embryogenesis: before cycle 9, cycle 9–13 and cycle 14. The fraction was calculated by dividing the number of repeats with H3K9me3 signal at each stage to total number of repeats (~37,480). See also Dataset EV1. (**F**) Boxplots of H3K9me3 (left) and H3K9me2 (right) peak size at PCH and chr_arms at before cycle 9, cycle 9–13 and cycle 14. The boxplot indicates interquartile range from 1st (Q1) and 3rd (Q3) quartile, whiskers denote 1.5 times interquartile region (IQR) below Q1 and above Q3. Dots represent outliers. (**G**) Quantification of nucleotide coverage of H3K9me3 signal (left) and H3K9me2 signal (right) at PCH and chr_arms at before cycle 9, cycle 9–13 and cycle 14 in ctr. (**H**) Antibody for GFP for endogenously tagged dSetDB1 and Su(var)3-9 with GFP, antibody for G9a and DAPI staining of early embryos at different developmental cycles. Insets on the right show a magnification of the signals at cycle 14. n is 3 embryos per genotype and per stage. Scale bars, 10 µm.

© The Author(s)

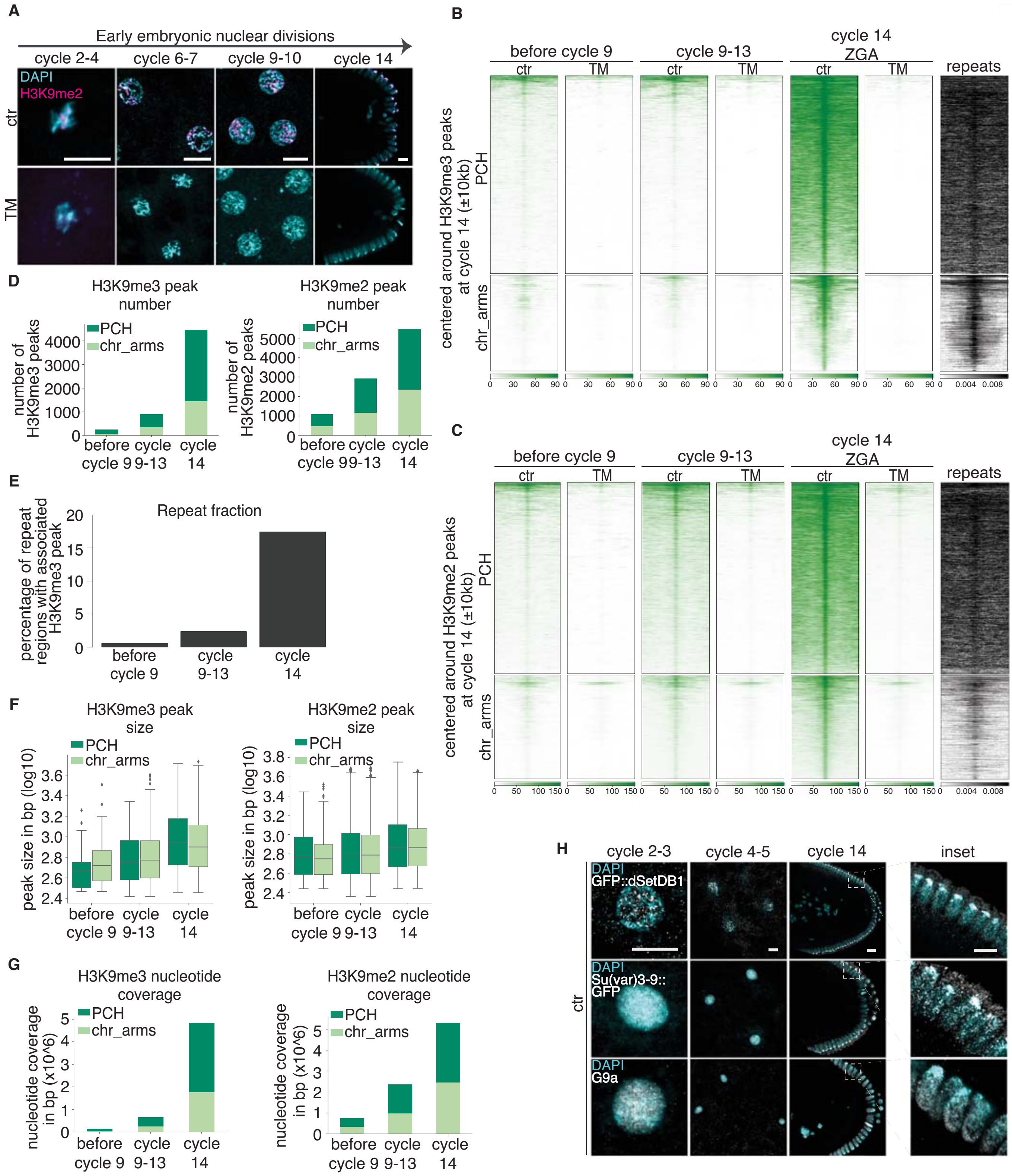
A
Early embryonic nuclear divisions
cycle 2-4
cycle 6-7
cycle 9-10
cycle 14
DAPI
H3K9me2
ctr
TM
B
before cycle 9
cycle 9-13
cycle 14 ZGA
ctr
TM
repeats
centered around H3K9me3 peaks at cycle 14 (±10kb)
PCH
chr_arms
C
centered around H3K9me2 peaks at cycle 14 (±10kb)
D
H3K9me3 peak number
H3K9me2 peak number
number of H3K9me3 peaks
number of H3K9me2 peaks
PCH
chr_arms
before cycle 9
cycle 9-13
cycle 14
E
Repeat fraction
percentage of repeat regions with associated H3K9me3 peak
F
H3K9me3 peak size
H3K9me2 peak size
peak size in bp (log10)
G
H3K9me3 nucleotide coverage
H3K9me2 nucleotide coverage
nucleotide coverage in bp (x10^6)
H
cycle 2-3
cycle 4-5
cycle 14
inset
DAPI
GFP::dSetDB1
Su(var)3-9::GFP
G9a

 © The Author(s)

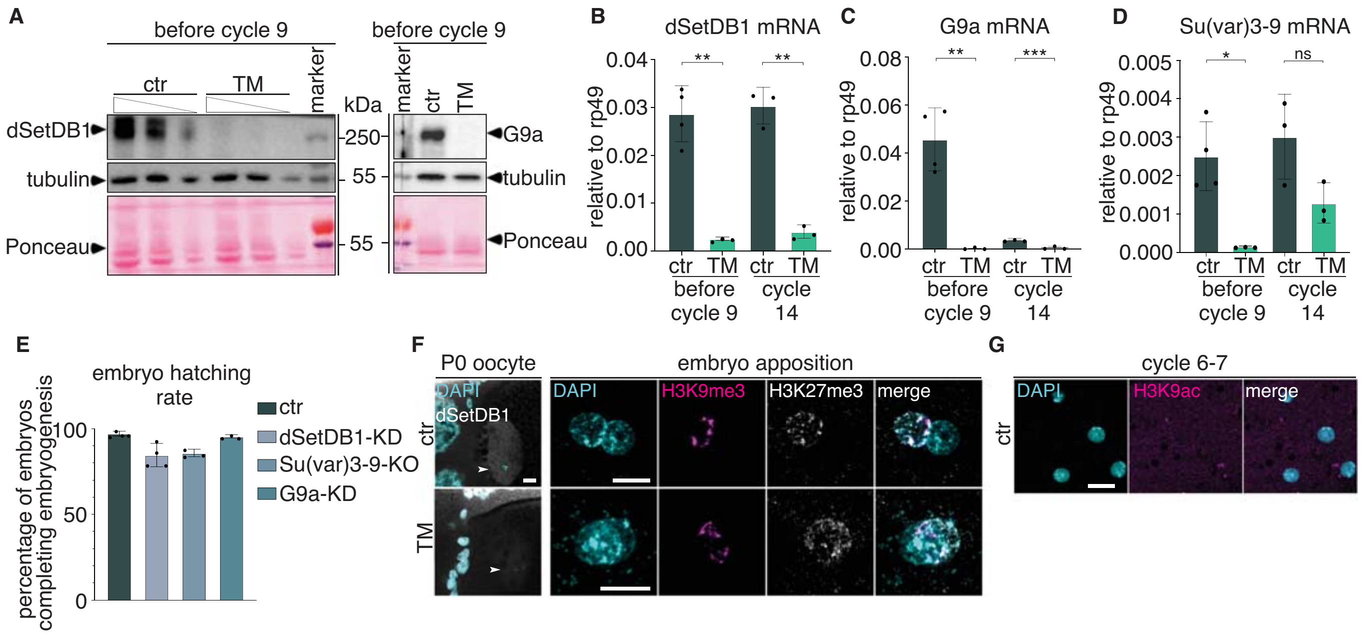

Figure EV2. TM is devoid of all H3K9 HMTs.

(**A**) Left panel: western blot for dSetDB1 on different amount of protein extract from ctr and TM before cycle-9 embryos. Before cycle-9 embryos represent the amount of maternally loaded proteins. Right panel: western blot for G9a on protein extract from ctr and TM before cycle-9 embryos. Tubulin and Ponceau staining are used as loading controls. See also Dataset EV2. (**B**) dSetDB1 mRNA levels relative to rp49 in ctr and TM before cycle-9 and cycle 14 embryos measured by RT-qPCR. Before cycle-9 embryos represent the amount of maternally loaded mRNAs. $n = 3$–4 biological replicates were used for each genotype at each time point (for before cycle-9 embryo, P value = 0.0029, two-tailed Welch two-sample t test, for cycle 14 embryo, P value = 0.0033, two-tailed Welch two-sample t test). Each dot represents one biological replicate. Shown mean +/− s.d. (**C**) G9a mRNA levels relative to rp49 in ctr and TM before cycle-9 and cycle 14 embryos measured by RT-qPCR. $n = 3$–4 biological replicates were used for each genotype at each time point (for before cycle-9 embryo, P value = 0.0061, two-tailed Welch two-sample t test, for cycle 14 embryo, P value = 0.0003, two-tailed Welch two-sample t test). Each dot represents one biological replicate. Shown mean +/− s.d. (**D**) Su(var)3-9 mRNA levels relative to rp49 in ctr and TM before cycle-9 and cycle 14 embryos measured by RT-qPCR. $n = 3$–4 biological replicates are used for each genotype at each time point (for before cycle-9 embryo, P value = 0.0136, two-tailed Welch two-sample t test, for cycle 14 embryo, P value = 0.0963, two-tailed Welch two-sample t test). Each dot represents one biological replicate. Shown mean +/− s.d. (**E**) Hatching rate of ctr and single mutants: G9a-KO, Su(var)3-9-KO and dSetDB1-KD embryos. $n = 4$ biological replicates with total 480 embryos were used for ctr and dSetDB1-KD and $n = 3$ biological replicates with total 360 embryos were used for Su(var)3-9-KO and G9a-KO. Each dot represents one biological replicate. Shown mean +/- s.d. (**F**) Left panel: dSetDB1 and DAPI staining of ctr and TM stage 10 egg chamber oocyte. The arrowhead indicates the oocyte. Right panel: H3K9me3, H3K27me3 and DAPI staining of ctr and TM pronuclei at apposition in the zygote. To define the maternal pronucleus H3K27me3 was used as it stains the maternal pronucleus (Zenk et al, 2017). n is at least three embryos per genotype and per stage. Scale bars, 10 μm. (**G**) H3K9ac and DAPI staining of ctr cycle 6–7 embryos. n is at least 2 embryos. Scale bars, 10 μm.

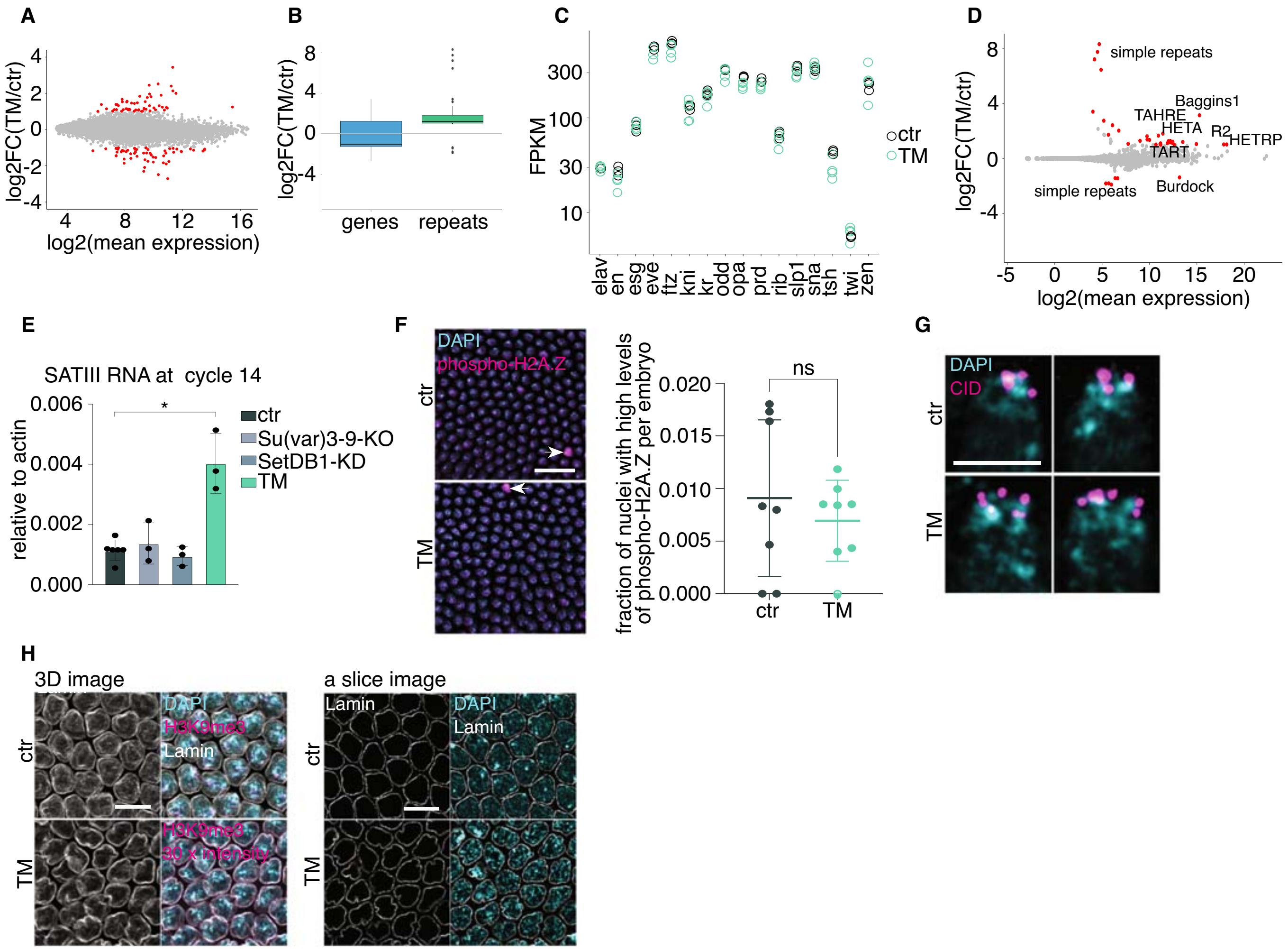

Figure EV3. Absence of H3K9me2/3 does not affect gene expression significantly, but some repeats.

(**A**) MA plot of differential gene expression in TM versus ctr cycle 14 embryos determined by Gro-seq. $n = 3$ biological replicates for ctr and $n = 4$ biological replicates for TM were used for quantification. See also Dataset EV3. (**B**) Boxplots showing the distribution of expression changes for genes and repeats in TM versus ctr cycle 14 embryos. The boxplot indicates interquartile range from 1st (Q1) and 3rd (Q3) quartile, whiskers denote 1.5 times interquartile region (IQR) below Q1 and above Q3. Dots represent outliers. See also Dataset EV3. (**C**) Normalized counts of 16 pure zygotic genes in TM versus ctr cycle 14 embryos similar to (Zenk et al, 2021) obtained by Gro-seq. See also Dataset EV3. (**D**) MA plot of differential repeat expression in TM versus ctr cycle 14 embryos from Gro-seq data. See also Dataset EV3. (**E**) SATIII mRNA levels relative to actin in ctr, Su(var)3-9-KO, dSetDB1-KD and TM cycle 14 embryos measured by RT-qPCR (P value = 0.0314, two-tailed Welch two-sample t test). $n = 6$ biological replicates for ctr and $n = 3$ biological replicates were used for the rest. Each dot represents one biological replicate. Shown mean +/− s.d. (**F**) Left panel: antibody for phosphorylated H2A.Z (phospho-H2A.Z) and DAPI staining of cycle 14 ctr and TM embryos. An arrow represents a nucleus with high levels of phospho-H2A.Z. Scale bars, 20 μm. Right panel: quantification of fraction of nuclei with high phospho-H2A.Z staining normalized by total number of nuclei per embryo in ctr and TM cycle 14 embryos (P value = 0.4856, two-tailed Welch two-sample t test). $n = 8$ embryos were used for quantification for each genotype. (**G**) Representative images of CID and DAPI staining of cycle 14 ctr and TM embryos. Scale bars, 5 μm. (**H**) Representative images of Lamin, H3K9me3 and DAPI staining of cycle 14 ctr and TM embryos. H3K9me3 signal intensity in TM embryos was shown 30 times more than H3K9me3 signal intensity of ctr embryos to see the leftover H3K9me3 signal. Left panel: 3D image of assembled Z-stacks. Right panel: a single slice image. $n = 2$ biological replicates with 9 embryos were used for quantification for each genotype. Scale bars, 10 μm.

 © The Author(s)

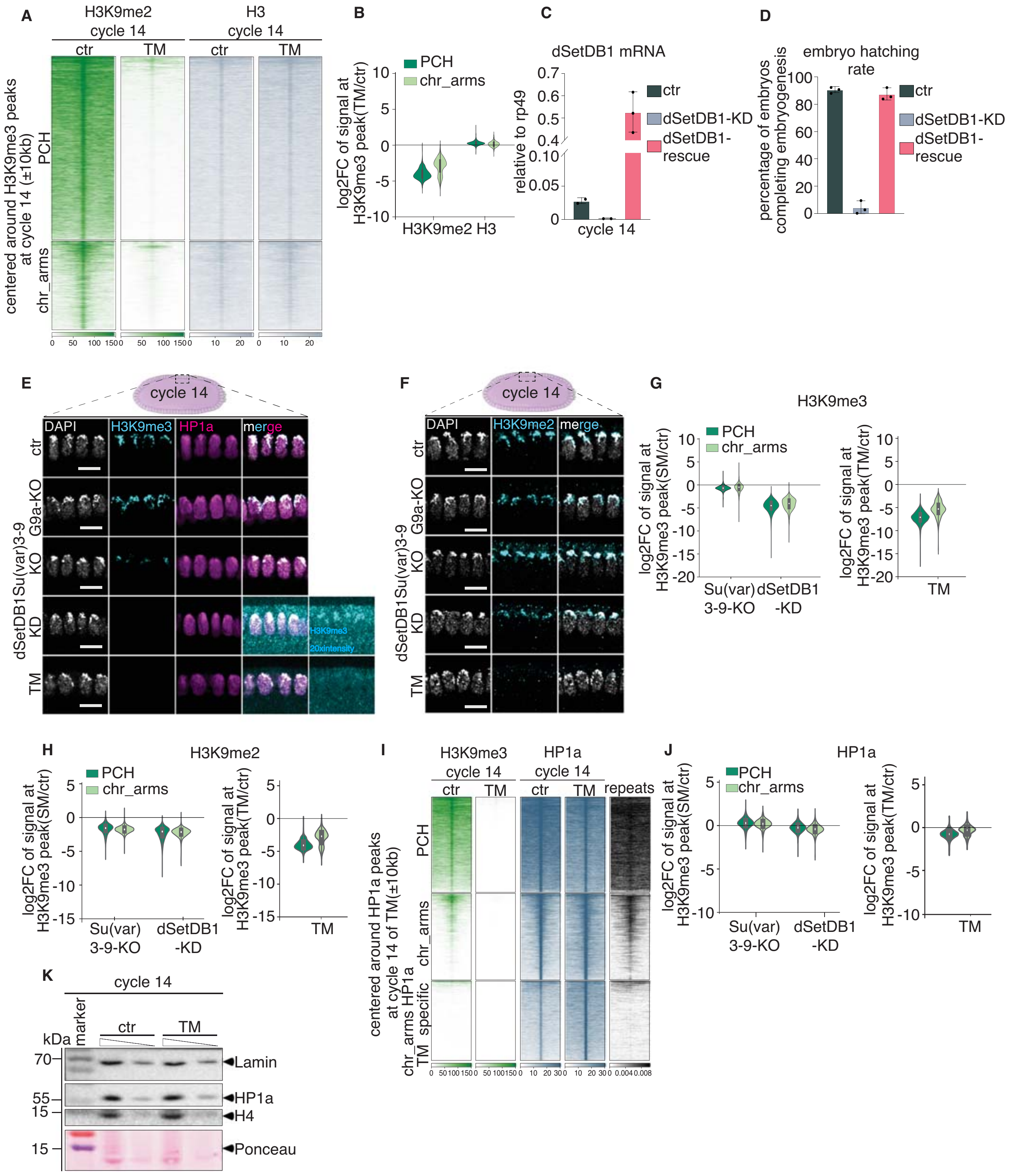
A
H3K9me2 cycle 14
H3 cycle 14
ctr
TM
centered around H3K9me3 peaks at cycle 14 (±10kb)
PCH
chr_arms
0 50 100 150
0 10 20
B
PCH
chr_arms
log2FC of signal at H3K9me3 peak(TM/ctr)
H3K9me2 H3
C
dSetDB1 mRNA
relative to rp49
cycle 14
ctr
dSetDB1-KD
dSetDB1-rescue
D
embryo hatching rate
percentage of embryos completing embryogenesis
E
cycle 14
DAPI
H3K9me3
HP1a
merge
ctr
G9a-KO
Su(var)3-9 KO
dSetDB1 KD
TM
H3K9me3 20xintensity
F
H3K9me2
G
H3K9me3
log2FC of signal at H3K9me3 peak(SM/ctr)
log2FC of signal at H3K9me3 peak(TM/ctr)
Su(var) 3-9-KO
dSetDB1 -KD
H
H3K9me2
I
H3K9me3 cycle 14
HP1a cycle 14
repeats
centered around HP1a peaks at cycle 14 of TM(±10kb)
chr_arms
TM specific chr_arms HP1a
J
HP1a
K
cycle 14
kDa
marker
Lamin
HP1a
H4
Ponceau

Figure EV4. H3K9me2/3 signals are absent only in triple-mutant embryos.

(**A**) Heatmaps of H3K9me2 and H3 at ZGA (cycle 14) clustered into PCH and chr_arms. The signal is ±10 kb centered on H3K9me3 peaks at cycle 14 ctr embryos and ranked by signal intensity at cycle 14 ctr. Mean signal of two biological replicates is shown. (**B**) Violin plot of log2 fold change of H3K9me2 and H3 signal on H3K9me3 peaks found in PCH and chr_arms regions in TM cycle 14 embryos compared to ctr embryos. The boxplot inside indicates interquartile range from 1st (Q1) and 3rd (Q3) quartile, whiskers denote 1.5 times interquartile region (IQR) below Q1 and above Q3. (**C**) dSetDB1 mRNA levels relative to rp49 in ctr, dSetDB1-KD and dSetDB1 rescue cycle 14 embryos measured by RT-qPCR. $n = 3$ biological replicates were used for each genotype. In (**C**, **D**) early oogenesis maternal tubulin driver (see "Methods") was used. Each dot represents one biological replicate. Shown mean $+/-$ s.d. (**D**) Hatching rate of ctr, dSetDB1-KD and dSetDB1 rescue embryos. $n = 3$ biological replicates with total 360 embryos were used for each genotype. Each dot represents one biological replicate. Shown mean $+/-$ s.d. (**E**) H3K9me3, HP1a and DAPI staining of ctr, G9a-KO, Su(var)3-9-KO, dSetDB1-KD and TM cycle 14 embryos. For dSetDB1-KD and TM cycle 14 embryos, H3K9me3 staining intensity was increased 20 times more than in ctr to see the leftover H3K9me3 signal. *n* is at least 4 embryos per genotype. Scale bars, 10 µm. (**F**) H3K9me2 and DAPI staining of ctr, G9a-KO, Su(var)3-9-KO, dSetDB1-KD and TM cycle 14 embryos. *n* is at least 4 embryos per genotype. Scale bars, 10 µm. (**G**) Violin plot of log2 fold change of H3K9me3 signal on H3K9me3 peaks found in PCH and chr_arms in single mutants: Su(var)3-9-KO and dSetDB1-KD and TM cycle 14 embryos compared to ctr embryos. The boxplot inside indicates interquartile range from 1st (Q1) and 3rd (Q3) quartile, whiskers denote 1.5 times interquartile region (IQR) below Q1 and above Q3. (**H**) Violin plot of log2 fold change of H3K9me2 signal on H3K9me3 peaks found in PCH and chr_arms in single mutants: Su(var)3-9 KO and dSetDB1-KD and TM cycle 14 embryos compared to ctr embryos. The boxplot inside indicates interquartile range from 1st (Q1) and 3rd (Q3) quartile, whiskers denote 1.5 times interquartile region (IQR) below Q1 and above Q3. (**I**) Heatmaps of H3K9me3, and HP1a at cycle 14 clustered into PCH, chr_arms and chr_arms with newly appearing HP1a peaks only in TM. The last column represents the density of repeats at H3K9me3 ZGA peaks. The signal is ±10 kb centered on HP1a peaks at cycle 14 TM embryos and ranked by H3K9me3 signal intensity at cycle 14 ctr. Mean signal of two biological replicates is shown. (**J**) Violin plot of log2 fold change of HP1a signal on H3K9me3 peaks found in PCH and chr_arms in single mutants: Su(var)3-9-KO and dSetDB1-KD and TM cycle 14 embryos compared to ctr embryos. The boxplot inside indicates interquartile range from 1st (Q1) and 3rd (Q3) quartile, whiskers denote 1.5 times interquartile region (IQR) below Q1 and above Q3. (**K**) Western blot for HP1a on different amount of protein extracts from ctr and TM cycle 14 embryos. Lamin, H4 and Ponceau staining are used as loading controls.

 © The Author(s)

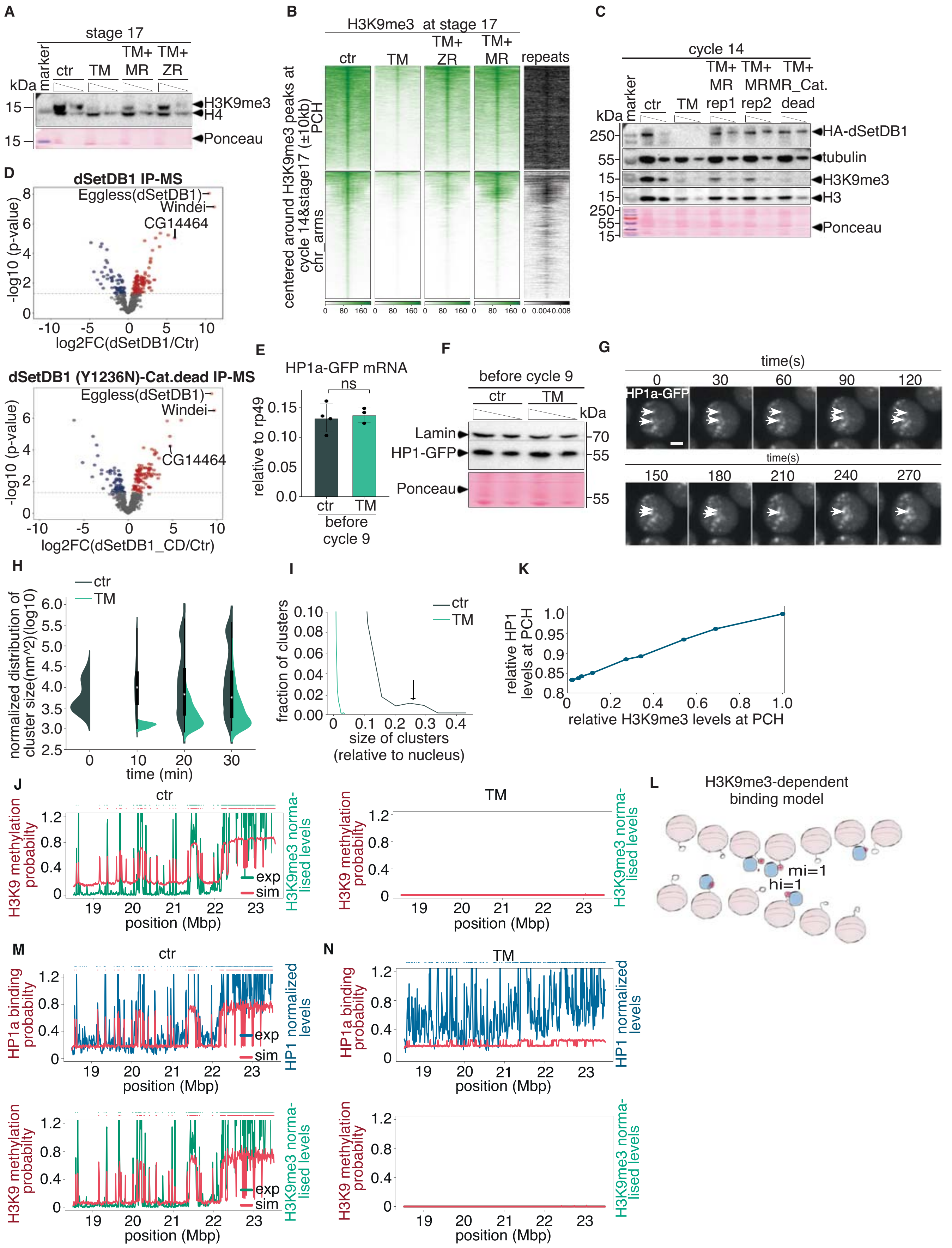

A
stage 17
marker
ctr
TM
TM+ MR
TM+ ZR
kDa
15
15
H3K9me3
H4
Ponceau
B
H3K9me3 at stage 17
ctr
TM
TM+ ZR
TM+ MR
repeats
centered around H3K9me3 peaks at cycle 14&stage17 (±10kb)
PCH
chr_arms
0 80 160
0 0.004 0.008
C
cycle 14
marker
ctr
TM
TM+ MR rep1
TM+ MR rep2
TM+ MR_Cat. dead
kDa
250
55
15
15
250
55
15
HA-dSetDB1
tubulin
H3K9me3
H3
Ponceau
D
dSetDB1 IP-MS
Eggless(dSetDB1)
Windei
CG14464
-log10 (p-value)
log2FC(dSetDB1/Ctr)
dSetDB1 (Y1236N)-Cat.dead IP-MS
Eggless(dSetDB1)
Windei
CG14464
-log10 (p-value)
log2FC(dSetDB1_CD/Ctr)
E
HP1a-GFP mRNA
ns
relative to rp49
ctr
TM
before cycle 9
F
before cycle 9
ctr
TM
kDa
Lamin
HP1-GFP
Ponceau
70
55
55
G
time(s)
HP1a-GFP
0 30 60 90 120
150 180 210 240 270
H
ctr
TM
normalized distribution of cluster size(nm^2)(log10)
time (min)
0 10 20 30
I
ctr
TM
fraction of clusters
size of clusters (relative to nucleus)
K
relative HP1 levels at PCH
relative H3K9me3 levels at PCH
J
ctr
TM
H3K9 methylation probability
H3K9me3 normalised levels
exp
sim
position (Mbp)
L
H3K9me3-dependent binding model
mi=1
hi=1
M
ctr
N
TM
HP1a binding probability
HP1 normalized levels
exp
sim
position (Mbp)
H3K9 methylation probability
H3K9me3 normalised levels

Figure EV5. Lack of H3K9me3 in prior and at ZGA cannot be rescued by zygotic expression of dSetDB1.

(**A**) Western blot for H3K9me3 on different amount of protein extract from stage 17 ctr, TM, zygotic expression of dSetDB1 in the background of TM (TM + ZR) and maternal expression of dSetDB1 in the background of TM embryos (TM + MR). H4 and Ponceau staining are used as loading controls. (**B**) Heatmaps of H3K9me3 at stage 17 embryos clustered into PCH and chr_arms. The last column represents the density of repeats at H3K9me3 ZGA and stage 17 peaks. The signal is ±10 kb centered on H3K9me3 peaks at cycle 14 and stage 17 ctr embryos and ranked by signal intensity at stage 17 ctr embryos. Mean signal of two replicates is shown. (**C**) Western blot for flag-HA-dSetDB1 and H3K9me3 on different amount of protein extract from cycle 14 ctr, TM, maternal expression of wild-type dSetDB1 (TM + MR) and maternal expression of catalytic dead dSetDB1 in the background of TM embryos (TM + MR_cat.dead). H4 and Ponceau staining are used as loading controls. TM + MR is loaded as 2 biological replicates. (**D**) Volcano plot showing the enrichment of proteins in top: endogenously flag-HA-tagged wild-type dSetDB1 and bottom: endogenously flag-HA-tagged catalytic dead dSetDB1(Y1236N) over ctr embryos. Red and blue dots represent enriched and diminished proteins in the flag-HA-tagged IP compared to ctr embryos, respectively. $n = 3$ biological replicates were used for both ctr and wild-type and catalytic dead dSetDB1 IPs. (**E**) HP1a mRNA levels relative to rp49 in ctr HP1-GFP homozygous and TM HP1-GFP homozygous before cycle-9 embryos measured by RT-qPCR (P value $= 0.7247$, two-tailed Welch two-sample t test, for cycle 14 embryo). $n = 3$–4 biological replicates are used for each genotype. Each dot represents one biological replicate. Shown mean +/− s.d. (**F**) Western blot on different amount of protein extract from before cycle-9 ctr and TM embryos that homogenously express endogenously tagged HP1a-GFP. Lamin and Ponceau staining are used as loading controls. (**G**) A single nucleus maximum projection of 30 z-stacks images of endogenously tagged HP1a with GFP for cycle 14 embryos for an interval of 30 s. 10 images correspond to a period of 4.5 min. Two arrows show the two clusters of HP1-GFP that over time fuse into one cluster. Scale bars, 2 µm. (**H**) The distribution of cluster size normalized to nucleus size for ctr ($n = 3$ embryos) and TM ($n = 4$ embryos) cycle 14 embryos. For clarity, only time points 0, 10, 20 and 30 min are shown. (**I**) Cluster size distribution in ctr and TM at late time points (> 30 min). An arrow indicates the population with a bigger size. (**J**) Normalized H3K9me3 profile detected in CUT&Tag (green curve) and predicted by the model where H3K9me3 mediates only HP1-HP1 interactions (red curve) in ctr and TM. Sites with H3K9me3 are shown in the upper part of the panel for the simulation (red track) and in CUT&Tag (blue track, see Methods). See also Dataset EV4. (**K**) Fraction of HP1 bound to PCH relative to the fraction detected with the best model from Fig. 5G as a function of methylation rate. (**L**) A schematic representation of the model where H3K9me3 is important for HP1 binding to the chromatin. Each locus (pink bead) can undergo H3K9 methylation (red circle) and bind HP1 (blue shape). (**M**) Top panel: normalized HP1 binding profile detected in CUT&Tag (blue curve) and predicted by the model where H3K9me3 mediates only HP1 binding to chromatin (red curve) in ctr. Bottom panel: normalized H3K9me3 profile detected in CUT&Tag (green curve) and predicted by the model where H3K9me3 mediates only HP1 binding to chromatin (red curve) in ctr. Bound sites and sites with H3K9me3 are shown in the upper part of the panel for the simulation (red track) and in CUT&Tag (blue track, see "Methods"). (**N**) as in M for TM.

 © The Author(s)
